# Disarming of type I-F CRISPR-Cas surveillance complex by anti-CRISPR proteins AcrIF6 and AcrIF9

**DOI:** 10.1038/s41598-022-19797-y

**Published:** 2022-09-15

**Authors:** Egle Kupcinskaite, Marijonas Tutkus, Aurimas Kopūstas, Simonas Ašmontas, Marija Jankunec, Mindaugas Zaremba, Giedre Tamulaitiene, Tomas Sinkunas

**Affiliations:** 1 Institute of Biotechnology, Life Sciences Center, Vilnius University, Sauletekio Ave. 7, 10257 Vilnius, Lithuania; 2 Department of Molecular Compound Physics, Center for Physical Sciences and Technology, Savanoriu 231, 02300 Vilnius, Lithuania; 3 Institute of Biochemistry, Life Sciences Center, Vilnius University, Sauletekio Ave. 7, 10257 Vilnius, Lithuania

**Keywords:** Biochemistry, Biophysics, Molecular biology, Structural biology

## Abstract

CRISPR-Cas systems are prokaryotic adaptive immune systems that protect against phages and other invading nucleic acids. The evolutionary arms race between prokaryotes and phages gave rise to phage anti-CRISPR (Acr) proteins that act as a counter defence against CRISPR-Cas systems by inhibiting the effector complex. Here, we used a combination of bulk biochemical experiments, X-ray crystallography and single-molecule techniques to explore the inhibitory activity of AcrIF6 and AcrIF9 proteins against the type I-F CRISPR-Cas system from *Aggregatibacter actinomycetemcomitans* (Aa). We showed that AcrIF6 and AcrIF9 proteins hinder Aa-Cascade complex binding to target DNA. We solved a crystal structure of Aa1-AcrIF9 protein, which differ from other known AcrIF9 proteins by an additional structurally important loop presumably involved in the interaction with Cascade. We revealed that AcrIF9 association with Aa-Cascade promotes its binding to off-target DNA sites, which facilitates inhibition of CRISPR-Cas protection.

## Introduction

Clustered Regularly Interspaced Short Palindromic Repeats (CRISPR) and CRISPR-associated (*cas*) genes comprise prokaryotic adaptive immune systems that encounter and dispose of invading nucleic acids (e.g., phages)^1,2^. CRISPR-Cas protection involves three different stages: (i) adaptation, (ii) expression, and (iii) interference^3^. In the adaptation stage, a small DNA fragment (protospacer) of the invader is inserted into the CRISPR region as a new spacer^4^. During the expression stage, the transcript of the CRISPR locus is processed to generate small CRISPR RNA (crRNA) molecules, which together with Cas proteins assemble into a ribonucleoprotein effector complex. In the interference stage, the effector complex detects the crRNA-matching target sequence (protospacer together with the protospacer adjacent motif (PAM)) and then hydrolyses the intruder’s nucleic acid^5,6^. The very diverse CRISPR-Cas systems are partitioned into two classes comprising six types (I-VI), which are further divided into subtypes (A, B, C, etc.)^7,8^.

The evolutionary arms race between prokaryotes and their genetic predators gave rise to small anti-CRISPR (Acr) proteins that act as a counter defence against CRISPR-Cas systems by inhibiting the action of their effector complex^9,10^. The Acrs are classified into families according to the subtype of the targeted CRISPR-Cas system. Currently, combining bioinformatic analysis and functional screening over 100 Acr families have been identified^11^.

The first anti-CRISPR proteins were discovered as inhibitors of the type I-F CRISPR-Cas system in *Pseudomonas aeruginosa* (Pa)^12^. The type I-F Cascade surveillance complex binds a crRNA-matching target sequence forming an R-loop where the spacer basepairs with the complementary strand of protospacer, while the non-complementary strand is displaced as ssDNA. The R-loop attracts Cas2/3 nuclease-helicase, which binds to the displaced strand and starts unidirectional DNA target degradation^13,14^. Currently, twenty-four AcrIF families (AcrIF1-24) have been found that inhibit the interference stage of the prototype Pa-CRISPR-Cas system^12,15–17^. The most common target of the AcrIFs is the “sea-horse” shaped Cascade complex, which is assembled from four Cas proteins and one crRNA molecule with a Cas8f_1_:Cas5f_1_:Cas7f_6_:Cas6f_1_:crRNA_1_ stoichiometry^13,18–20^. The AcrIFs usually bind Cascade complex blocking target DNA binding sites^18–25^. Recently, enzymatic Cascade inactivation was demonstrated^26^. The AcrIFs can also allosterically inhibit cleavage initiation^22^ or DNA hydrolysis^23,27,28^. A rapidly growing number of structural studies reveal principles of the Pa-Cascade complex interplay with different AcrIF families; however, the mechanisms of AcrIF interactions with other type I-F systems remain to be established.

The CRISPR-Cas effector complexes have been adapted for many important applications including genome engineering, gene regulation, and diagnostics^29,30^. However, the uncontrollable action of these powerful molecular tools might cause undesirable genetic alterations limiting their applications. The Acr proteins are natural CRISPR-Cas breaks, which show promise as regulators for the action of the effector complexes^31^. Thus, the biochemical characterisation of these proteins might contribute to the development of new molecular tools.

In this study, we examined the inhibition potential of ten AcrIF families for the type I-F CRISPR-Cas system from *Aggregatibacter actinomycetemcomitans* D7S-1 (Aa). We show that only two AcrIF families, the AcrIF6 and AcrIF9 proteins, blocked the activity of the Aa-CRISPR-Cas in vivo. The biochemical analysis revealed that both AcrIF6 and AcrIF9 interact with the Aa-Cascade complex and interfere with the DNA target recognition; however, AcrIF9 binding to Aa-Cascade also stimulated non-specific DNA binding. We demonstrated the dynamics of the AcrIF9-Cascade complex upon DNA binding by using single-molecule AFM and Soft DNA Curtains techniques. Finally, we solved a crystal structure of Aa1-AcrIF9 and identified putative AcrIF9 surfaces involved in interactions with Aa-Cascade and non-specific DNA.

## Results

### AcrIF6 and AcrIF9 inhibit Aa-CRISPR-Cas in vivo

**Figure 1:**
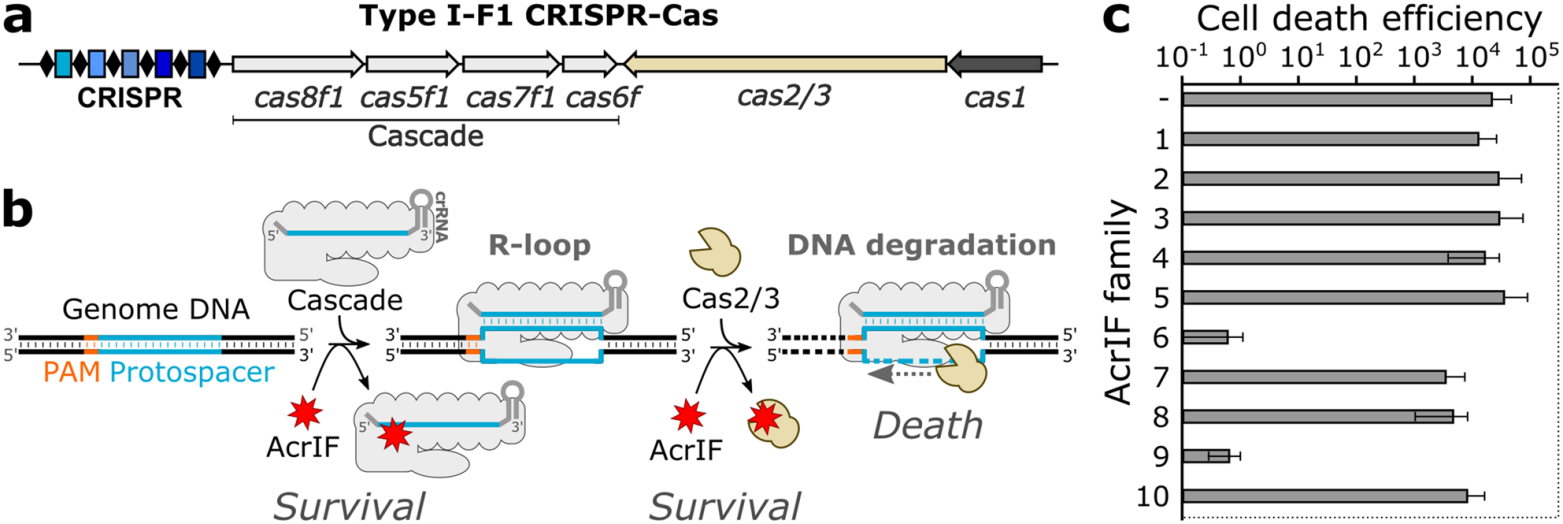
Inhibitors of Aa-CRISPR-Cas system. (**a**) Schematic representation of the Aa-CRISPR-Cas locus composed of six *cas* genes and CRISPR locus. Cascade genes are underlined. (**b**) Rescue assay by AcrIF. The *E. coli* genomic DNA was targeted by the Cascade complex forming the R-loop, which triggered the Cas2/3-mediated DNA degradation leading to cell death. The cell could survive when the AcrIF blocked the action of either Cascade or Cas2/3. (**c**) In vivo inhibition of Aa-CRISPR-Cas by the AcrIFs. Representatives from the AcrIF1-10 families were co-expressed with the Aa-CRISPR-Cas system either targeting or non-targeting the *E. coli* genome. Empty vector (–) was used as a control. The ratio of transformation efficiencies of non-targeting and targeting guides was expressed as cell death efficiencies. Error bars represent standard deviations of average in at least three separate experiments.

Previous studies of the AcrIF activity were focused mainly on the inhibition of the *Pseudomonas aeruginosa* (Pa) CRISPR-Cas system. We wanted to investigate whether these AcrIF proteins are capable to interfere with our model *Aggregatibacter actinomycetemcomitans* (Aa) CRISPR-Cas system (Fig. 1a), which in-depth biochemical analysis was performed previously^14^. It is distinct from the Pa-CRISPR-Cas at the protein sequence level (Supplementary Table S1). At the moment of our research, ten AcrIF families (AcrIF1-AcrIF10) were segregated thus we tested their inhibitory activity in vivo^12,15^ (Supplementary Table S2). We co-expressed AcrIF proteins with the Aa-CRISPR-Cas system, which was guided to target the genome of the recombinant *E. coli* cells. Therefore, cells carrying inactive AcrIF were killed by the Aa-CRISPR-Cas system, while the active AcrIFs protected the host from genome damage (Fig. 1b). In our assay, only the homologues of AcrIF6 (Os and Pa) and AcrIF9 (Aa1, Aa2, Aa3, Vp) families inhibited the action of the Aa-CRISPR-Cas system, while the AcrIF1–AcrIF5, AcrIF7, AcrIF8, and AcrIF10 proteins showed no protection in vivo (Fig. 1c and Supplementary Fig. S1). Next, we purified Os-AcrIF6, Aa1-AcrIF9, Aa2-AcrIF9, Aa3-AcrIF9, and Vp-AcrIF9 proteins (Supplementary Fig. S2) to assess their action in vitro (unless we want to stress particular homologue, for simplicity further in the text we refer to Os-AcrIF6 and Aa1-AcrIF9 proteins as AcrIF6 and AcrIF9, respectively).

### AcrIF6 and AcrIF9 interact with Aa-Cascade

Acr proteins act by targeting various components of the CRISPR-Cas effector complex. In the case of type I-F CRISPR-Cas systems, AcrIFs impede the action of either the Cascade complex or the Cas2/3 nuclease-helicase^9,10^. In principle, Acrs can interrupt the CRISPR-Cas protection in three ways: (i) prevent the DNA target recognition and binding, (ii) disassemble the R-loop, or (iii) hinder DNA degradation. We performed in vitro experiments to delineate, which mechanistic approach is used by the AcrIF6 and AcrIF9 proteins in the case of the Aa-system.

**Figure 2:**
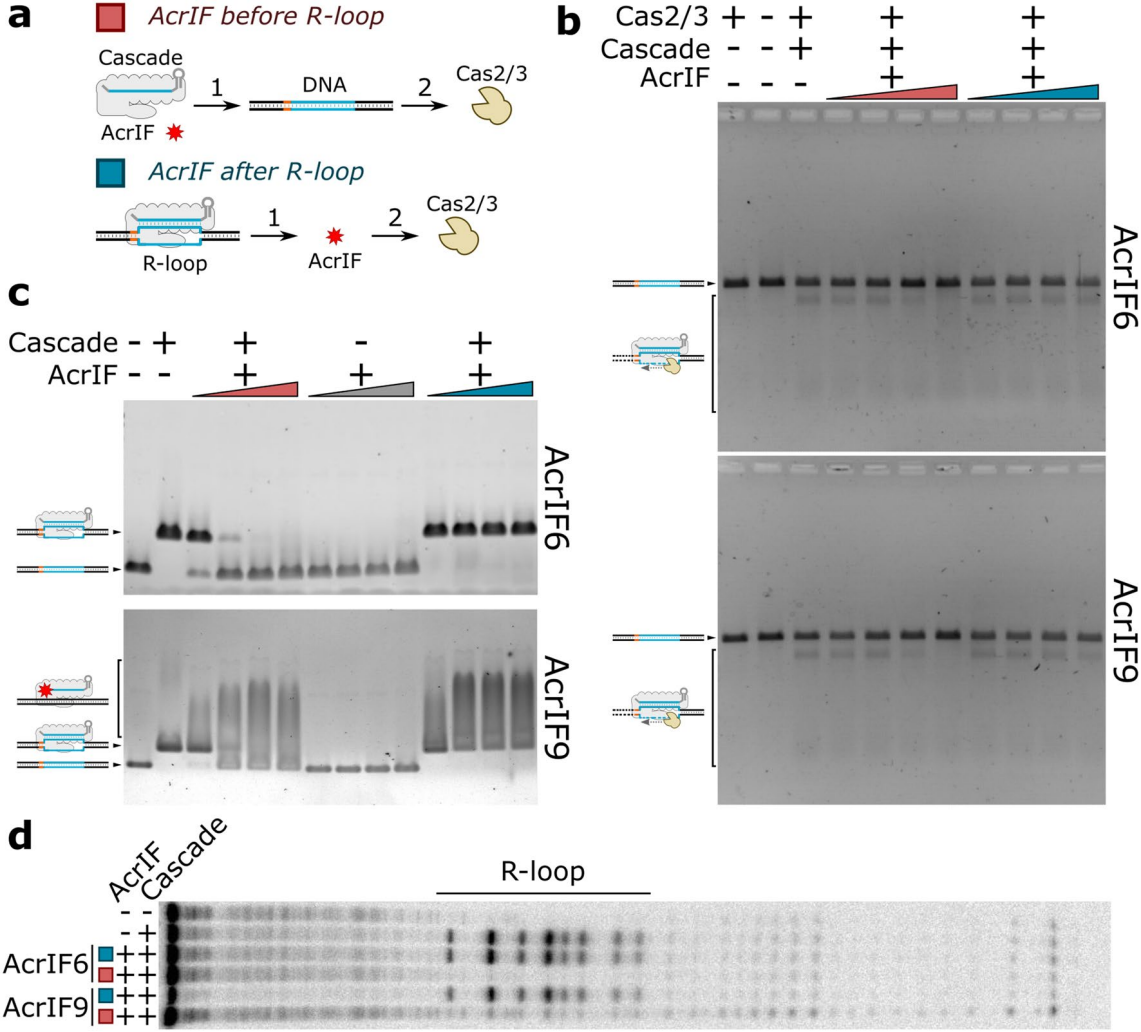
AcrIF6 and AcrIF9 interplay with the Cascade. (**a**) Mixing order of reaction components. AcrIF protein was introduced either before (red-coded) or after (blue-coded) the R-loop formation (1). Then cleavage was initiated by Cas2/3 addition (2). (**b**) The Cas2/3-mediated cleavage. Increasing AcrIF concentrations (5, 50, 500, 5000 nM) were introduced to the cleavage reactions as indicated in (**a**). (**c**) Cascade binding to the DNA target. Increasing AcrIF concentrations (30, 300, 3000, 20,000 nM) were introduced to 20 nM Cascade binding reactions as indicated in (**a**). The binding reactions were assayed by EMSA in agarose gel. See Supplementary Fig. S3 for EMSAs using 100 nM Cascade. (**d**) The footprint of the R-loop in the presence of the AcrIF6 and AcrIF9. Reactions were mixed as indicated in (**a**; 1) then the footprint of the R-loop was initiated by the addition of KMnO_4_. The solid line above the gel indicates the boundaries of the R-loop.

We tested the influence of AcrIFs for the Aa-CRISPR-Cas action before and after the R-loop formation, i.e., AcrIF6/9 was either incubated with the Aa-Cascade complex before DNA addition or mixed with the preformed R-loop (Fig. 2a). First, we monitored the influence of these AcrIFs on the Cascade-Cas2/3 mediated DNA target degradation, which occurs unidirectionally upstream from the R-loop leaving the downstream DNA intact. Both AcrIF6 and AcrIF9 hindered the degradation when they were mixed with Cascade before DNA introduction. Contrary, these AcrIFs had no influence on DNA degradation when the R-loop was already formed (Fig. 2b). Thus, both AcrIF6 and AcrIF9 interfere with the R-loop formation; however, they do not influence the activity of Cas2/3 in the presence of the R-loop.

Next, we employed EMSA to analyze Cascade binding to the target DNA in the presence of the AcrIF6 and AcrIF9 proteins (Fig. 2c and Supplementary Fig. S3). Both AcrIF6 and AcrIF9 hindered the specific interaction of the Cascade with the target DNA as the increasing concentration of the AcrIF6 and AcrIF9 led to unbound DNA. When AcrIF proteins were introduced after R-loop formation no unbound DNA was present in the gel showing that these proteins can’t rip off the Cascade from the preformed R-loop (Fig. 2c). This is in agreement with the footprint of the Cascade where we monitored the interference of the R-loop formation by AcrIF6 and AcrIF9; however, these proteins were incapable to disassemble the preformed R-loop (Fig. 2d). In the presence of AcrIF9 and Cascade, the smeared DNA band above the bands corresponding to the unbound DNA or R-loop appeared. The electrophoretic mobility of the smeared DNA band even decreased by increasing the Cascade concentration (a five-fold increase) (Supplementary Fig. S3). However, AcrIF9 protein in the absence of Cascade didn't change the mobility of the DNA (Fig. 2d). Other AcrIF9 homologues (Aa2, Aa3 and Vp) also showed analogous DNA binding patterns (Supplementary Fig. S4). Moreover, such smeared DNA bands were also observed using DNA that was non-specific for the Cascade, i.e., the Cascade-matching protospacer was absent in the DNA (Supplementary Fig. S3). The non-specific DNA could also displace the AcrIF9-Cascade complex bound to the target DNA (Supplementary Fig. S5). These results indicate that the AcrIF9-Cascade complex can form non-specific interactions with DNA.

Last, to study a possible interaction between Cascade and AcrIF6 or AcrIF9 we investigated protein mixes by the size-exclusion chromatography. Both AcrIF6 and AcrIF9 proteins eluted together with Cascade showing their direct interaction with the complex (Supplementary Fig. S6).

Taken together, both AcrIF6 and AcrIF9 bind to the Cascade complex inhibiting its ability to recognize DNA targets. Additionally, AcrIF9 drives Cascade binding to DNA in a sequence non-specific manner, which we aimed to analyze in more detail.

### Monitoring AcrIF9-Cascade binding to DNA at a single-molecule level

We employed atomic force microscopy (AFM) and Soft DNA Curtains^32,33^ technologies to get mechanistic information on the AcrIF9-Cascade complex binding to a DNA at a single-molecule level.

**Figure 3:**
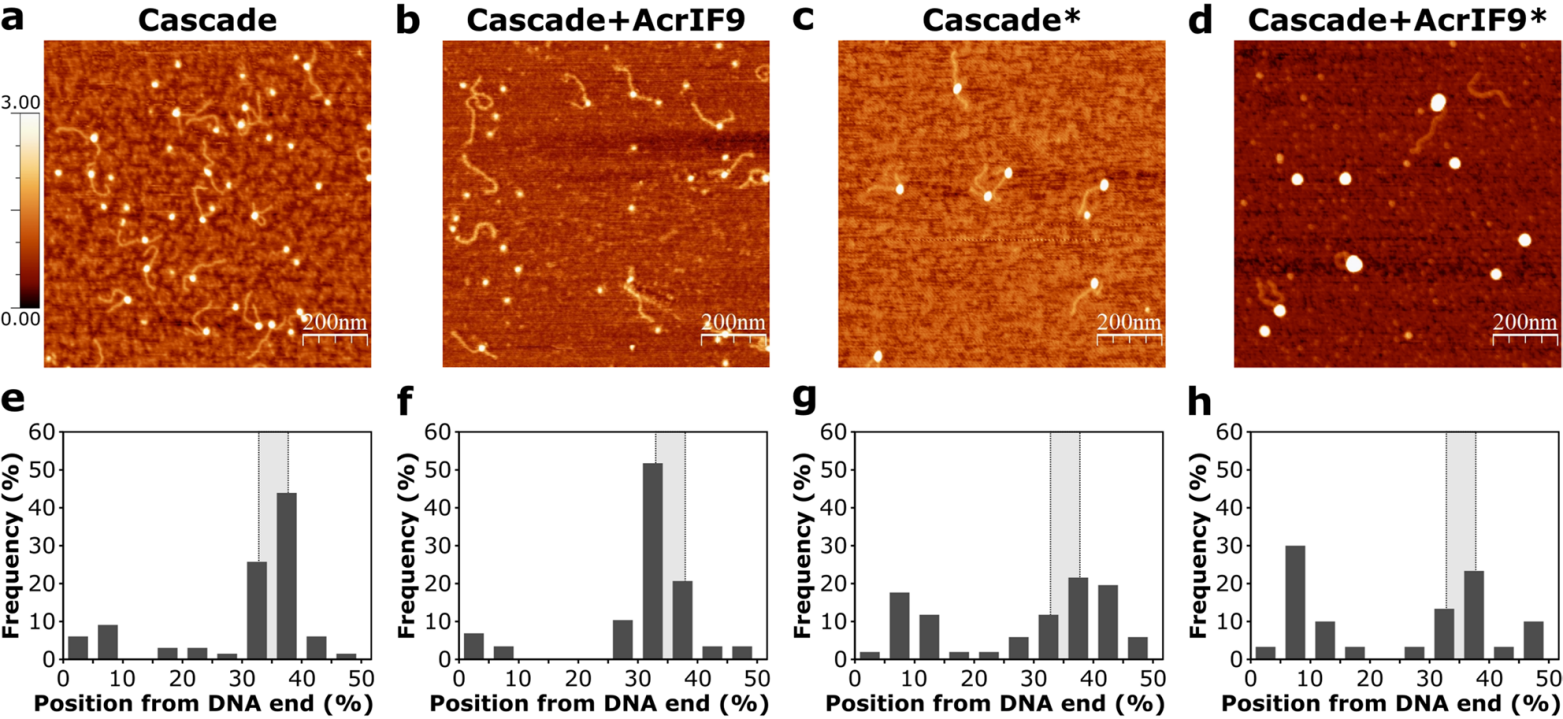
Cascade and AcrIF9-Cascade binding distribution on DNA monitored by AFM. (**a**–**d**) Representative AFM images of the protein-DNA interaction complexes. Scan size 1 µm by 1 μm (additional images and magnified views are provided in Supplementary Figs. S7 and S8, respectively). (**e**–**h**) Protein binding distribution on the DNA. The percentage histogram indicates the frequency of the binding events according to the binding sites, which were obtained by measuring the distance from the centre of a bound protein to the shorter DNA end. (**a**,**e**) Cascade (number of analyzed complexes—n = 66) and (**b**,**f**) AcrIF9-Cascade (n = 29) binding to DNA in the absence of the cross-linker. (**c**,**g**) Cascade (n = 51) and (**d**,**h**) AcrIF9-Cascade (n = 30) binding to DNA in the presence of the cross-linker (2% (v/v) of glutaraldehyde; indicated by asterisk). AcrIF9 and Cascade were preincubated before DNA addition in (**b**,**d**). The Cascade target site is indicated by a grey-coloured interval within the dotted lines. The data are summarized in Supplementary Table S3.

First, we used AFM, a surface-sensitive technique, which enables the detection of protein-DNA interactions at high resolution. We used a DNA fragment of ~ 600 bp length containing the Cascade target that was located ~ 200 and 400 bp from the DNA ends (~ 1/3rd of DNA length). We incubated the DNA with either Cascade or the AcrIF9-Cascade complex and scanned the samples using the AFM. In the absence of AcrIF9, Cascade was localized at the expected 1/3rd of DNA length indicating the specific interaction with the target sequence (Fig. 3a,e and Supplementary Figs. S7, S8). When the AcrIF9-bound Cascade was added, the unbound DNA and the separate protein complexes dominated on the surface (Fig. 3b; the unbound DNA on the surface comprise 63% (n = 227) compared to 3% (n = 210) in the presence and absence of AcrIF9, respectively), confirming that AcrIF9 inhibits the Cascade binding to the target sequence. However, we also observed rare protein binding events located at the 1/3rd of DNA length, which most likely represented the R-loop formed by the AcrIF9-unhindered Cascade (Fig. 3f and Supplementary Figs. S7, S8). The extensive wash steps are performed before AFM imaging dissociating weakly bound biomolecules from the surface thus the less stable interactions of the Cascade-AcrIF9 complexes are most likely washed out from the non-specific DNA sites leaving the more stable R-loops. We tried to fix weak protein-DNA interactions using glutaraldehyde crosslinking. In this case, binding events for both Cascade (Fig. 3c and Supplementary Figs. S7, S8) and the AcrIF9-Cascade (Fig. 3d and Supplementary Figs. S7, S8) complexes were detected which were distributed more evenly throughout DNA length indicating the non-specific binding (Fig. 3g,h, and Supplementary Table S3). However, the addition of glutaraldehyde promotes aggregation (Supplementary Fig. S7) and increases the volumes of the complexes by 2–3 times compared with complexes untreated with the crosslinker (Fig. 3a–d, and Supplementary Table S4). In the control experiment, the AcrIF9 protein in the absence of Cascade failed to crosslink with the DNA (Supplementary Fig. S9), supporting the failure of AcrIF9 to alter DNA migration in the EMSA experiment (Fig. 2c).

**Figure 4:**
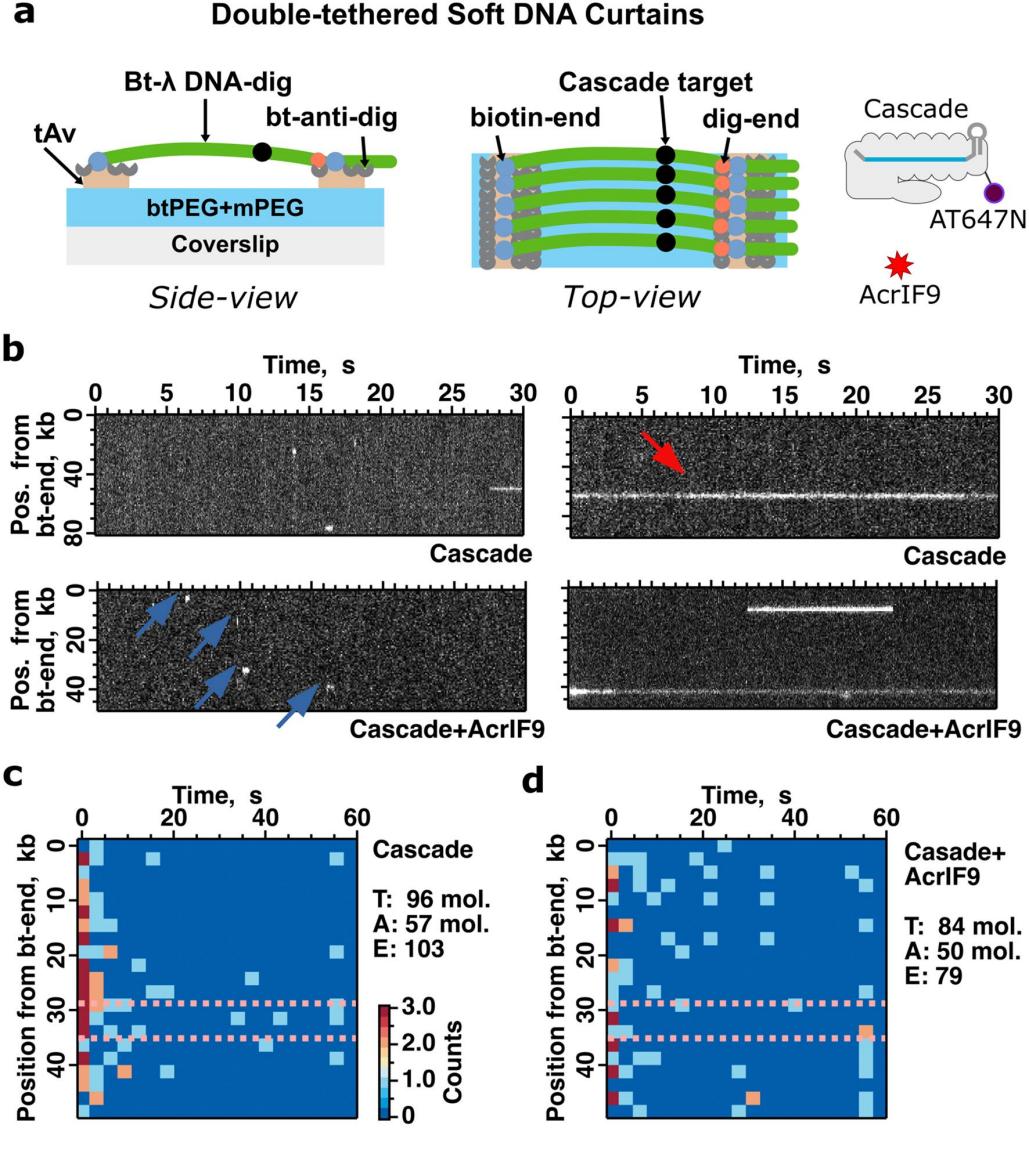
Binding location and duration of Cascade on the DNA Curtains. (**a**) A schematic representation of the assay depicts the double-tethered Soft DNA Curtains with the Cascade target site on λ DNA located at 31.1 kbp from the biotinylated DNA end (bt-end). Biotinylated and digoxigenin labelled λ DNA (bt-λ DNA-dig) was immobilized on the surface printed traptavidin (tAv) line-features. The dig-end of λ DNA was post-tethered using biotinylated anti-dig antibodies (bt-anti-dig). The binding of fluorescently labelled Cascade-mSav-AT647N to DNA was monitored in the presence or absence of AcrIF9. (**b**) Representative kymographs made from individual DNA molecules. These kymographs show short- (left; blue arrows) or longer-lasting (right; red-arrow) binding events of Cascade to the DNA molecule in the absence (top) or presence (bottom) of AcrIF9. (**c**) Cascade binding position vs. dwell time 2D histogram plot. The total number of examined DNA molecules (T) was 96, and 57 DNA molecules showed at least one Cascade binding event (A). The plot represents 103 individual Cascade binding events (E). The colour-code represents the binding counts. (**d**) Cascade pre-incubated with AcrIF9 binding position vs. dwell time 2D histogram plot. The total number of examined DNA molecules was 84, and 50 DNA molecules showed at least one Cascade binding event. The plot represents 79 individual Cascade binding events. The expected range of Cascade targeted binding is marked by the magenta dashed line.

Next, to evaluate the dynamics of Cascade and AcrIF9-Cascade binding to the DNA, we employed the Soft DNA Curtains platform^32,33^. In this stretch-flow assay, oriented bacteriophage lambda DNA molecules (48.5 kb) were tethered at both ends (containing biotin and digoxigenin, respectively) to the flow-cell surface on the printed traptavidin (tAv) line-features (Fig. 4a). The fluorescently labelled Cascade complex (targeting sequence located ~ 31.1 kb from the biotinylated lambda DNA end) was injected into the flow-cell and its binding on the stretched DNA substrate in the absence of the buffer flow was monitored (Fig. 4a). We registered the Cascade binding events (Fig. 4b), which could be grouped into two populations: (i) short-lasting of < 20 s and (ii) long-lasting of > 20 s. The short-lasting events were distributed throughout the DNA length, while the long-lasting events were grouped to the target site (Fig. 4c). Upon addition of AcrIF9, both short and long populations of the Cascade binding were also detected. However, the percentage of events for the long binding population markedly increased compared to the Cascade binding in the absence of AcrIF9 (from ~ 11 to ~ 30%). Furthermore, the long binding events were distributed throughout the DNA length showing the non-specific binding (Fig. 4d). The percentage of the on-target events decreased compared to the Cascade binding in the absence of AcrIF9 (from ~ 18 to ~ 8%). The off-target binding frequency upon addition of AcrIF9 increased from ~ 31 to 42% and the mean off-target dwell-time increased from ~ 7.8 to 18.6 s. Thus, the most likely factor driving the non-specific DNA binding is the increased dwell-time rather than the increased binding frequency of the AcrIF9-Cascade complex at off-target sites. This can explain the similar binding distribution of both crosslinked Cascade and AcrIF9-Cascade complexes in the AFM experiment (Fig. 3g,h) monitoring only fixed time point images.

Taken together the results of the single-molecule studies support previous findings based on the experimental observations in bulk solution: AcrIF9 inhibits the Cascade’s ability to specifically recognize the target site and promotes its non-specific interaction by prolonging the dwell-time of the complex in the off-target DNA sites.

### Structure of AcrIF9

**Figure 5:**
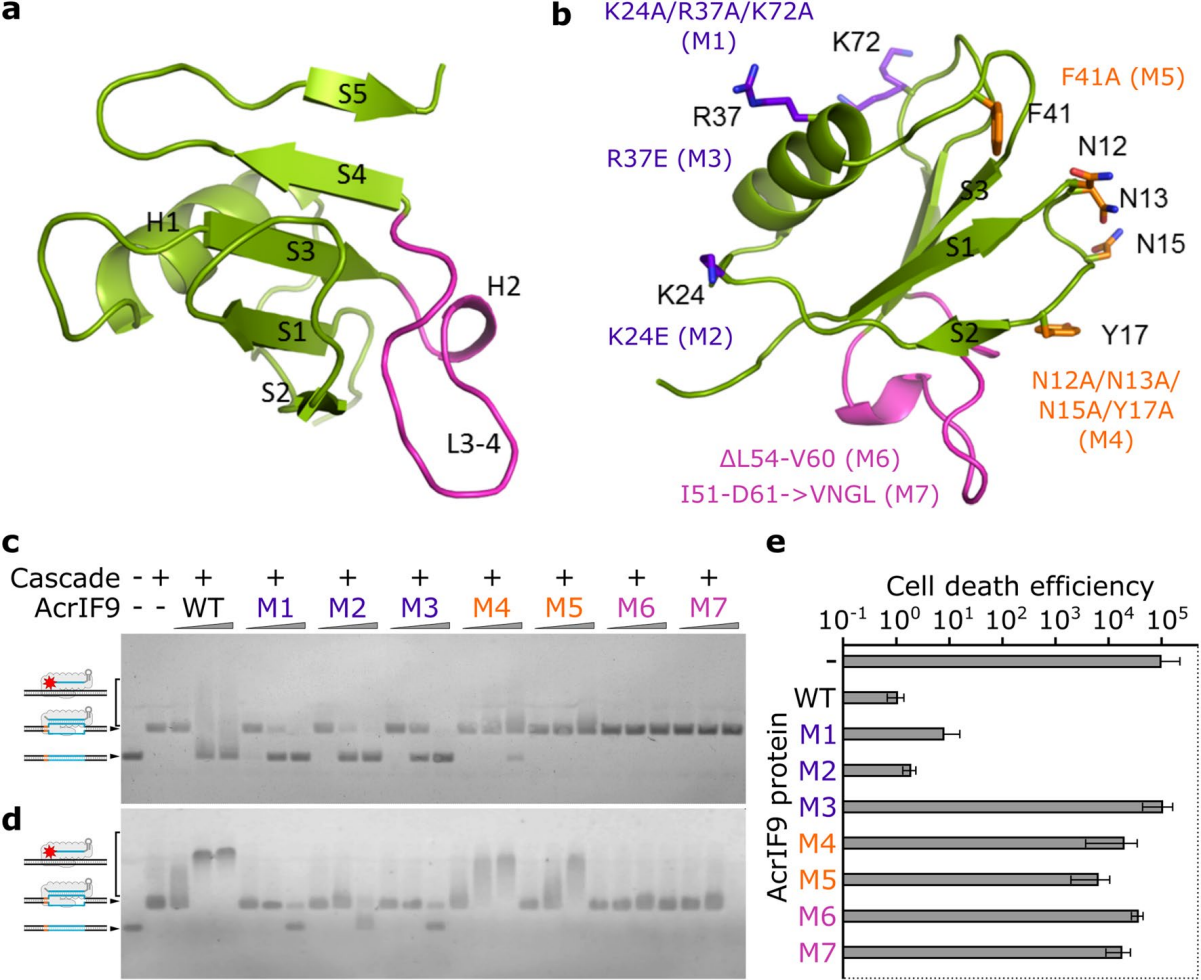
Crystal structure of AcrIF9. (**a**) The overall structure of Aa1-AcrIF9. The secondary structure elements are labelled. (**b**) Cascade and DNA interaction surfaces. Residues of Aa1-AcrIF9 potentially interacting with Cascade and DNA are shown in stick representation and coloured orange and purple, respectively. The loop L3-4 is coloured magenta. Mutants of the corresponding residues are indicated. (**c**,**d**) Cascade binding to the DNA target in the presence of the AcrIF9 mutants. The increasing concentrations of the AcrIF9 mutant proteins (50, 500, 5000 nM) were incubated with either 20 nM (**c**) or 100 nM (**d**) Cascade and then introduced to the binding buffer containing 20 nM target DNA. Protein interactions with the DNA were assayed by EMSA in agarose gel. (**e**) In vivo inhibition of Aa-CRISPR-Cas by the AcrIF9 mutants. The respective AcrIF9 mutants were co-expressed with the Aa-CRISPR-Cas system either targeting or non-targeting the *E. coli* genome. Empty vector (–) was used as a control. The ratio of transformation efficiencies of non-targeting and targeting guides was expressed as cell death efficiencies. Error bars represent standard deviations of average in at least three separate experiments.

We solved the crystal structure of Aa1-AcrIF9 protein at 2.3 Å resolution (Table 1). Aa1-AcrIF9 is composed of a five stranded, antiparallel β sheet cradling an α helix (Fig. 5a). Structure of Aa1-AcrIF9 is similar to three available homologous structures of AcrIF9 from *Pseudomonas aeruginosa* (Pa-AcrIF9, PDB ID 6VQV, Dali Z-score 12.2, 40% identical aa), *Proteus penneri* (Pp-AcrIF9, PDB ID 6W1X, Dali Z-score 10.9, 36% identical aa), and *Photobacterium damselae* (Pd-AcrIF9, PDB 7CHR, Dali Z-score 11.5, 40% identical aa (Pd-AcrIF9 is 100% identical with Pa-AcrIF9)) (Supplementary Fig. S10)^21,34,35^. The main difference of Aa1-AcrIF9 is a longer loop L3-4 (residues 54–62) between the strands S3 and S4, which contains an additional short α helix H2 (Fig. 5a and Supplementary Fig. S10).

**Table 1:** Data collection and refinement statistics.

**Data collection**	
Wavelength	0.9762
Resolution range, Å	54.65–2.3 (2.382–2.3)
Space group	I 4_1_ 2 2
Unit cell	77.285 77.285 75.663 90 90 90
Total/unique reflections	128,129 (13,010)/5338 (508)
Multiplicity	24.0 (25.6)
Completeness (%)	99.59 (99.41)
Mean I/sigma(I)	26.00 (7.98)
Wilson B-factor	31.59
R-merge^a^	0.1014 (0.5107)
CC1/2^b^	0.999 (0.948)
**Refinement**	
Reflections used in refinement (total/R-free)	5322/532
R-work^c^/R-free^d^, %	18.27/22.27
Number of non-hydrogen atoms (macromolecules/solvent)	666 (626/40)
RMS (bonds/angles)	0.003/0.5
Ramachandran: favoured/allowed/outliers (%)	98.65/1.35/0
Rotamer outliers (%)	1.52
Clashscore	3.21
Average B-factor (protein/solvent)	35.52 (35.23/40.03)
Number of TLS groups	1

Data collection statistics for the highest-resolution shell are shown in parentheses.

^a^${\text{R}}_{{{\text{merge}}}} \, = \,\Sigma_{{{\text{hkl}}}} \sum_{{\text{i}}} \left| {{\text{I}}_{{\text{i}}} \left( {{\text{hkl}}} \right)\, - \,\left\langle {{\text{I}}\left( {{\text{hkl}}} \right)} \right\rangle } \right|/\sum_{{{\text{hkl}}}} \sum_{{\text{i}}} {\text{I}}_{{\text{i}}} \left( {{\text{hkl}}} \right)$, where I_i_(hkl) is the intensity of the measured reflection (hkl) and n denotes multiplicity.

^b^CC_1/2_ is the correlation coefficient of the half datasets.

^c^R-work = Σ ||F_obs_| −|F_calc_||/Σ |F_obs_|, where F_obs_ and F_calc_ are observed and calculated structure factors.

^d^R-free = Σ T||F_obs_| −|F_calc_||/Σ T|F_obs_|, where T is a test dataset of ∼ 10% of the total reflections randomly chosen and set aside prior to refinement.

The cryo-EM structures of Pa- and Pp-AcrIF9 bound to the Pa-Cascade complex indicate that two AcrIF9 molecules bind to the thumbs of Cas7f.4 and Cas7f.6 subunits and the two AcrIF9 binding sites are similar^21,34^. The Pp-AcrIF9 also interacts with additional DNA molecules^34^. We superimposed the structures and identified the Aa1-AcrIF9 surfaces potentially involved in the interactions with Cas7f subunits and DNA (Supplementary Fig. S10). We replaced the residues K24A/R37A/K72A (mutant termed M1), K24E (M2), and R37E (M3) that may be responsible for interaction with the DNA. Besides, we introduced mutations N12A/N13A/N15A/Y17A (M4) and F41A (M5) and deleted ΔL54-V60 (M6) or replaced I51-D61 → VNGL (M7; the loop in the Pa-AcrIF9) residues of the L3-4 loop that may be involved in the interactions with the Cas7f subunits (Fig. 5b and Supplementary Fig. S10). We purified the mutant proteins (Supplementary Fig. S11) and assayed their activity in vitro (Fig. 5c,d, and Supplementary Figs. S12, S13) and in vivo (Fig. 5e).

The M1, M2, and M3 mutations disrupted the AcrIF9-mediated binding to non-specific DNA as the smeared DNA bands disappeared in EMSAs with target and non-target DNA (Fig. 5c,d, and Supplementary Fig. S12). However, these mutants retained the ability to block the Cascade binding to the DNA target resulting in inhibition of the DNA degradation by Cas2/3 (Fig. 5c,d, and Supplementary Fig. S13). The M4 and M5 mutations of Aa1-AcrIF9 permitted the Cascade binding to the target sequence leading to unhindered R-loop formation (Fig. 5c) and the Cas2/3-mediated cleavage (Supplementary Fig. S13). Interestingly, these Aa1-AcrIF9 mutants retained the ability to mediate non-specific DNA binding both in target and non-target DNA substrates although with reduced efficiency compared to WT protein (Fig. 5d and Supplementary Fig. S12). This shows that M4 and M5 mutations disturbed the AcrIF9 binding to Cascade; however, the interaction was not diminished. This phenotype may be due to the longer loop L3-4 of Aa1-AcrIF9, which could make additional contacts with the neighbouring Cas7f subunit and stabilize the Cascade-AcrIF9 complex (Supplementary Fig. S10). The deletion of the L3-4 loop (M6) or its replacement with the Pa-AcrIF9 loop (M7) completely blocked the Aa1-AcrIF9 interaction with the Cascade (Fig. 5c,d). However, the stability of these mutants was substantially lower than WT or other Aa1-AcrIF9 mutants (Supplementary Fig. S14). This indicates that the L3-4 loop plays an important structural role within Aa1-AcrIF9 and most likely participates in interaction with the Cascade complex.

Finally, we assayed the potential of these mutant proteins to inhibit the CRISPR-Cas system targeting the *E. coli* genome (Fig. 5e and Supplementary Fig. S15). Alterations within Cascade interaction surface (M4 and M5) and the loop deletions (M6 and M7) completely disrupted the inhibitory ability of AcrIF9 thus enabling the system to kill *E. coli* cells. Mutations of the DNA interaction surface (M1-M3) resulted in different in vivo inhibitory outcomes: M2 had no effect, M1 slightly reduced, and M3 completely abolished the inhibition of the CRISPR-Cas system within the cells. Thus, both Cascade and DNA interaction surfaces play an important role in the AcrIF9 activity in vivo.

## Discussion

The prototype *P. aeruginosa* (Pa) type I-F CRISPR-Cas system was used to validate most of the AcrIF families^12,15–17^. Previously, the in vivo activity of AcrIF1-AcrIF10 was also tested using another type I-F CRISPR-Cas system from *Pectobacterium atrosepticum* (Pe). Almost all AcrIF families, with exception of AcrIF3-AcrIF5, were intercepted with the action of the Pe-CRISPR-Cas^15^. We have recently characterized the DNA interference stage of the type I-F CRISPR-Cas system from *A. actinomycetemcomitans* D7S-1 (Aa)^14^, which is distinct from Pa- and Pe-CRISPR-Cas systems at the sequence level (Supplementary Table S1). The most conserved Aa-Cascade protein is Cas7f, which contains ~ 50% identical amino acids with Pa- and Pe-Cas7f, other Cascade proteins share ~ 30% identical amino acids. We examined whether all AcrIF1-10 proteins are capable to block Aa-CRISPR-Cas action in vivo and found that only homologues of AcrIF6 and AcrIF9 efficiently inhibited Aa-CRISPR-Cas (Fig. 1 and Supplementary Table S2). We assessed the inhibition mechanism conferred by the AcrIF6 and AcrIF9 proteins. We showed that both AcrIF6 and AcrIF9 interact with the Aa-Cascade (Supplementary Fig. S6). These proteins hinder Aa-Cascade binding to the target DNA preventing the R-loop formation and subsequent DNA hydrolysis by the Aa-Cas2/3. However, neither AcrIF6 nor AcrIF9 can disassemble the R-loop or hamper Aa-Cas2/3 activity (Fig. 2). Thus, the AcrIF6 and AcrIF9 families adapted a common strategy of blocking target DNA recognition as exemplified by AcrIF1, AcrIF2, AcrIF7, AcrIF8, AcrIF10, and AcrIF14 proteins^18–23^.

Structural studies of AcrIF binding to Pa-CRISPR-Cas components could clarify the different potency of AcrIF families to inhibit Pa- and Aa-systems. Two copies of both AcrIF1 and AcrIF9 bind to the similar region of the thumb domains of Cas7.4f and Cas7.6f subunits (the same binding sites are observed also for the AcrIF14), which are the most conserved between Pa- and Aa-Cascades (Supplementary Fig. S16)^20–22,34,36^. However, only AcrIF9 acts as an inhibitor of the Aa-Cascade in vivo. AcrIF1 is larger than AcrIF9 and makes additional contacts also with neighbouring Cas7f.3/5 subunits and Cas8f. Probably these additional interactions cannot be formed by AcrIF1 with Aa-Cascade. Members of AcrIF2, AcrIF6, AcrIF7, and AcrIF10 families bind to the DNA binding surface of Cas8f interfering with PAM recognition^18,19,21,22,24,25^. However, differently from AcrIF2, AcrIF7, and AcrIF10, only AcrIF6 inhibits Aa-Cascade. AcrIF6 also interacts with a more conserved Cas7f subunit and Cas5f (Supplementary Fig. S16)^21^. AcrIF4 and AcrIF8 (which do not inhibit Aa-system) mainly bind to Cas8f and Cas5f subunits of Pa-Cascade (Supplementary Fig. S16), which share little sequence similarity with analogous subunits of Aa-Cascade^21,22^. AcrIF3 (inactive against Aa-system) interacts with nuclease-helicase Pa-Cas2/3 protein^27,28^. The homology model of Aa-Cas2/3 revealed that the protein compared to Pa-Cas2/3 has additional structural elements (longer loops 219–229, 247–256 and an additional hairpin structure 1018–1029) at the AcrIF3 binding interface that could prevent its binding (Supplementary Fig. S16). Thus, AcrIF6 and AcrIF9 families target the surfaces of the type I-F effector complexes that are conserved between these systems.

The AcrIF6 acts by blocking the first stage of target recognition by Aa-Cascade. AcrIF6 binding to Cas8f and Cas7f clogs the DNA passage cleft where the PAM sequence is captured and the target sequence complementarity check for the crRNA spacer is initiated. Besides inhibition of target DNA binding, the AcrIF9 protein mediates Aa-Cascade binding to DNA irrelevant from the target sequence (Supplementary Figs. S3 and S5). The positively charged surface can be observed within the structure of the Aa1-AcrIF9 protein. Mutational analysis of this surface proved that it is the binding site for the non-specific DNA (Fig. 5). Similar findings were observed for the Pa-Cascade and Pp-AcrIF9 complex where the DNA backbone is positioned to the analogous surface of the Pp-AcrIF9 subunits^34,37^. We also confirmed the surfaces of the Aa1-AcrIF9 that are responsible for interactions with the Aa-Cascade (Fig. 5). Although the structure of Aa1-AcrIF9 is similar to three available homologous structures of Pa-AcrIF9, Pp-AcrIF9, and Pd-AcrIF9^21,34,35^, it has a unique loop, which could potentially be used for the additional interaction with the Cascade and is important for the structural stability of the Aa1-AcrIF9 (Fig. 5 and Supplementary Fig. S16).

We employed single molecule techniques to analyse the profile and dynamics of the AcrIF9-Cascade binding to the DNA. AFM and Soft DNA Curtains technologies show fixed Cascade binding to the expected target site, while the AcrIF9-Cascade distribute along DNA length with a reduced target site binding (Figs. 3 and 4). Using the Soft DNA Curtains, we detected short- and long-lasting populations of binding events for both Cascade and AcrIF9-Cascade (Fig. 4). The short-lasting binding events for the Cascade distribute throughout the length of the DNA molecule and can be interpreted as target site search, while the long-lasting events populate target site boundaries representing the formed R-loop. The major difference upon the addition of the AcrIF9 is the increased distribution of long-lasting events on off-target DNA sites. The frequencies of off-target binding events are comparable for both AcrIF9-Cascade and Cascade. Thus, the increase of dwell-time at off-target sites most likely is the primary cause driving the non-specific DNA binding of AcrIF9-Cascade, which was monitored as smeared DNA band in the EMSA (Fig. 2). The AcrIF9 might have some DNA sequence preference prolonging AcrIF9-Cascade retention at respective DNA sites. However, AcrIF9-Cascade and DNA interaction is dynamic compared with the R-loop formation and can readily dissociate as seen in AFM without a crosslinker (Fig. 3) and the experiment using concurrent DNA (Supplementary Fig. S5).

The biggest question is the functionality of this non-specific DNA binding by the AcrIF9-Cascade complex since AcrIF9 binding to the Cascade is sufficient to inhibit DNA degradation (Fig. 2 and Supplementary Fig. S13). One explanation could be that the AcrIF9 evolved from a DNA binding protein and the DNA binding surface is evolutionary remains, which have no function in the AcrIF9. This is unlikely as distant AcrIF9 homologues retained this surface (Supplementary Fig. S10). Furthermore, mutation of this surface has a negative effect on the activity of Aa1-AcrIF9 in cells indicating the functional importance of this surface (Fig. 5). Likewise, reduced inhibitory activity was also observed for the DNA binding mutant of Pp-AcrIF9 against Pa-Cascade in vivo^37^. Thus, the AcrIF9-mediated binding to DNA might add an additional layer to Cascade inhibition retaining Cascade in the off-target DNA sites for prolonged times. A recent study revealed that a two-domain AcrIF14 can also mediate non-specific DNA binding. Its C-terminal domain binds to the same surface of Cas7f as AcrIF9, while the N-terminal domain facilitates non-specific DNA binding to the PAM recognition site of Cas8f^36^. The DNA interaction domains are also found in AcrIIA1 and AcrIIA13-AcrIIA15 proteins. The conserved N-terminal HTH domains of these proteins do not participate in the direct inhibition of the effector complex; however, they bind the promoter of the AcrAII genes and play a role in the regulation of its expression^38,39^. Likewise, AcrIF9-Cascade might also have some sequence specificity, which in the natural host could be associated with repression of gene transcription or somehow interfere with cell proliferation.

**Figure 6:**
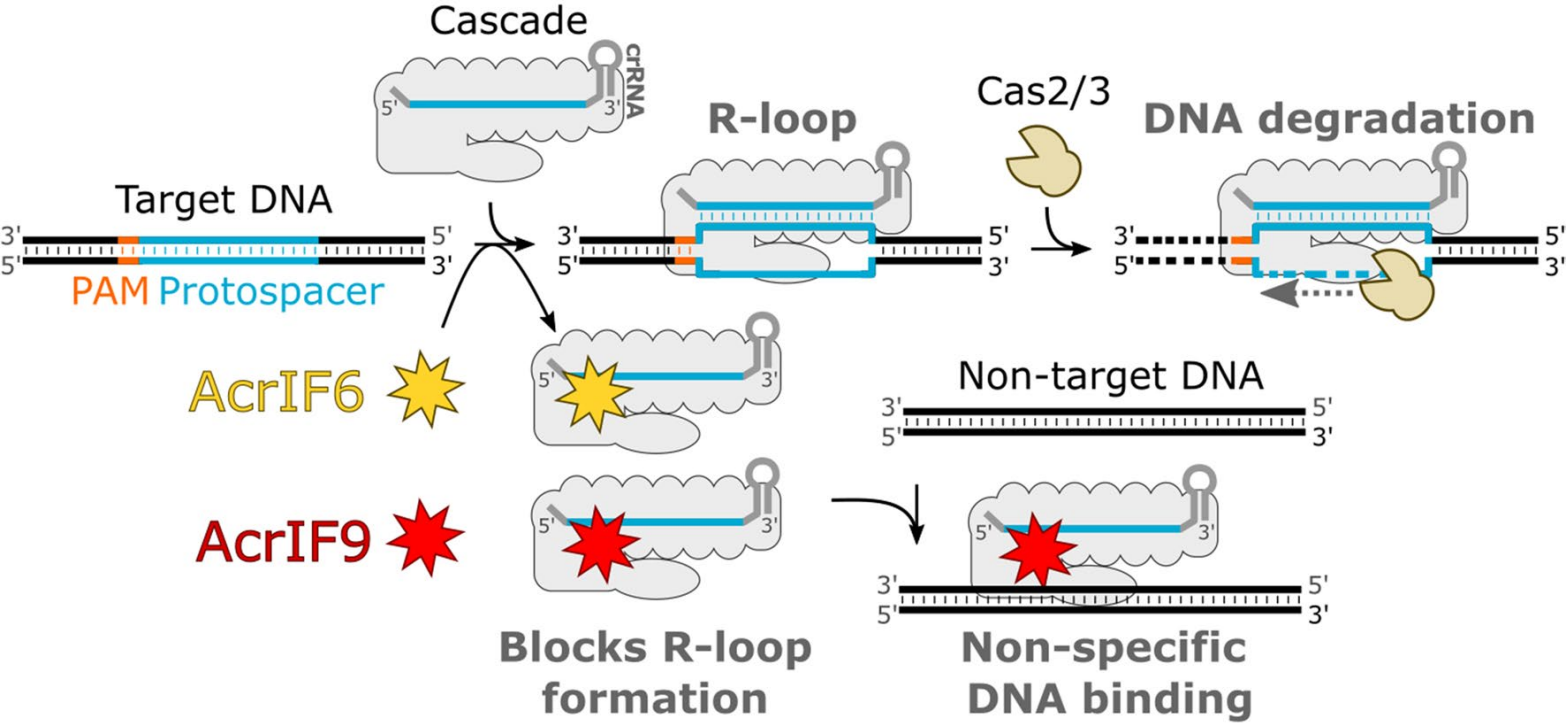
Inhibition mechanism of CRISPR-Cas system by AcrIF6 and AcrIF9 proteins. The Cascade complex guided by crRNA binds the target DNA forming the R-loop, which triggers Cas2/3-mediated target DNA hydrolysis. Both AcrIF6 and AcrIF9 bind the Aa-Cascade complex inhibiting the target DNA binding and R-loop formation. In addition, the AcrIF9 mediates the Aa-Cascade binding to non-specific DNA sequences.

To summarize, we demonstrate that AcrIF6 and AcrIF9 proteins bind to the conserved surfaces of the Cascade and hinder its binding to the DNA target. We also provide evidence for the AcrIF9-mediated non-specific DNA binding (Fig. 6), which facilitates CRISPR-Cas inhibition. Multiple recent studies show the potential of type I CRISPR-Cas systems as molecular tools for the engineering of eukaryotic genomes^40–44^. Thus, anti-CRISPR proteins might become useful regulators balancing the action of type I engineering tools.

## Methods

### Cloning, expression and purification of proteins

Cascade complex and Cas2/3 protein were obtained as described previously^14^. All plasmids used in this study are provided in Supplementary Table S5.

Synthetic DNA fragments of *acrIFs* were obtained from Twist Bioscience (sequences of cloned genes are provided in Supplementary Table S6). The fragments were inserted into either NcoI/XhoI or BamHI/XhoI sites of pCDF-HS expression vector obtaining AcrIF proteins either without tag or fused to N-terminal His_6_-tag, respectively (Supplementary Table S5).

*Escherichia coli* BL21-AI strain containing AcrIF expression vector was grown in LB medium supplemented with 50 μg/mL streptomycin until it reached an optical density of ~ 0.5 (OD_600_). Then protein expression was induced for 4 h at 37 °C by the addition of 1 mM IPTG (isopropylthiogalactoside) and 0.2% (w/v) arabinose. Cells were collected by centrifugation and resuspended in buffer containing 20 mM Tris–HCl (pH 7.0), 500 mM NaCl, 5 mM imidazole, 5 mM 2-ME (2-mercaptoethanol) and 1 mM PMSF (phenylmethylsulfonyl fluoride). Cells were lysed by sonication and cell debris was removed by centrifugation. The resulting supernatant was loaded on a Ni^2+^-charged HiTrap column (GE Healthcare) and eluted with a linear gradient of increasing imidazole. The fractions containing AcrIF were pooled and loaded on Superdex 200 (HiLoad 16/600; GE Healthcare) column for separation by gel filtration. The fractions containing AcrIF were dialysed into 20 mM Tris–HCl (pH 8.0), 300 mM NaCl, 50% (v/v) glycerol, 2 mM DTT (dithiothreitol) and stored at − 20 °C. The concentrations of AcrIFs were measured by UV (280 nm) absorbance.

Cascade complex fused with AviTag at the N-terminus of the Cas6f was expressed in *E. coli* BL21-AI strain. The cells were transformed with (i) pAvi-His-Cd containing Cascade cassette (*cas8f1-cas5f1-cas7f1* and *cas6f1* fused with AviTag-His_6_ sequence at the N-terminal), (ii) pCR-λ containing λ DNA targeting spacer and (iii) pACYC-BirA encoding biotin ligase, which transfers biotin to AviTag sequence (Supplementary Table S5). The cells were grown in LB medium supplemented with 25 μg/mL streptomycin, 15 μg/mL kanamycin, 17 μg/mL chloramphenicol and 50 μg/mL d(+)-biotin. Protein expression was induced with 1 mM IPTG and 0.2% (w/v) l-arabinose for 4 h at 37 °C once a culture reached an OD_600_ of 0.9. Cells were collected by centrifugation, lysed by sonication and cell debris was removed by centrifugation. The Cascade complex was purified as described previously^14^. The fractions containing Cascade complex were dialysed into 20 mM Tris–HCl (pH 8.0), 300 mM NaCl, 50% (v/v) glycerol, 2 mM DTT and stored at − 20 °C. The biotinylated Cascade complex was used for labelling with monovalent streptavidin conjugated with ATTO647N fluorescent dye (mSav-AT647N; see method “Cascade labelling and monitoring on soft DNA curtains”).

### Plasmid interference assays

Electrocompetent *E. coli* BL21-AI cells bearing a plasmid with Cas proteins (pCd-C1-2/3) and either an empty (pCDF-HS) or AcrIF expression vector (pCDF-AcrIF1-10) were transformed with 0.1 μg of either pCR-T (spacer targeting *E. coli* genome) or pCR-NT (non-targeting spacer) plasmids (Supplementary Table S5). Ten-fold serial dilutions of the transformed cells were plated on LB-agar medium containing 0.1 mM IPTG, 0.2% (w/v) l-arabinose, kanamycin (15 μg/mL), streptomycin (25 μg/mL) and chloramphenicol (17 μg/mL) and incubated for 16 h at 37 °C. The survival efficiency of the cells in the presence of respective AcrIF was calculated as a ratio of the pCR-NT and pCR-T plasmid transformation efficiencies. The survival efficiencies are provided as an average of three biological replicas.

### Electrophoretic mobility shift assay (EMSA)

Two mixing approaches of component were used to conduct EMSA: (i) 30 nM or 100 nM of Cascade complex was pre-bound with 20 nM DNA substrate (SP1 or NS; Supplementary Table S7) for 10 min forming the R-loop then AcrIF6, AcrIF9 or respective AcrIF9 mutant (30 nM, 300 nM, 3000 nM, 20,000 nM) was added and incubated for additional 20 min; (ii) 30 nM or 100 nM of Cascade complex was pre-incubated with AcrIF6, AcrIF9 or respective AcrIF9 mutant (30 nM, 300 nM, 3000 nM, 20,000 nM) for 10 min then mixed with 20 nM DNA substrate (SP1 or NS) and incubated for additional 20 min. The incubations were performed at 37 °C or 22 °C (in the assay with AcrIF9 mutants) in 1× TAE (Invitrogen) buffer supplemented with 200 mM NaCl, 10% (v/v) glycerol and 0.1 mg/mL BSA (bovine serum albumin). The samples were analysed on 1% (w/v) agarose gel and visualised by SYBR-Gold (Invitrogen) staining.

### R-loop footprinting experiments

The R-loop was assayed by two mixing approaches of component: (i) the Cascade was mixed with the DNA target forming the R-loop then AcrIF was introduced; (ii) AcrIF was pre-incubated with Cascade before adding the DNA target. The binding was conducted at 37 °C for 20 min in a buffer containing 1× TAE, 200 mM NaCl, 10% glycerol, 0.1 mg/mL BSA, 10 nM SP* substrate ^32^P-5′-end-labelled on the non-target strand (Supplementary Table S7), 100 nM of Cascade and 20 µM AcrIF6 or AcrIF9. Further, KMnO_4_ was added to the final concentration of 2 mM. After incubation at 37 °C for 20 s, the reactions were terminated by the addition of 0.5 M 2-ME and 0.75 M sodium acetate (pH 7.0). Nucleic acids were isolated by phenol–chloroform extraction and precipitated by sodium acetate/isopropanol, followed by the addition of 1 M piperidine and incubation for 30 min at 90 °C. The reaction products were precipitated by sodium acetate/isopropanol and solubilized in loading dye (95% (v/v) formamide, 0.5 mM EDTA (ethylenediaminetetraacetic acid), 0.025% (w/v) bromophenol). The products were separated on a denaturing 20% polyacrylamide gel and visualized by autoradiography.

### Cas2/3 cleavage assay

Prior to Cas2/3 cleavage initiation, the reaction components were mixed by two approaches: (i) the Cascade was mixed with the DNA target forming the R-loop then AcrIF was introduced; (ii) AcrIF was pre-incubated with Cascade before adding the DNA target. The reactions conducted at 37 °C in a buffer containing 20 mM HEPES (pH 7.2), 120 mM NaCl, 10% glycerol, 0.1 mg/mL BSA, 2 mM CoCl_2_, 2 mM ATP, 5 nM SP2 substrate (Supplementary Table S7), 20 nM Cascade, 200 nM Cas2/3 and AcrIF6, AcrIF9 or respective AcrIF9 mutant (5, 50, 500, and 5000 nM). After incubation for 1 h, the reactions were terminated by adding 3× stop solution (75 mM EDTA, 0.025% (w/v) OrangeG, 0.5% (w/v) SDS (sodium dodecyl sulphate), 50% (v/v) glycerol (pH 8.0)) followed by heating at 75 °C for 10 min. Nucleic acids were separated from proteins by phenol–chloroform extraction. The reaction products were analysed on 0.8% (w/v) agarose gel and visualised by ethidium bromide staining.

### SEC pull-down assay

Cascade complex was incubated with respective AcrIFs at the molar ratio of 1:100 in 1× TAE buffer at 37 °C for 30 min. The samples were fractionated by Superdex 200 (HiLoad 16/600; GE Healthcare) column. Proteins in the collected fractions were precipitated using TCA (trichloroacetic acid) then analysed by SDS-PAGE and visualised by Coomassie blue staining.

### Competitive DNA binding

Two mixing approaches of components were used to assay competitive DNA binding: (i) the Cascade was mixed with the DNA target forming the R-loop then AcrIF9 together with competing DNA was introduced; (ii) AcrIF9 was pre-incubated with Cascade before adding the DNA target mixed with competing DNA. The binding reactions were conducted at 37 °C in binding buffer (1× TAE buffer (Invitrogen), 200 mM NaCl, 10% (v/v) glycerol and 0.1 mg/mL BSA) supplemented with 100 nM Cascade, 20 µM AcrIF9, 20 nM SP* target DNA (Supplementary Table S7) and 0.05 nM, 0.5 nM or 5 nM of competitive λ DNA (~ 48 kbp). The samples were analysed on 1% (w/v) agarose gel and visualised by autoradiography.

### Protein stability

The stability of the Aa1-AcrIF9 and its mutants were assayed by nanoscale differential scanning fluorimetry (Prometheus, Nanotemper). The capillaries were loaded with protein samples of 0.5 mg/ml concentration in 20 mM Tris–HCl (pH 8.0), 300 mM NaCl, 25% (v/v) glycerol. The denaturation curves were recorded increasing temperature from 15 to 95ºC at a 1 ºC/min rate. The onset and inflection point temperatures were determined from three separate measurements.

### Protein crystallization and structure determination

The crystals of Aa1-AcrIF9 were obtained by sitting drop vapour diffusion method at 19 °C by mixing Aa1-AcrIF9 (10.7 mg/ml) with the reservoir solution containing 0.1 M Tris–HCl (pH 8.5), 3.0 M NaCl in 1:1 ratio. For data collection, the crystals were shortly dipped into 2.9 M Na-malonate (pH 7.0) and flash-freezed. The X-ray diffraction dataset was collected at the EMBL/DESY Petra III P13 beamline (Germany) at 100 K. XDS^45^, SCALA and TRUNCATE^46^ were used for data processing. The data collection and refinement statistics are presented in Table 1. Homology model of Aa1-AcrIF9 prepared by SWISS-MODEL server (https://swissmodel.expasy.org/)^47^ using Pa-AcrIF9 (PDB ID 6VQV chain A) as a template was used for molecular replacement in MOLREP^48^. Manual model rebuilding of the models was performed in COOT^49^ and the structure was refined with phenix.refine-1.12-2829^50^. All molecular scale representations were prepared using Pymol^51^.

### Sample preparation and AFM imaging of the dry sample

Prior to AFM imaging DNA-Cascade complexes were formed by incubating the SP3 DNA substrate (638 bp, 5 nM; Supplementary Table S7) with Cascade (ratio 1:1) for 10 min at room temperature in the Binding Buffer (20 mM Tris–HCl pH 8.0), 100 mM NaCl) in a total volume of 50 μl. For the AcrIF9-mediated Cascade binding experiments, Cascade and AcrlF9 mixture (1:20, 50 nM:1 μM) was incubated for 5 min then DNA was added and incubated for 10 min. Additionally, the DNA–protein complexes were cross-linked with 2% (v/v) glutaraldehyde for 20 min.

After a tenfold dilution, the reaction solution was spread on the chemically modified mica (grade IV, SPI supplies Inc., USA) at room temperature. 1-(3-Aminopropyl)silatrane-functionalized mica (APS-mica) was used as a substrate for the binding of protein and DNA molecules. APS-mica was prepared as described by^52^. 50 μl of DNA–protein complex solution was deposited on APS-mica for 5 min. After incubation, the mica surface was flushed with excess water and then dried under a flow of nitrogen. The images were acquired in the air with the DimensionIcon (Bruker, Santa Barbara, CA) microscope system in tapping mode. Probes with nominal spring constants of ~ 5 or 40 N/m (FESP or TESP (Bruker)) were used. Typically, the image acquisition was at a speed of 0.4–0.6 Hz and a resolution of 1024 × 1024 pixels, scan size 2 µm × 2 µm. For each reaction, at least three mica samples were prepared, and for each, at least 5–7 images from different locations were captured. Individual protein-DNA complexes with a well-defined structure were manually picked using WsXM software (v4.0 beta 9.3)^53^ for distribution analysis. The protein position from the closer DNA end was determined after drawing a profile line over the entire length of the DNA molecule. Then the protein position was divided by the total length of the DNA molecule.

### Cascade labelling and monitoring on soft DNA curtains

Soft DNA Curtains were prepared using 13-µm spaced line-features containing Si-master as described in^32,33^. Thus the end-to-end distance of the immobilized DNA molecules on traptavidin line-features was ~ 13 µm and that represented ~ 80% tension. For more details see supplementary methods “Glass surface silanization and PEGylation” and “Protein nanopatterning by lift-off µCP and the Soft DNA Curtains”. The DNA binding events by the labelled Cascade complex were monitored using a home-build TIRF (total internal reflection fluorescence) microscopy setup^32^, which is described in supplementary methods “TIRF microscopy”.

Avi-tag containing Cascade complex (Cascade-avitag) was conjugated with the monovalent streptavidin labelled with ATTO647N (mSav-AT647N), which was produced according to^54^. For a detailed description of the monovalent streptavidin production see the Supplementary method “Expression and purification of traptavidin and monovalent streptavidin”. Conjugation reaction mix in buffer (40 mM Tris–HCl, (pH 8.0), 133 mM KCl): 1.7 nM of Cascade-avitag + 17 nM mSav-AT647N + 0.2% (v/v) Tween-20, was incubated for 30 min at the RT. Next, the reaction mix was centrifuged for 5 min at 20,000*g* and the supernatant was removed. The biotin-functionalized magnetic beads (B000-18-2, Rockland) were added to the supernatant (18 µL to 290 µL of supernatant) to remove the excess of mSav-AT647N and incubated for another 30 min under shaking. Next, we performed separation using a magnetic holder and to quench any remaining free mSav-AT647N we added 9 µM of biotin into the separated solution and incubated at least 10 min before use.

The Cascade-mSav-AT647N was diluted to final a concentration of 0.8 nM and 0.4 nM of SG (SYTOX green) was added. This mixture was injected into the flowcell for TIRF microscopy. Then 10 image-long movie was recorded under 488 nm excitation to located DNAs and then 600 image-long movie was recorded under 635 nm excitation. We performed imaging in several different positions of the surface. Then we injected Cascade-mSav-AT647N, which was pre-incubated with AcrIF9 (100× excess) and the same movie acquisition was performed.

### Cascade binding location and duration characterization

Binding location characterization was done using a custom-written automated procedure. It fits each Cascade-mSavAT647N complex in the original images (red-fluorescent spot in TIRF images acquired under 635 nm wavelength excitation) to the 2D Gaussian function with the help of the detection of clusters of interconnected pixels that have values above the manually-set threshold. Thus first fluorescent spots are detected using thresholding of the original image and analysis of more than 4 interconnected pixel-area clusters that have intensity values above the threshold. Only then the 2D gaussian fitting is performed for those preliminary detected spots. Both centre coordinates (x and y) were recorded for each detected fluorescent spot that had the 2D Gaussian fitting error of all parameters < 60% from the parameter value. After fitting, we manually examined the fitted data and selected algorithm-suggested time and X–Y space interconnected fit points (stable Cascade binding events) that lasted for more than 5 frames (i.e.duration at least 0.5 s). This analysis allowed us to extract Cascade binding state durations (i.e. dwell times) and correlate them with the position on the DNA substrate. All procedures were written using Igor Pro (Wavemetrics, Inc.) and are available upon direct request to the authors.

## Supplementary Information

Supplementary Information.

## Data Availability

Atomic coordinates and structure factors for the reported crystal structure have been deposited in the Protein Data bank (https://www.rcsb.org/) under accession number 7BB5.
