# Supplementary material for: Disarming of type I-F CRISPR-Cas surveillance complex by anti-CRISPR proteins AcrIF6 and AcrIF9
Source: Sci Rep. 2022 Sep 15;12:15548. doi: 10.1038/s41598-022-19797-y (PMC9478129; doi:10.1038/s41598-022-19797-y)

Supplementary information for:

Disarming of type I-F CRISPR-Cas surveillance complex by anti-CRISPR proteins AcrIF6 and AcrIF9

Egle Kupcinskaite[1], Marijonas Tutkus[1,2], Aurimas Kopūstas[1,2], Simonas Ašmontas[1], Marija Jankunec[3], Mindaugas Zaremba[1], Giedre Tamulaitiene[1], and Tomas Sinkunas[1*]

[1]Institute of Biotechnology, Life Sciences Center, Vilnius University, Sauletekio al. 7, LT-10257, Vilnius, Lithuania

[2]Department of Molecular Compound Physics, Center for Physical Sciences and Technology, Savanorių 231, Vilnius LT-02300, Lithuania.

[3]Institute of Biochemistry, Life Sciences Center, Vilnius University, Sauletekio al. 7, LT-10257, Vilnius, Lithuania

[*] To whom correspondence should be addressed: tomas.sinkunas@bti.vu.lt

Supplementary methods:

Chemicals and Materials. Silicone elastomer Sylgard 184 (Dow Corning, Midland, MI, USA), DNA primers were synthesized and purified by Iba-lifesciences (Germany) or Metabion (Germany). Bacteriophage lambda DNA (48.5 kb, ThermoFisher Scientific). Ultrapure water (Labostar, Siemens), isopropanol (EMSURE, MERCK), methanol (EMPLURA, MERCK), acetone (Sigma-Aldrich, USA), Tris-acetate (Sigma-Aldrich, USA), KCl (Carl Roth GmbH, Germany). Buffer solutions: **A)** 33 mM Tris-acetate (pH 7.9), 66 mM KCl, **B)** 40 mM Tris-HCl, (pH 8.0) 1 mM MgCl2, 1 mM DTT, **C)** 40 mM Tris-HCl, (pH 8.0), 133 mM KCl, **D)** 0.1 M sodium bicarbonate, (pH 8.0).

Glass surface silanization and PEGylation. First, we cleaned glass coverslips (25 x 25 mm^2, #1.5, Menzel Glaser) surface by washing with 1% (v/v) alconox solution, ultrapure water, and then immersed into 1 M of NaOH solution for 1 hour. After incubation in NaOH we rinsed coverslips with ultrapure water, dried unsing N_2 gas and cleaned by air plasma (5 min, ~500 mTorr, high-power mode, PDC-002, Harrick, USA). Immediately after plasma cleaning we placed coverslips in a staining jar filled with aminosilane (0.6 ml, 8.19172 EMD Millipore) solution in methanol (75 ml) and acetic acid (3 ml) for 5 min. After silanization we rinsed coverslips placed in the jar with methanol, and then with ultrapure water. Next, we dried coverslips with N_2 gas, and performed PEGylation in a sandwich format (two coverslips placed on top of each other) using a 60 µl drop of 10:1 methoxy-PEG-SVA:biotin-PEG-SVA (w/w, both 5 kDa, Lyasan Bio, USA) in buffer D. PEGylation took overnight, and then the coverslips disassembled from sandwich were washed with ultrapure water and stored at -20 °C until use.

TIRF microscopy. The employed home-build TIRF microscopy setup was described previously [1]. This microscopy setup was equipped with three different wavelength lasers: 488 nm, 532 nm and 635 nm (all 20 mW, Crystalaser, USA). These combined beams were directed to the objective (100x, 1.4NA, Nikon) using a quad-line dichroic mirror (zt405/488/532/640rpc, Chroma Technology Corp) installed in the microscope body (Nikon Eclipse Ti-U). The laser power before the objective was set to 2.5 mW for both 532 nm and 632 nm lasers, and to 0.1 mW for the 488 nm laser, respectively. The exposure time of the EMCCD camera (Ixon3, Andor) was set to 100 ms. The penetration depth of the evanescent field was set to ~300 nm for all wavelengths of excitation. This setup was equipped with a custom-build feed-back control system to compensate the Z-axis drift of the sample and keep it stably in focus.

Protein nanopatterning by lift-off µCP and the Soft DNA Curtains. Biotin (bt) and digoxigenin (dig) functionalized λ DNA (bt-λ DNA-dig) was prepared and purified in the same way as previously [2]. Si master with 0.5 µm line-width and 13 µm line-spacing was fabricated according to the previously published procedure [1, 2]. Flat ~2 mm thickness PDMS (PDMS elastomer) elastomer (10:1 ratio w/w, Sylgard 184 kit) stamps for protein lift-off µCP were fabricated according to the previously published procedure [1].

The lift-off µCP was performed similarly to the published procedure [2]. The Si master was sonicated in acetone for 15 min and then 15 min in isopropanol, dried under N_2 gas, and cleaned by air plasma (5 min, ~500 mTorr, high-power mode, PDC-002, Harrick, USA). The PDMS elastomer (5 x 5 mm^2) was immersed in isopropanol for 10 min, held by tweezers and dried for 15 s using N_2 gas, placed on a clean Petri dish and dried under air for another 10 min. Next, the stamp was placed facing flat side-up on a piece of glass (10 x 10 mm^2), which was covered with the double-sided sticky tape (9088-200,

3M). To homogeneously cover the PDMS surface with a film of the protein ink a 60 µl drop of 0.17 mg/ml traptavidin (tAv) protein solution (in buffer A) was placed on the stamp surface, mixed with the tip of pipette, and kept for 10 min. After incubation the protein ink was removed from the stamp by sucking it out with the pipette tip. Then, the stamp on glass slide was held with tweezers and washed with 5 ml of buffer A using a 1 ml pipette, ~50 ml ultrapure water using a wash bottle, and dried under N_2 gas stream. For the printing procedure we employed a semi-automated printing machine [2]. First, the cleaned Si master was placed on the silicon rubber on the bottom of the printing machine and the pressure of 0.6 ml for both printing steps on Si master and silanized/PEGylated glass coverslip. The patterned glass coverslip was assembled into the flowcell, which was prepared as described earlier [1].

To immobilize the bt-λ DNA-dig the channel of the flowcell was filled with buffer B. To enhance surface passivation against non-specific protein adsorption, we injected 5% (v/v) Tween-20 solution in buffer B into the channel of the flowcell, incubated for 10 min and washed out with 600 µl of buffer A. Next, the DNA (~40 pM 150 µl, in buffer B) was injected and incubated for at least 15 min. The excess of unbound DNA was washed out with ~300 µl of buffer A. Then DNA was labelled with the DNA intercalating green fluorescent dye – SYTOX green (SG, ThermoFisher Scientific, USA) at a concentration of ~0.4 nM (in buffer C). The SG dye was present during the entire time of the experiment. For the second DNA end tethering, the close-loop circulation was employed and 5 µl of biotin-anti-dig (bt-anti-dig) antibody was added (this resulted in ~0.05 mg/ml concentration) and incubated for at least 10 min at low speed (~0.1 ml/min). After 10 min, the speed of the buffer flow was increased to ~1 ml/min and kept constant for 20 min. Then, to remove the excess of unbound bt-anti-dig the flowcell was washed with 500 µl of buffer A in the open-loop circulation. Finally, 100 µl of imaging buffer was injected into the flowcell in order to reveal bound DNA.

Expression and purification of traptavidin and monovalent streptavidin. We followed previously published protocols for traptavidin (tAv) and monovalent streptavidin (mSav) preparation [3]. *E. coli* BL21 (DE3) strain cells were transformed with pET21a tAv or pET21a streptavidin (sAv) (Alive or Dead) plasmids, plated onto Luria-Broth (LB)-Carbenicillin agar plates and incubated at 37 °C overnight. An overnight culture, which was prepared by inoculating a colony from the agar plate in LB- Ampicillin (Amp) medium and shaking at 220 r.p.m. and 37 °C overnight, was then diluted 100-fold into LB-Amp, grown at 37 °C until OD600 0.9, induced with 0.5 mM isopropyl-β-D- thiogalactopyranoside and shaken further at 37 °C for 4 h. After spinning the bacteria at 5000*g* and 4 °C for 10 min, the cell pellet was resuspended in a lysis buffer (50 mM Tris-HCl (pH 7.8), 300 mM NaCl, 5 mM EDTA, 0.8 mg/ml lysozyme, 1% (v/v) Triton X-100) and then put on a rocker at 80 r.p.m. and RT for 20 min. Pulsed sonication of the cell pellet on ice at 30% amplitude was performed afterwards for 10 min. Centrifugation at 27000 *g* and 4 °C for 15 min was followed by three-times washing of the inclusion body pellet in a wash buffer (50 mM Tris-HCl (pH 7.8), 100 mM NaCl, 0.5% (v/v) Triton X-100). Isolated inclusion bodies were dissolved in 6 M guanidinium hydrochloride (GuHCl) (pH 1.5) and then spun at 17700 *g* and 4 °C for 20 min. Solubilized Alive and Dead sAv subunits were accordingly mixed to obtain a 5-fold molar excess of Dead sAv subunit in respect to Alive subunit. The resultant mixture of two different sAv subunits and GuHCl-dissolved tAv monomers were separately refolded by diluting them rapidly into PBS at 4 °C and stirring overnight at the same temperature. Protein precipitation using solid ammonium sulfate was then carried out in order to precipitate sAv and tAv from their refolds. The obtained precipitates were resuspended in a minimum volume of PBS at room temperature, centrifuged

at 14000 *g* and 4 °C for 5 min and the excess of ammonium sulfate was removed by running the supernatant through a NAP-25 column. GE AKTA Prime Plus liquid chromatography system was used to purify tAv and monovalent sAv (mSav). Mixture of distinct sAv tetramers was loaded on the equilibrated HiTrap Chelating HP 5 ml column and mSav was then eluted by applying gradient elution. An elution buffer (50 mM Tris-HCl (pH 7.8), 300 mM NaCl, 0.5 M imidazole) along with a start buffer (50 mM Tris-HCl (pH 7.8), 300 mM NaCl) was used during this procedure. Same steps were followed in the case of affinity-based tAv purification. After pooling the fractions containing tAv or mSav, the resultant protein solution was dialyzed one time in PBS at 4 °C overnight. Purified proteins were concentrated by using a 9 kDa MWCO centrifugal concentrator and spinning at 4800*g* and 4 °C for 20-30 min. The obtained final yields of tAv and mSav were ~ 2 mg/l and ~ 3 mg/l of initial culture, respectively.

We labelled the mSav using NHS-ATTO647N ester. We mixed 10 μM of mSav with 20 μM of NHS-ATTO647N (dissolved in DMSO (dimethyl sulfoxide)) in PBS and incubated at RT for 1 hour. After incubation, we quenched the excess dye using glycine. Then, we purified the protein using NAP-5 column (GE Healthcare). The purified protein had ~1 μM concentration.

The volume analysis of DNA-protein complexes. Atomic force microscopy produce three-dimensional topological images. The heights and diameters of a number of particles were determined and used to calculate molecular volumes (Eq. 1). Particle diameter was measured at half the maximal height.

$$V_{AFM} = \frac{\pi h}{6}\left(3 * \frac{d^2}{4} + h^2\right) \qquad \text{(Eq. 1)}$$

Where h is the height of the protein particle, d is its diameter. The theoretical volume is determined by Eq. 2:

$$V_{calculated} = \frac{M_W}{N_A}(V_1 + dV_2) \qquad \text{(Eq. 2)}$$

Here, M_W is the molecular weight, N_A is Avogadro's number, and V1 and V2 are the partial specific volumes of the individual protein 0.74 cm^3 g^{-1} and 1 cm^3 g^{-1} water, respectively. d is the extent of protein hydration (0.4 mol H2O/mol protein) [4].

Mean values for particle molecular volume are given in Supplementary Table S4.

Supplementary Figures

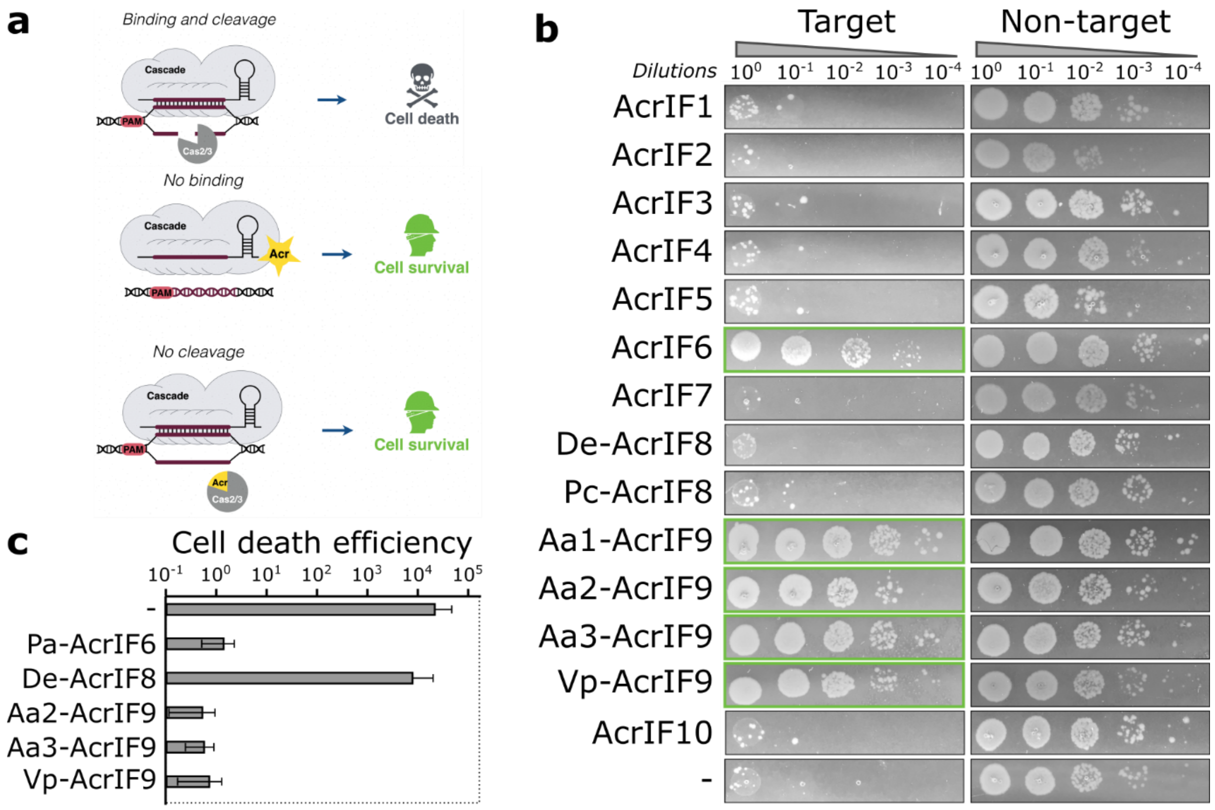

Supplementary Figure S1. *Inhibition of the Aa-CRISPR-Cas system by AcrIF proteins.* (**a**) Principles of the survival assay. Cascade complex containing *E. coli* genome targeting guide specifically binds genomic DNA triggering its hydrolysis by Cas2/3 what leads to cell death. The AcrIF protein may block either Cascade or Cas2/3 resulting in cell survival. (**b**) Representative LB-agar plating results. *E. coli* cells carrying Cas proteins and respective AcrIF protein were transformed with a vector bearing either targeting or non-targeting CRISPR locus. Ten-fold dilutions of the transformants were plated on LB-agar. Empty vector (-) was used as a control. The plates with rescued cells are delineated by green rectangles. (**c**) Cell death efficiency in the presence of indicated AcrIF proteins (not shown in the main text). The ratio of transformation efficiencies of non-targeting and targeting guides was expressed as cell death efficiencies. Error bars represent standard deviations of average in at least three separate experiments.

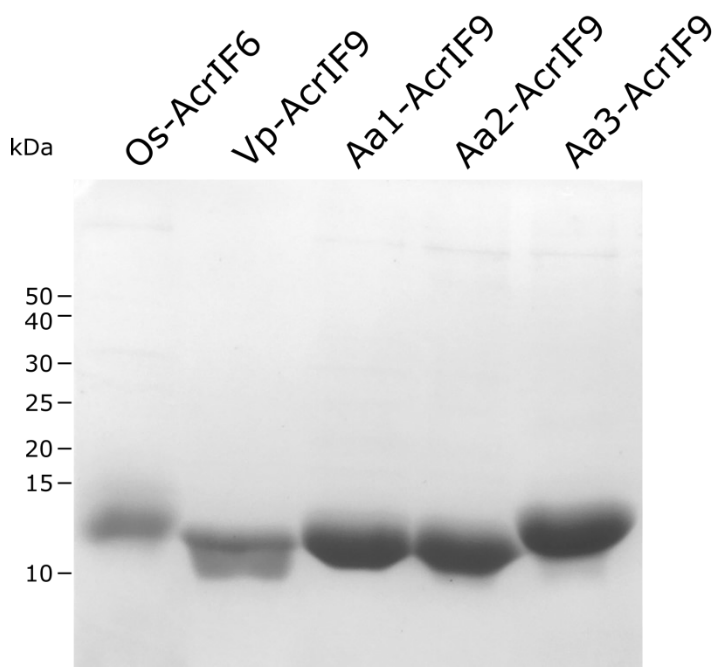

Supplementary Figure S2. *SDS-PAGE of purified AcrIF6 and AcrIF9 proteins.* Purified samples of respective AcrIF6 and AcrIF9proteins were fractionated in 15% polyacrylamide gel under denaturing conditions.

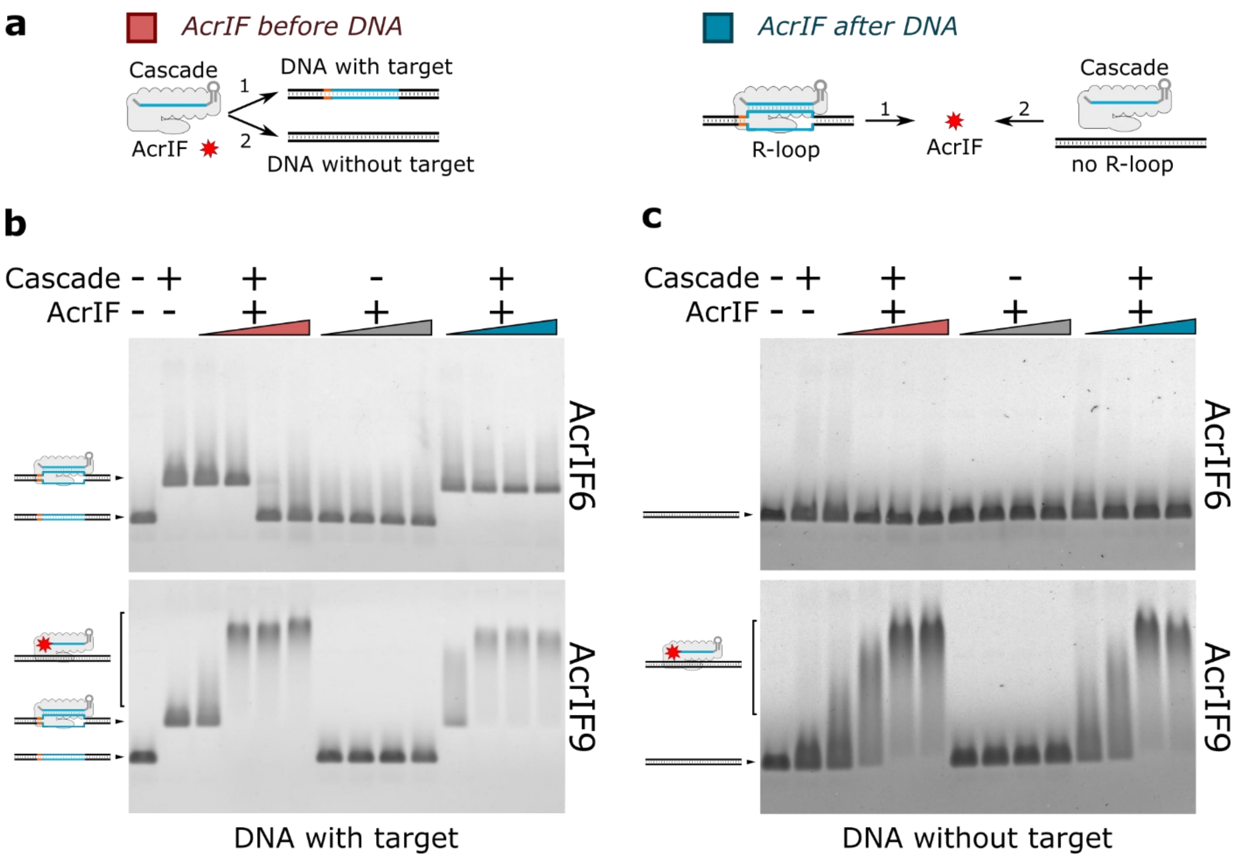

Supplementary Figure S3. *Influence of AcrIF6 and AcrIF9 for Cascade binding to target and non-target DNA.* (**a**) Mixing order of reaction components. AcrIF6 or AcrIF9 protein was introduced either before (red-coded) or after (blue-coded) DNA addition. The Cascade binds to DNA containing target sequence (1) resulting in the R-loop formation, while it should not bind DNA without target sequence (2). EMSA of (**b**) target and (**c**) non-target in the presence of Cascade and AcrIF6/9. Increasing AcrIF concentrations (30, 300, 3000, 20000 nM) were introduced to 100 nM Cascade binding reactions as indicated in (A). DNA binding reactions mediating only AcrIF6/9 proteins (without Cascade) are indicated by a grey triangle.

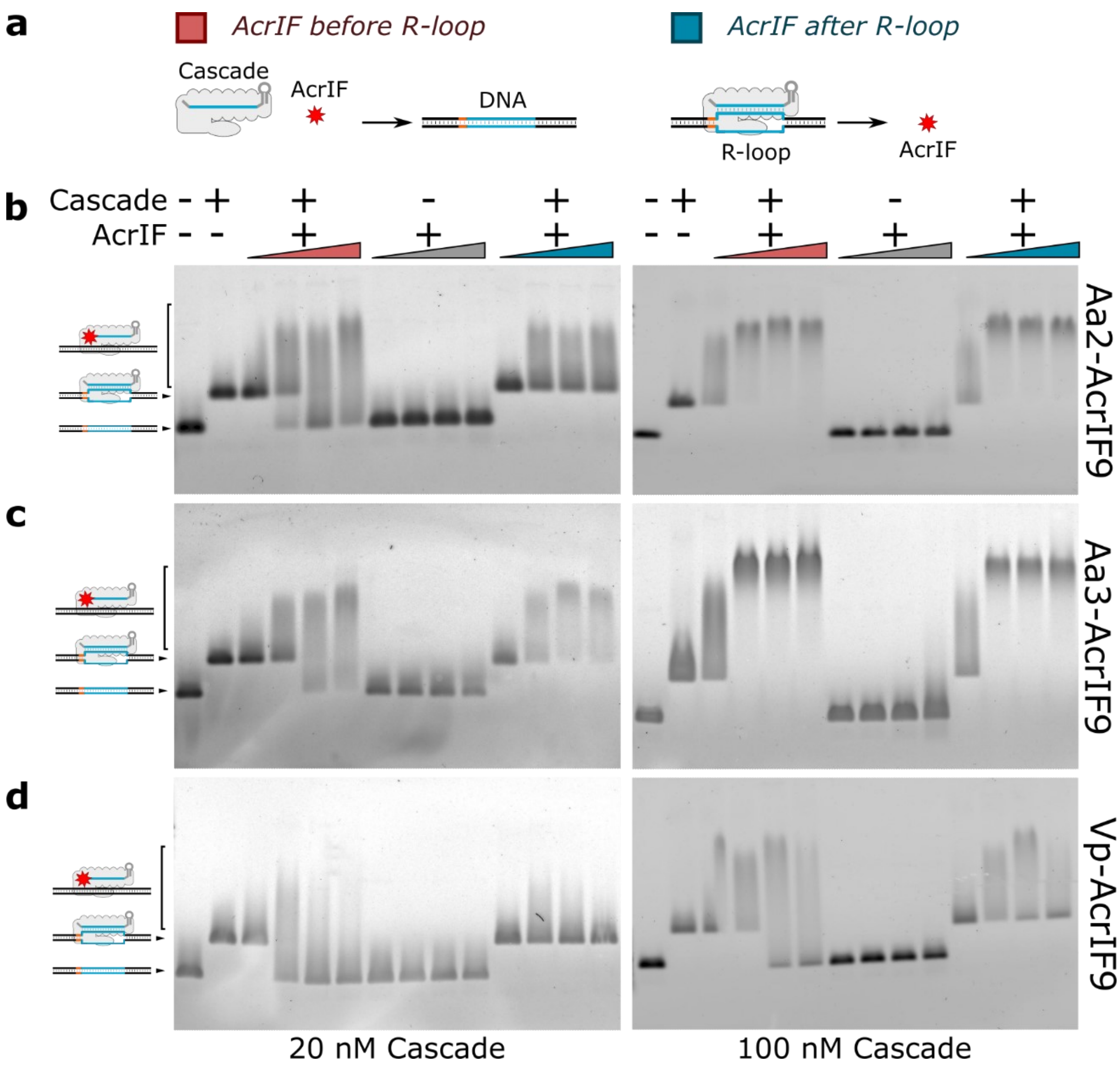

Supplementary Figure S4. *AcrIF9 homologs block Cascade binding to DNA target and stimulate non-specific DNA binding*. (**a**) Mixing order of reaction components. AcrIF9 protein was introduced either before (red-coded) or after (blue-coded) the R-loop formation. Cascade binding to DNA target in the presence of Aa2- (**b**), Aa3- (**c**), and Vp-AcrIF9 (**d**) homologues. Increasing AcrIF9 concentrations (30, 300, 3000, 20000 nM) were introduced to either 20 nM (left-side panels) or 100 nM (right-side panels) Cascade binding reactions as indicated in (**a**). DNA binding reactions mediating only AcrIF6/9 proteins (without Cascade) are indicated by a grey triangle. The binding reactions were assayed by EMSA in agarose gel.

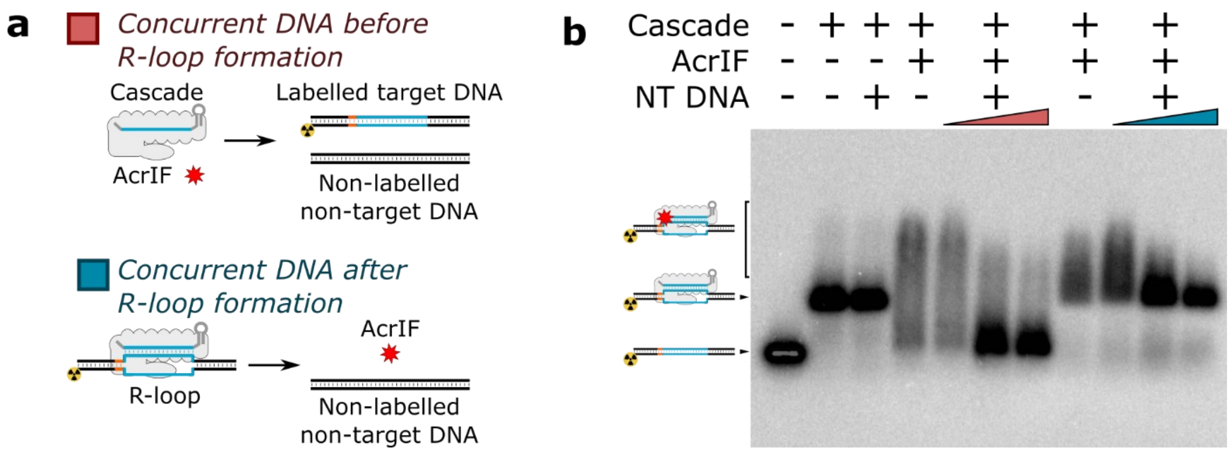

Supplementary Figure S5. *The AcrIF9-mediated Cascade binding to target DNA in the presence of non-specific DNA*. (**a**) Mixing order of reaction components. Two approaches were used for concurrent (non-target) DNA addition: (i) AcrIF9 protein was incubated with Cascade then added to a mixture of the labelled target DNA and non-labelled concurrent DNA containing no target sequence (red-coded); (ii) the preformed R-loop was introduced to the mixture of AcrIF9 and non-labelled concurrent DNA (blue-coded). (**b**) EMSA of binding reactions. Increasing concentrations of concurrent ~48 kbp length λ phage genomic DNA (0.05, 0.5, and 5 nM) were introduced to binding reactions of 20 μM AcrIF9,100 nM Cascade and 20 nM labelled target DNA (~0.3 kbp length) as indicated in (**a**).

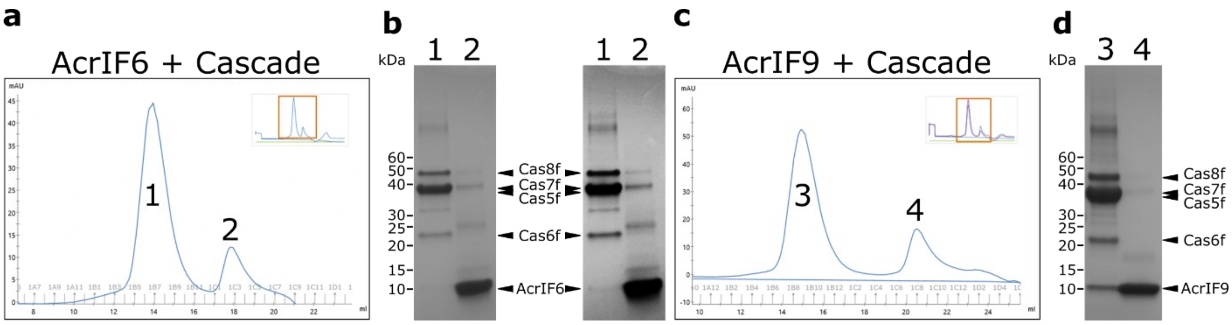

Supplementary Figure S6. *Cascade interacts with AcrIF6 and AcrIF9*. Cascade was incubated with AcrIF6 or AcrIF9 and fractionated using Superdex 200 size exclusion chromatography column. Elution peaks 1 and 2 depicted in the chromatogram of AcrIF6 and Cascade incubation (**a**) was analyzed by SDS-PAGE (**b**). The gel in the right panel of (**b**) represents the gel of the left panel with modified brightness and contrast to sharpen the poorly stained band of AcrIF6, which eluted together with Cascade (1). Elution peaks 3 and 4 depicted in the chromatogram of AcrIF9 and Cascade incubation (**c**) was analyzed by SDS-PAGE (**d**). Expected molecular weights for Cas8f, Cas5f, Cas7f, Cas6f, AcrIF6 and AcrIF9 proteins were approximately 52, 36, 38, 23, 11.6, and 10,3 kDa, respectively.

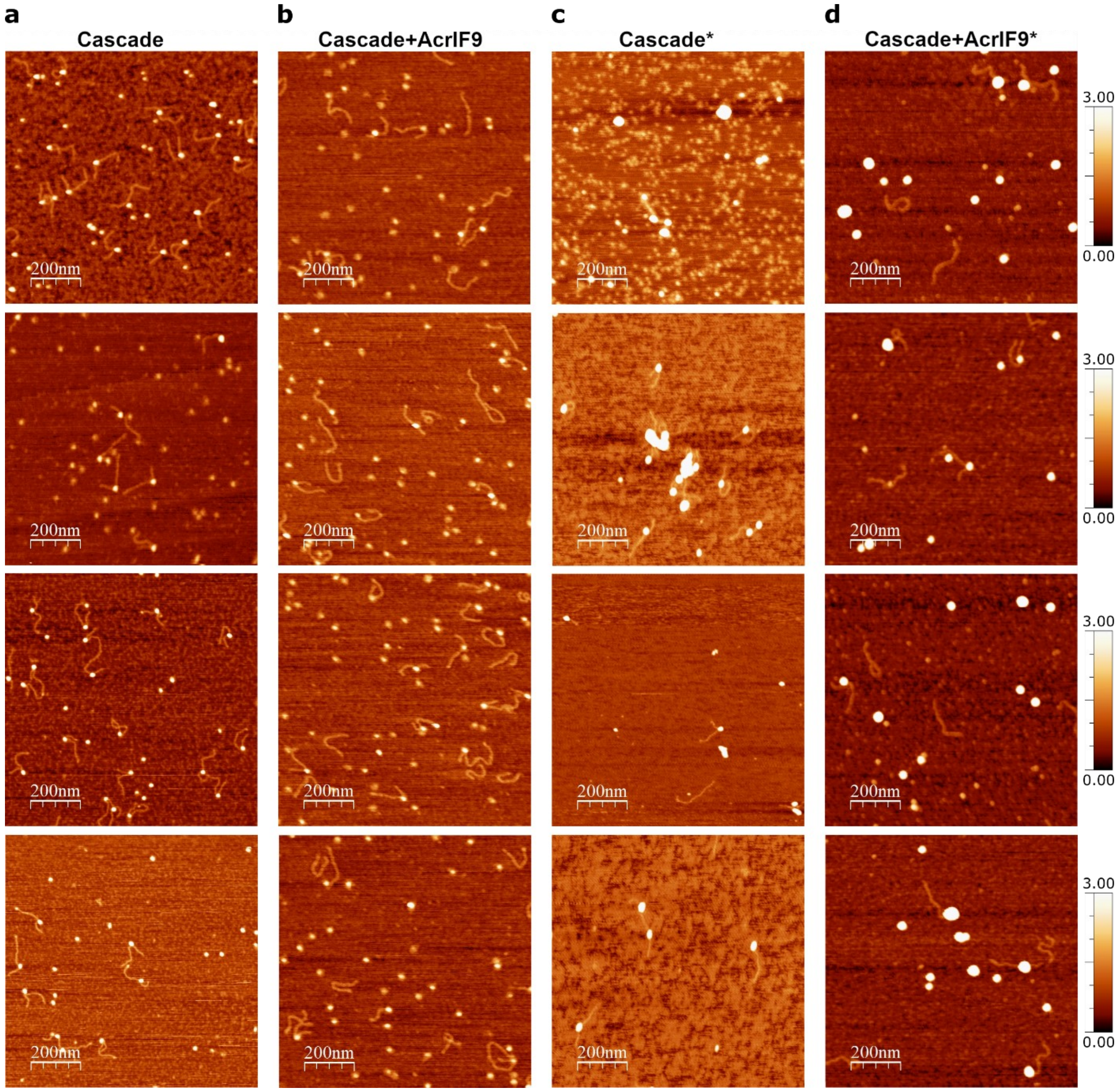

Supplementary Figure S7. *AFM images of studied Cascade and DNA complexes*. **(a)** Cascade and **(b)** AcrIF9-Cascade binding to DNA in the absence of the cross-linker. **(c)** Cascade and **(d)** AcrIF9-Cascade binding to DNA in the presence of the cross-linker (2% (v/v) of glutaraldehyde; indicated by asterisk).Scan size is 1 μm by 1 μm. Z range is 3 nm. The differences in the background morphology show uneven distribution of APS layer.

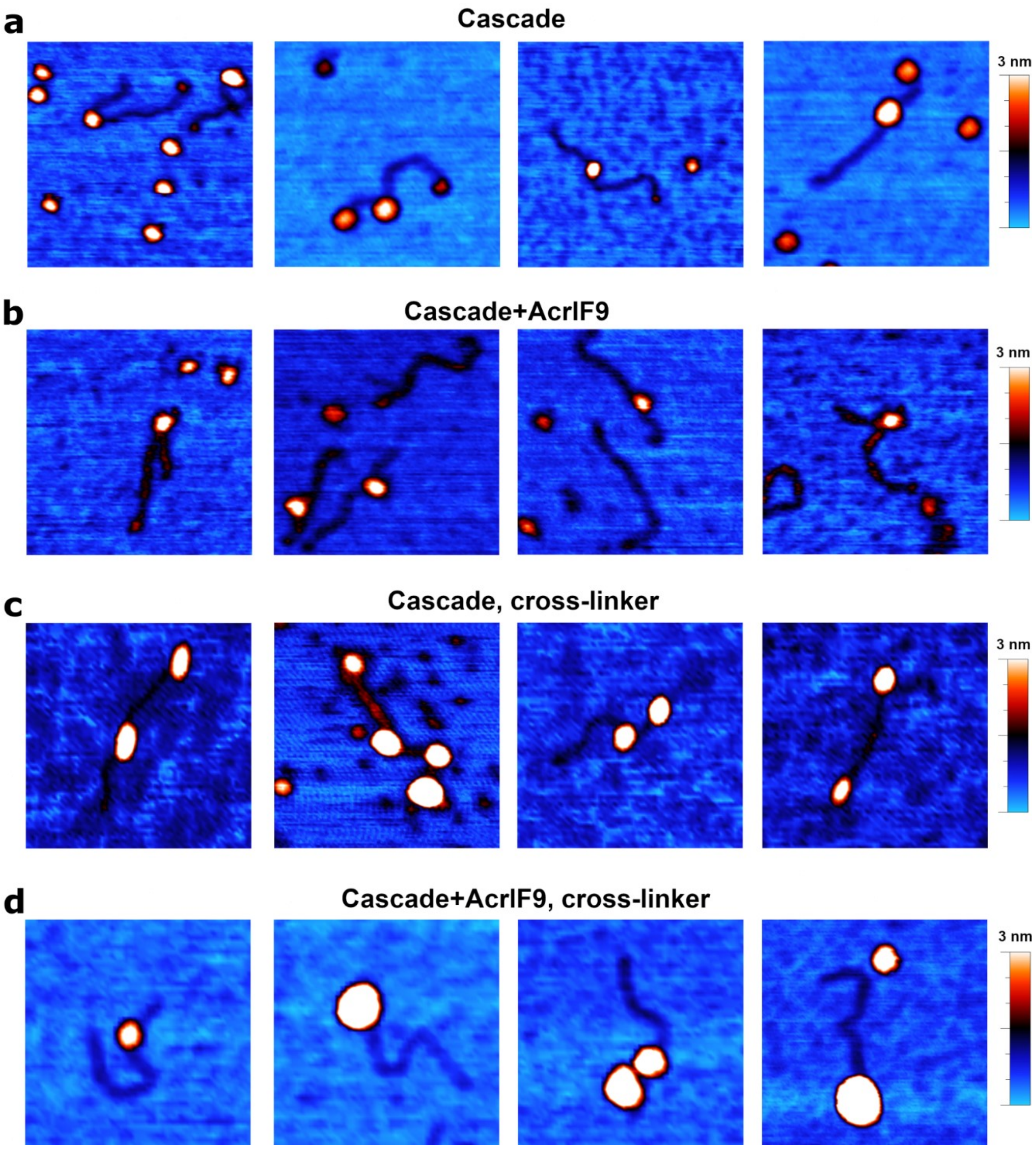

Supplementary Figure S8. *AFM images show different types of single Cascade and DNA complexes.* (**a**) Cascade and (**b**) AcrIF9-Cascade binding to DNA in the absence of the cross-linker. (**c**) Cascade and (**d**) AcrIF9-Cascade binding to DNA in the presence of the cross-linker (2% (v/v) glutaraldehyde). The height scale is 3 nm. Scan size 250 nm by 250 nm.

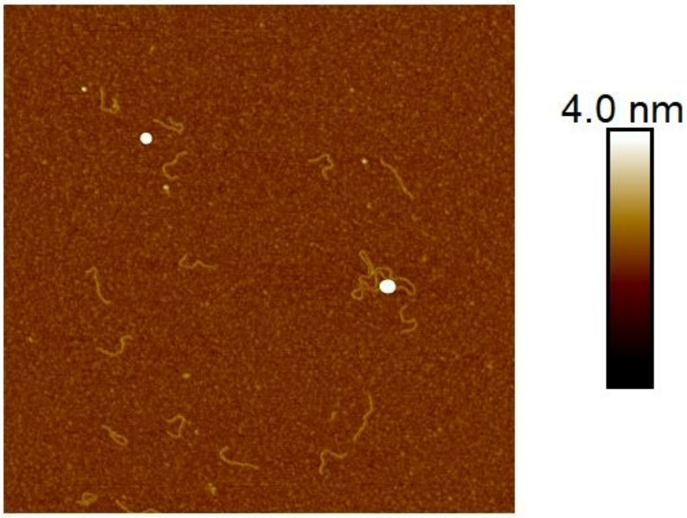

Supplementary Figure S9. *AFM image of AcrIF9 incubation with DNA*. AcrIF9:DNA incubated in 20:1 ratio in the presence of 2 % of glutaraldehyde and adsorbed on APS-mica. Scan size 2 μm by 2 μm.

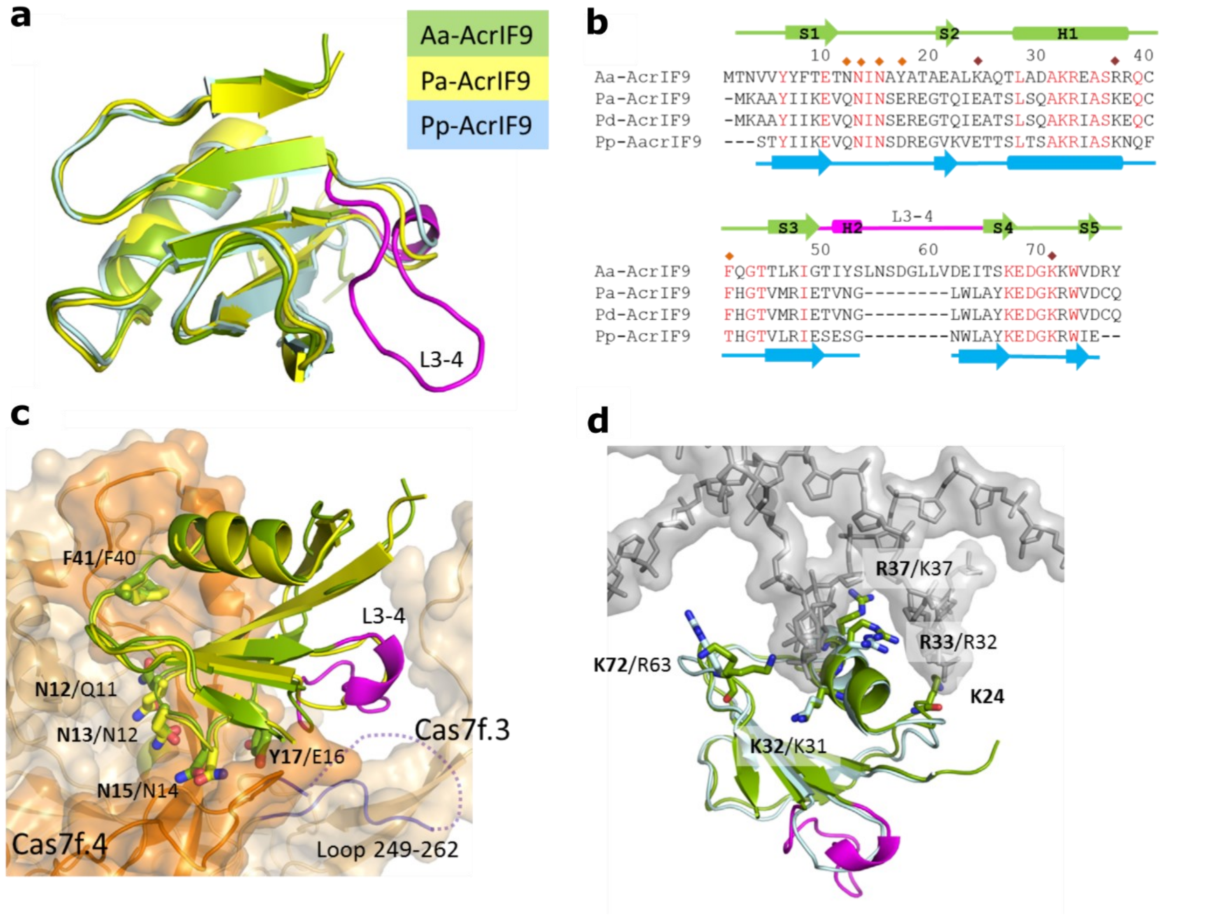

Supplementary Figure S10. *Aa1-AcrIF9 structural superimpositions*. (**a**) Superposition of Aa1-AcrIF9 (green) with Pa-AcrIF9 (PDB ID 6VQV, chain A, yellow) and Pp-AcrIF9 (PDB ID 6W1X, chain J, light blue). (**b**) Structure-based sequence alignment of AcrIF9 homologs. Identical residues are red. Aa1-AcrIF9 residues presumably involved in contacts with Cas7f and DNA are marked by orange and purple diamonds, respectively. The loop L3-4 is coloured magenta. (**c**) Model of Aa1-AcrIF9 binding to the Pa-Cascade complex. Aa1-AcrIF9 (green) was superimposed with the chain A of the Pa-AcrIF9-Cascade complex (6VQV). Cas7f.4 and Cas7f.3 subunits are coloured orange and light orange, respectively. Residues of Aa1-AcrIF9 interacting with Cas7f.4 are shown in stick representation and labelled. Aa1-AcrIF9 residues are labelled in bold. Loop 249-262 of Cas7.3 presumably interacting with L3-4 is coloured blue. (**d**) Model of DNA binding by Aa1-AcrIF9. Aa1-AcrIF (green) was superimposed with the chain J of the Pp-AcrIF9-Cascade-DNA complex (6WHI). Residues of Aa1-AcrIF9 interacting with DNA are shown in stick representation and labelled. Aa1-AcrIF9 residues are labelled in bold.

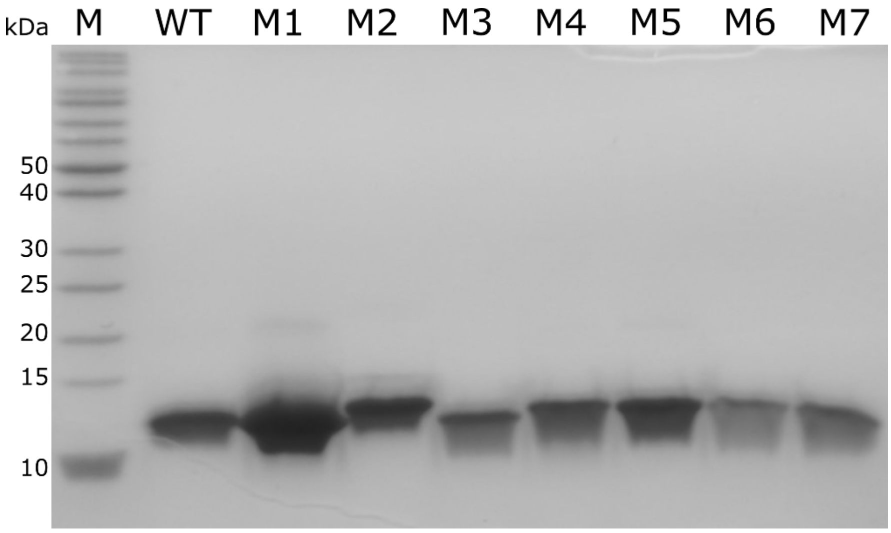

Supplementary Figure S11. *SDS-PAGE of the Aa1-AcrIF9 mutants*. The purified samples of Aa1-AcrIF9 (WT) and its K24A/R37A/K72A (M1), K24E (M2), R37E (M3), N12A/N13A/N15A/Y17A (M4), F41A (M5), ΔL54-V60 (M6), and I51-D61->VNGL (M7) mutants were fractionated in 15% polyacrylamide gel under denaturing conditions.

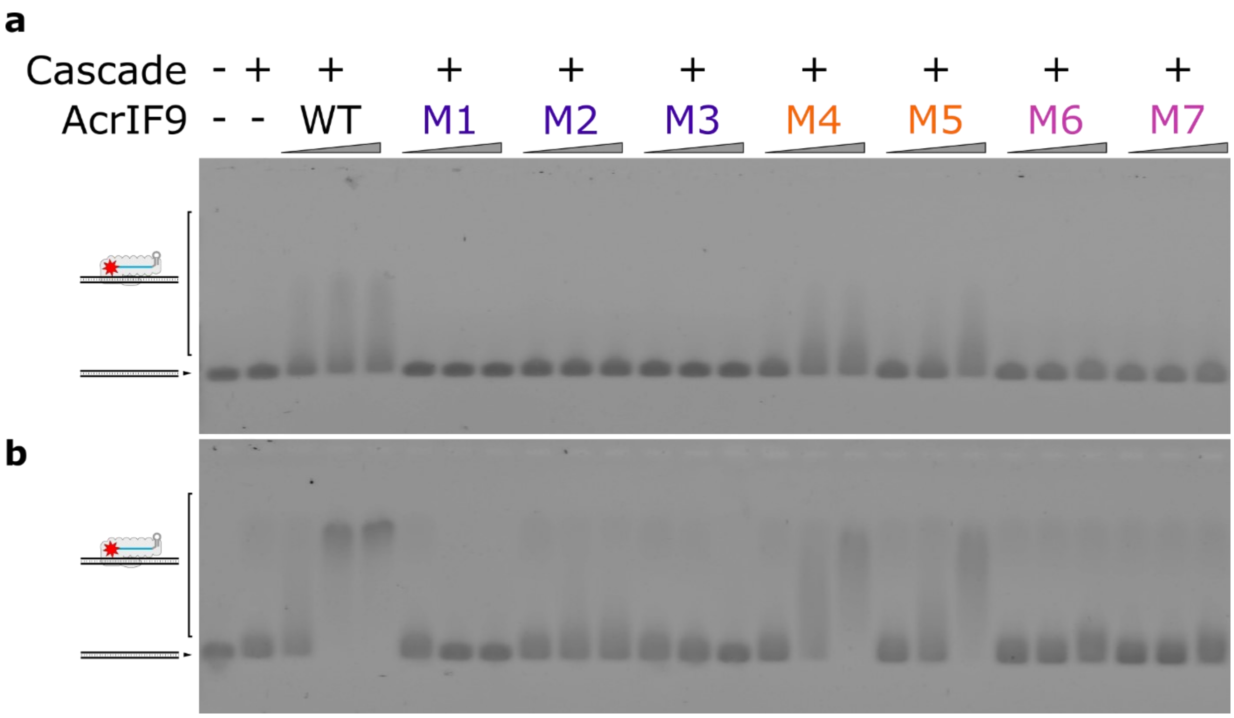

Supplementary Figure S12. *Cascade binding to the non-target DNA in the presence of the AcrIF9 mutants*. The increasing concentrations of the AcrIF9 mutant proteins (50, 500, 5000 nM) incubated with either 20 nM **(a)** or 100 nM **(b)** Cascade then introduced to the binding buffer containing 20 nM non-target DNA (NS). Protein interactions with the DNA were assayed by EMSA in agarose gel. Mutants of Aa1-AcrIF9 (WT) used in this experiment are K24A/R37A/K72A (M1), K24E (M2), R37E (M3), N12A/N13A/N15A/Y17A (M4), F41A (M5), ΔL54-V60 (M6), and I51-D61->VNGL (M7).

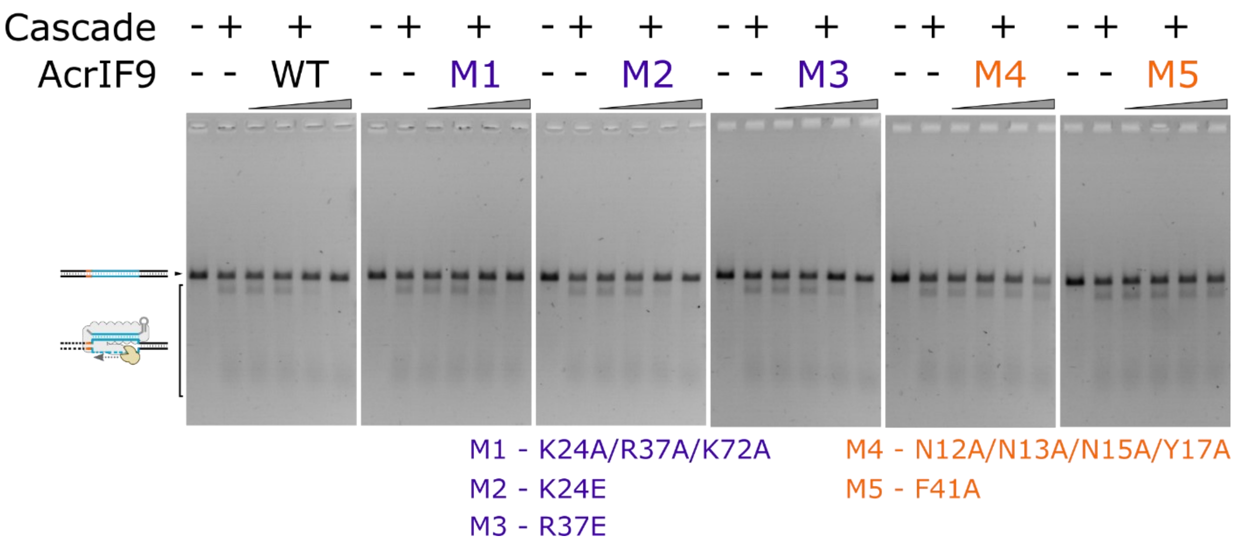

Supplementary Figure S13. *A Cas2/3-mediated cleavage inhibition by AcrIF9 mutants*. Increasing concentrations (5, 50, 500, 5000 nM) of respective Aa1-AcrIF9 mutant were pre-incubated with 20 nM Cascade then mixed with 5 nM target DNA (SP3). The DNA degradation was initiated by the addition of 200 nM Cas2/3. Positions of substrate and degradation products are indicated on the left of the agarose gel. Coding of mutations is provided below the gel. Colour-coding as in Figure 5.

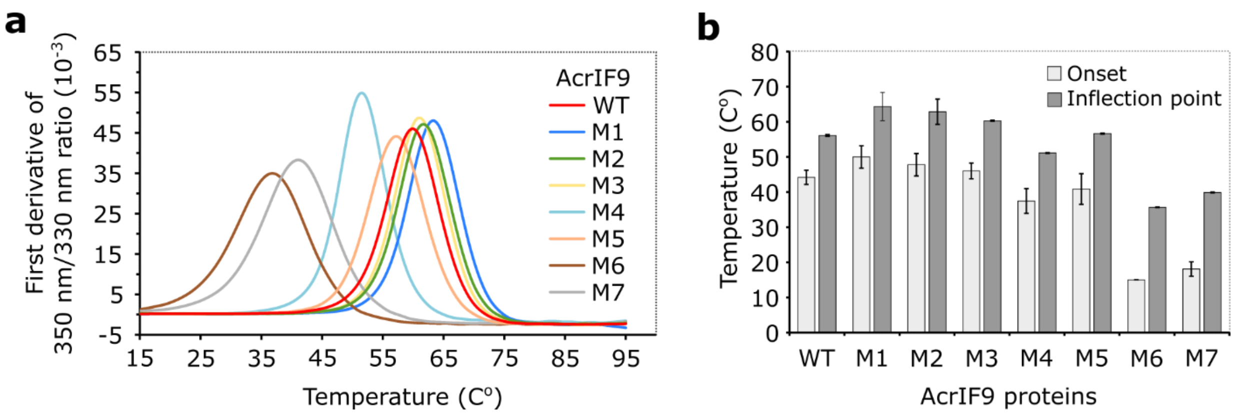

Supplementary Figure S14. *The stability of the Aa1-AcrIF9 proteins*. The samples of Aa1-AcrIF9 (WT) and its K24A/R37A/K72A (M1), K24E (M2), R37E (M3), N12A/N13A/N15A/Y17A (M4), F41A (M5), ΔL54-V60 (M6), and I51-D61->VNGL (M7) mutants were assayed by nanoscale differential scanning fluorimetry. (**a**) Denaturation curves are provided as the first derivative of the 350 nm / 330 nm ratio. (**b**) Temperatures of the denaturation curve onset and inflection points. Error bars represent standard deviations of average in at least three separate measurements.

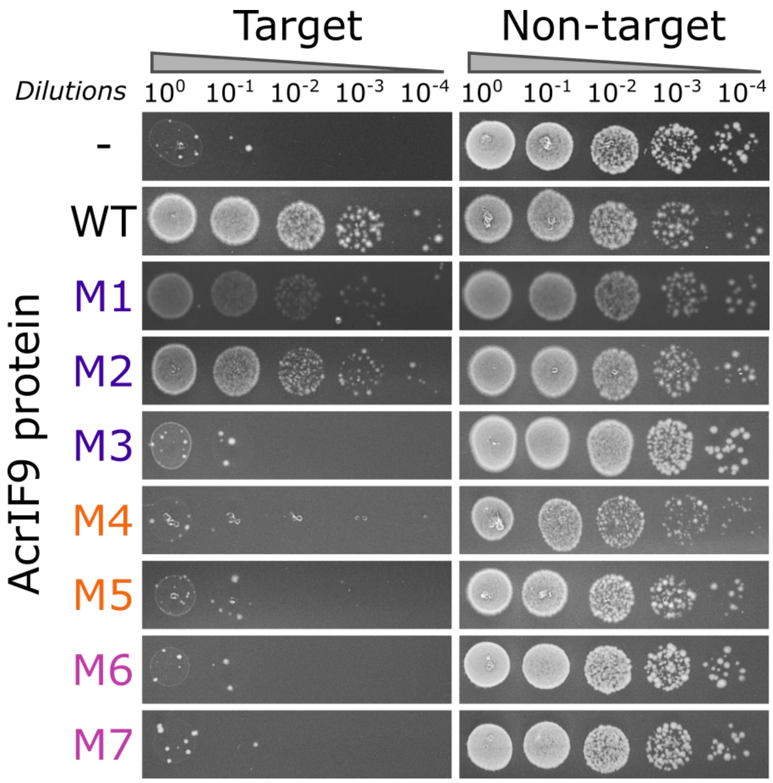

Supplementary Figure S15. *Representative plating results of Aa-CRISPR-Cas inhibition by AcrIF9 mutant proteins. E. coli* cells carrying Cas proteins and respective AcrIF9 mutant were transformed with a vector bearing either targeting or non-targeting CRISPR locus. Ten-fold dilutions of the transformants were plated on LB-agar. Empty vector (-) was used as a control. Mutants of Aa1-AcrIF9 (WT) used in this experiment are K24A/R37A/K72A (M1), K24E (M2), R37E (M3), N12A/N13A/N15A/Y17A (M4), F41A (M5), ΔL54-V60 (M6), and I51-D61->VNGL (M7).

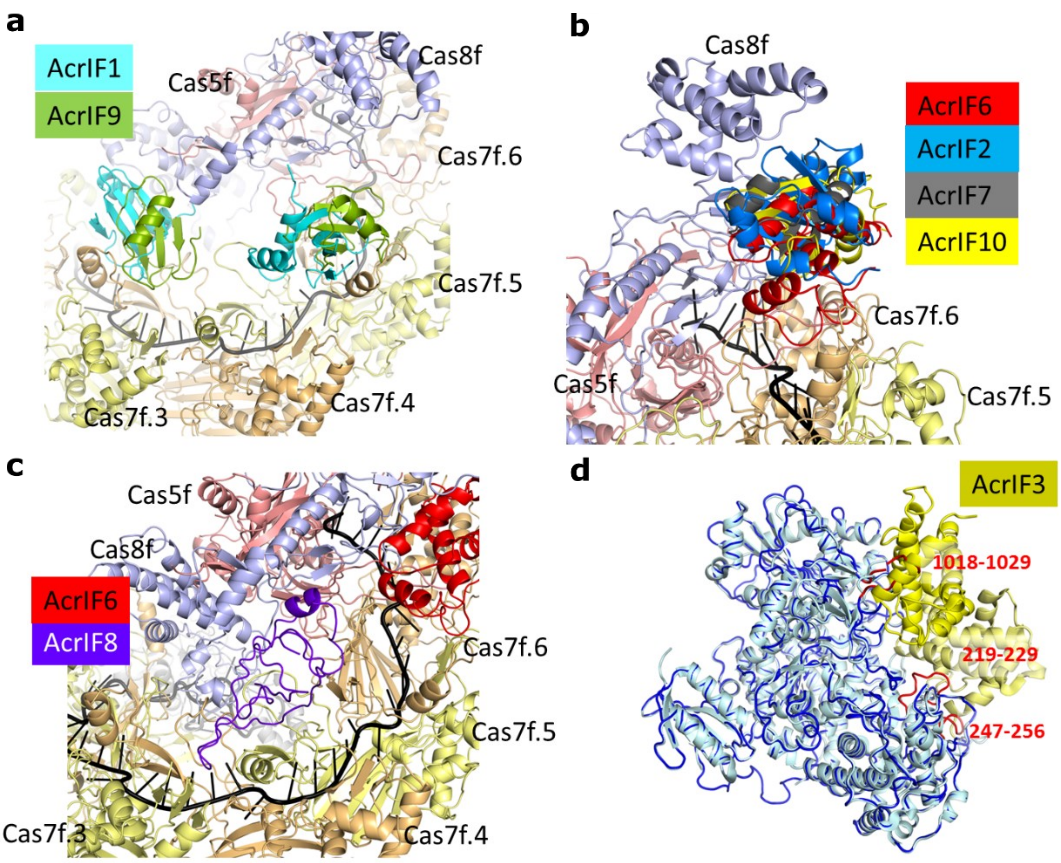

Supplementary Figure S16. *Comparison of AcrIFs bound to Pa-CRISPR-Cas system.* (**a**) Binding of AcrIF1 (PDB ID 5UZ9) and AcrIF9 (6WHI). (**b**) Binding of AcrIF2 (PDB ID 5UZ9), AcrIF6 (6VQX), AcrIF7 (7JZX) and AcrIF10 (6B48). (**c**) Binding of AcrIF8 (6VQW), AcrIF6 is shown for comparison. (**a-c**) corresponding Cascade-AcrIF structures were superimposed via Cas8f subunit. (**d**) Model of Aa-Cas2/3 interaction with AcrIF3. Homology model of Aa-Cas2/3 (dark blue) was generated using SWISS MODEL server, template Pa-Cas2/3 (PDB ID 5B7I) and superimposed with Pa-Cas2/3 (light blue)-AcrIF3 dimer (yellow) structure (5B7I). Unique Aa-Cas2/3 structural elements on the interaction interface are coloured red.

Supplemenary Table S1. Percent identities of pairwise alignment between type I-F CRISPR-Cas from *A. actinomycetemcomitans* proteins and *P. aeruginosa* and *P. atrosepticum* CRISPR-Cas systems that were used to assay AcrIF proteins.

A. actinomycetemcomitans	**Cas1**	**Cas2/3**	**Cas8f**	**Cas5f**	**Cas7f**	**Cas6f**
P. aeruginosa	60%	45%	27%	33%	53%	29%
P. atrosepticum	58%	41%	30%	33%	54%	33%

Supplementary Table S2. AcrIF proteins and their interaction with CRISPR-Cas systems

**Family**	**Named in this study**#	**Homologue species**	**Accession**	**CRISPR-Cas system***			**Interacts with Cas protein**%
				P. aeruginosa$	***P. atrosepticum***$	***A. actinomycetemcomitans***	
AcrIF1	AcrIF1	Pseudomonas aeruginosa	WP_016068276.1	+	±	-	Cas7f.3-4, Cas7f.5-6, Cas8f
AcrIF2	AcrIF2	Pseudomonas aeruginosa	WP_015972868.1	+	+	-	Cas8f
AcrIF3	AcrIF3	Pseudomonas aeruginosa	WP_016068567.1	+	-	-	Cas2/3
AcrIF4	AcrIF4	Pseudomonas aeruginosa	WP_016068584.1	+	-	-	Cas8f, Cas5f
AcrIF5	AcrIF5	Pseudomonas aeruginosa	WP_016068568.1	+	-	-	
AcrIF6	AcrIF6 or Os-AcrIF6	Oceanimonas smirnovii	WP_019933870	+	+	+	
	Pa-AcrIF6	Pseudomonas aeruginosa	WP_043884810	+	+	+	Cas8f, Cas7f.6, Cas5f
AcrIF7	AcrIF7	Pseudomonas aeruginosa	ACD38920.1	+	+	-	Cas8f
AcrIF8	Pp-AcrIF8	Pectobacterium phage ZF40	AFC22483.1	+	+	-	Cas5f, Cas7f.4-6, Cas8f
	Ds-AcrIF8	Delftia sp. 670	KEH13790.1	+	+	-	
AcrIF9	Vp-AcrIF9	Vibrio parahaemolyticus	WP_031500045.1	+	+	+	Cas7f.4, Cas7f.6
	AcrIF9 or Aa1-AcrIF9	Aggregatibacter actinomycetemcomitans serotype e str. SC1083	WP_005556152.1	ND	ND	+	
	Aa2-AcrIF9	Aggregatibacter actinomycetemcomitans serotype b str. SCC1398	WP_005567629.1	ND	ND	+	
	Aa3-AcrIF9	Aggregatibacter actinomycetemcomitans D11S-1	WP_005544627.1	ND	ND	+	
AcrIF10	AcrIF10	Shewanella xiamenensis	KEK29119	+	+	-	Cas8f

#Sequences of genes used in this study is provided in the Supplementary Table S6.
*AcrIF *in vivo* activity against the respective CRISPR-Cas system: active (+), partially active (±), and inactive (-).
$Provided in respective studies [5, 6].
% Provided in respective studies [7-13]

Supplementary Table S3. The distribution of protein complex binding positions to DNA. Values represent the mean and S.D., *n* indicates the number of measured bound proteins.

Complex (molar ratio)	Cross-linker	Position from DNA end		Non-target:target molecule ratio
		Non-target site	Target-site	
Cascade-DNA (1:1)	*no*	10.5±7.7 % (n=14)	36.3±3.7 % (n=52)	**1:4**
AcrIF9-Cascade-DNA (20:1:1)	*no*	5.1±0.6 % (n=3)	34.5±4.7 % (n=26)	**1:9**
Cascade-DNA (1:1)	*yes*	10.0±4.1 % (n=18)	37.8±5.6 % (n=33)	**1:2**
AcrIF9-Cascade-DNA (20:1:1)	*yes*	9.1±3.9 % (n=14)	37.9±5.8 % (n=16)	**1:1**

Supplementary Table S4. Volume analysis of protein complexes bound to DNA in AFM images. The values represent mean±S.E.M.

Complex (M_W)	Cross-linker	Measured volume#, nm^3	Theoretical volume$, nm^3	n
Cascade-DNA (357 kDa)	*no*	585.3±8.3	676	33
Cascade-AcrIF9-DNA (377 kDa)	*no*	576.0±8.2	695	29
Cascade-DNA (357 kDa)	*yes*	1157.6±120.8	676	43
Cascade* (357 kDa)	*yes*	1149.7±161.7	676	15
Cascade-AcrIF9-DNA (377 kDa)	*yes*	1546.4±52.2	695	30

# - AFM measured molecular volume was calculated using Eq. 1; $ - theoretical molecular volume was calculated using Eq. 2. For more details see „The volume analysis of DNA-protein complexes“ in Supplementary methods. * - Cascade complexes bound to the surface without DNA.

Supplementary Table S5. Plasmids used in this study.

Plasmid	Description	Cloning sites	Primers	Source
**Cloning**				
pCDF-HS [Str]	T7 promoter based expression vector. AcrIF coning. *In vivo* experiment empty control.			[14]
pCOLA-Duet1[Kn]	T7 promoter based expression vector			Novagen (EMD Millipore)
pCR-cloning [Cm]	Repeats cloned into pACYC-Duet1			[15]
pCR-cloning1 [Kn]	Repeats cloned into pCOLA-Duet1	NcoI/PacI	TS543/TS544	This study
**Expression**				
pCd-H [Str]	Cascade cassette (*cas8f1-cas5f1-cas7f1* and *cas6f1* with N-terminal His_6 sequence). Cascade complex purification.			[15]
pCas2/3-H [Amp]	*cas2/3* gene in pET-SH; C-terminus of Cas2/3 is fused with His_6 tag. Cas2/3 purification.			[15]
pCR-NT [Cm]	CRISPR region; spacer targeting sequence in pSP plasmid. In was used for expression of Cascade complex (annotated as pCR in the source study), which was used for EMSA, cleavage and AFM experiments. This plasmid (annotaded as pCR-NT) was also used in the *in vivo* experiment as non-targeting control.			[15]
pCR-T [Cm]	CRISPR region; spacer targeting *lacZ* gene in *E. coli* genome. *In vivo* experiment.	SapI	TS747/TS748	This study
pCR-λ [Kn]	CRISPR locus containing λ phage DNA targeting spacer. Cloned into pCR-cloning1 plasmid. Purification of Cascade complex, which was used in the single molecul experiment of Soft DNA Curtains.	SapI	TS1138 /TS1139	This study
pCd-C1-2/3 [Kn]	Cascade cassette (*cas8f1-cas5f1-cas7f1-cas6f1)* and *cas1cas3* cloned in the pCOLA-Duet1. *In vivo* experiment.			This study
pAvi-His-Cd [Str]	Cascade cassette (*cas8f1-cas5f1-cas7f1* and *cas6f* with N-terminal His_6 and AviTag sequences). AviTag was inserted into the pCd-H plasmid. Purification of Cascade complex, which was used in the single molecul experiment of Soft DNA Curtains.	NcoI	MZ1209/MZ1210	This study

pACYC-BirA [Cm]	Biotin ligase. *In vivo* biotinylation of AviTag. Purification of biotinylated Cascade complex, which was used in the single molecul experiment of Soft DNA Curtains.			
pCDF-AcrIF1 [Str]	*acrIF1* from *P. aeruginosa* JBD30 phage	NcoI/XhoI	TS918/TS919	This study
pCDF-AcrIF2 [Str]	*acrIF2* from *P. aeruginosa* D3112 phage	NcoI/XhoI	TS918/TS919	This study
pCDF-AcrIF3 [Str]	*acrIF3* from *P. aeruginosa* JBD5 phage	NcoI/XhoI	TS918/TS919	This study
pCDF-AcrIF4 [Str]	*acrIF4* from *P. aeruginosa* JBD26 phage	NcoI/XhoI	TS918/TS919	This study
pCDF-AcrIF5 [Str]	*acrIF5* from *P. aeruginosa* JBD5 phage	NcoI/XhoI	TS918/TS919	This study
pCDF-Os-AcrIF6 [Str]	*acrIF6* from *O. smirnovii*	NcoI/XhoI	TS918/TS919	This study
pCDF-Pa-AcrIF6 [Str]	*acrIF6* from *P. aeruginosa*	NcoI/XhoI	TS918/TS919	This study
pCDF-AcrIF7 [Str]	*acrIF7* from *P. aeruginosa*	NcoI/XhoI	TS918/TS919	This study
pCDF-Ds-AcrIF8 [Str]	*acrIF8* from *Delftia* sp. 670	NcoI/XhoI	TS918/TS919	This study
pCDF-Pp-AcrIF8 [Str]	*acrIF8* from *Pectobacterium* ZF40 phage	NcoI/XhoI	TS918/TS919	This study
pCDF-Aa1-AcrIF9 [Str]	*acrIF9* from *A. actinomycetemcomitans serotype* e str. SC1083	NcoI/XhoI	TS918/TS919	This study
pCDF-Aa2-AcrIF9 [Str]	*acrIF9* from *A. actinomycetemcomitans* serotype b str. SCC1398	NcoI/XhoI	TS918/TS919	This study
pCDF-Aa3-AcrIF9 [Str]	*acrIF9* from *A. actinomycetemcomitans* D11S-1	NcoI/XhoI	TS918/TS919	This study
pCDF-Vp-AcrIF9-Vp [Str]	*AcrIF9* from *V. parahaemolyticus*	NcoI/XhoI	TS918/TS919	This study
pCDF-AcrIF10 [Str]	*AcrIF10* from *S. xiamenensis*	NcoI/XhoI	TS918/TS919	This study
pCDF-His-Os-AcrIF6 [Str]	*AcrIF6-Osm* with N-terminal His_6 sequence	BamHI/XhoI	TS918/TS919	This study
pCDF-His-Vp-AcrIF9 [Str]	*Vp-AcrIF* with N-terminal His_6 sequence	BamHI/XhoI	TS918/TS919	This study
pCDF-His-Aa1-AcrIF9 [Str]	*Aa1-AcrIF9* with N-terminal His_6 sequence	BamHI/XhoI	TS918/TS919	This study
pCDF-His-Aa2-AcrIF9 [Str]	*Aa2-AcrIF9* with N-terminal His_6 sequence	BamHI/XhoI	TS918/TS919	This study
pCDF-His-Aa3-AcrIF9 [Str]	*Aa3-AcrIF9* with N-terminal His_6 sequence	BamHI/XhoI	TS918/TS919	This study
pCDF-His-Aa1-AcrIF9-K24A/R37A/K72A [Str]	*AcrIF9-Aa1- K24A/R37A/K72A* with N-terminal His_6 sequence; putative DNA interaction surface [M1]	BamHI/XhoI	TS918/TS919	This study
pCDF-His-Aa1-AcrIF9-K24E [Str]	*AcrIF9-Aa1-K24E* with N-terminal His_6 sequence; putative DNA interaction surface [M2]	BamHI/XhoI	TS918/TS919	This study
pCDF-His-Aa1-AcrIF9-R37E [Str]	*AcrIF9-Aa1- R37E* with N-terminal His_6 sequence; putative DNA interaction surface [M3]	BamHI/XhoI	TS918/TS919	This study

pCDF-His-Aa1-AcrIF9-N12A/N13A/N15A/ Y17A [Str]	*AcrIF9-Aa1- N12A/N13A/N15A/ Y17A* with N-terminal His_6 sequence; putative Cascade interaction surface [M4]	BamHI/XhoI	TS918/TS919	This study
pCDF-His-Aa1-AcrIF9-F41A [Str]	*AcrIF9-Aa1- F41A* with N-terminal His_6 sequence; putative Cascade interaction surface [M5]	BamHI/XhoI	TS918/TS919	This study
pCDF-His-AcrIF9-Aa1-Δ(L54-V60) [Str]	*AcrIF9-Aa1-Δ(L54-V60)* with N-terminal His_6 sequence; loop deletion [M6]	BamHI/XhoI	TS918/TS919	This study
pCDF-His-AcrIF9-Aa1-I51-D61->VNGL [Str]	*AcrIF9-Aa1-I51-D61->VNGL* with N-terminal His6 sequence; loop replacement with sequence of Vp-AcrIF9 loop [M7]	BamHI/XhoI	TS918/TS919	This study
**Substrate production**				
pSP [Amp]	CC PAM and target protospacer sequences in pUC19 (pSP1-CC). Template for production of SP1, SP2 and SP3 DNA substrates (Supplementary Table S7)			[16]
pNS [Amp]	AA PAM and non-target protospacer sequences in pUC19 (pSP3-AA). Template for production of NS DNA substrate (Supplementary Table S7)			[16]

[Str] streptomycin resistance, [Kn] kanamycin resistance, [Cm] chloramphenicol resistance, [Amp] ampicillin resistance

Supplementary Table S6. Sequences of AcrIF genes used in this study.

Anti-CRISPR	DNA sequence
AcrIF1 (30-35)	atgaagttcatcaaatacctcagcaccgctcacctgaactatatgaatatcgccgtttacgaaaatggcagcaaaatcaaagcccgcgttgagaacgtcgtaaacggcaaaagcgttggtgctcgtgattttgactcaacggagca actggaatcctggttttatggtctgcctggcagtggcctcggtcgtattgaaaacgctatgaatgagatttcccggcgtgaaaacccc
AcrIF2 (3112-30)	atgatcgctcagcagcacaaagatactgtcgcagcgtgcgaagccgccgaagccatcgctatcgctaaagatcaggtatgggatggcgagggctataccaagtacacgttcgacgacaacagcgtcctgatccagtcgggca ctactcagtatgcgatggatgccgacgacgcagacagcatcaaaggctatgcggactggctggacgatgaggctcgctccgccgaagcgtcggagatcgagcgcctgcttgaatcggtcgaggaggag
AcrIF3 (5-35)	atgagcaacacgatttcagatcgcatcgtcgcccgctcggttattgaagcggctcgattcatccagtcctgggaagatgcagaccccgacagcctgactgaagaccaggtcctggccgccgctggttttgccgccaggctccat gaggggctccaggctaccgtcctgcaacggctggtggacgagtccaatcatgaagagtatcgcgagttcaaggcatgggaagaggcgctgctcaacgcagatgggcgggtcgcgagcagcccgtttgctgattggggatg gtggtatcgcatcgccaacgtgatgctggccactgcctcgcagaacgtaggtgtcacctggggaagccgcgtccatgggcgtttaatggctatttttcaggacaagttcaagcagcggtatgaggaacaggca
AcrIF4 (26-37)	atgatgaccatcagtaagacagacattgactgttacctccagacctacgttgttatcgacccagtaagcaacgggtggcagtggggaatcgatgagaatggagtcggtggcgccctccatcatggccgagtagagatggttgaa ggtgagaatggttacttcggcttgcgtggcgccactcacccgaccgagaaggaagccatggccgccgcgctcgggtatctgtggaaatgccgacaggacctggtggccatcgctcgcaatgatgcaattgaagccgagaaat accgtgccaaggcc
AcrIF5 (5-36)	atgagccgaccaacagtcgttacggtgacggaaacccccaggaatccgggaagctacgaggtcaacgtagagcgggatggcaaaatggtcgttggccgggcccgcgcgggaagcgatcccggcgcagctgcggcgaa ggccatgcagatggccatggagtgggggagcccgaactacgtcattctcggcagcaacaaggttcttgcgttcataccggagcaactgcgggtgaaaatg
Os-AcrIF6	atgactatctatctcagcaacgctatcgaaaacgccacttccattgaacaagtcgttgaactgatcaatgaaggaacttgcgagggaatggaaggcattgagttcactgccgatatacttgctggtcaatacgcctggagtgccgct aaagattttgttgattcggcaggcatgacagctgatgacctagaagcccagcttgagttcctaagcgccgccggcgcagtattcagcgagagcgtcgcagtagatcatggcttgaaactggccgccgccgacaccgaataa
Pa-AcrIF6	atgaaagtccccgcattcttcgctgctaatatcctgaccatcgagcaaatcattgaagcgatcaacaatgacggaagcgctatgaccagcgcaccggaaatcgccggctactacgcctgggatgccgccactgacgcactgga aagcgagaatgatcttgagcaactgacggaggatgacttcgttgctcatctggaagttctggaagaaagaggcgccaagatcgaccgcgatgcagccatcgcagttgcgctccaattccaggccgcagcagtgaacgacctg cattctggtgacgaatga
AcrIF7	atgaccaccttcaccagcatcgttaccactaaccctgatttcggcggattcgagttctacgttgaagctggacagcagttcgacgattcagcatatgaagaggcatacggtgttagcgttccttccgccgtggtcgaggaaatgaa cgctaaagcggcgcagttgaaagacggcgagtggcttaatgtctcgcatgaagcctga

De-AcrIF8	atgtcctacctcatcagcaacgggcagatcatcgccaagacctccgccaccctggccagcggccaaagcgttgcattcagcggcacccgcgtggccgaggaaatcaatgccggccgccagtggaacctggtcaacgcccagcacgccaccatcggcaacggcaagacctgggatgccaagtgggccaagctggaaaatggccgcatggccagcgagataaaccccggcgaattcttcattgcctga
Pc-AcIF8	atggctcgaatcgctcctaacgaggattccacaatgtctactgcttacatcatctttaactcatccgtcgcggccgtagttgatactgagatcgctaatggcgctaatgtcacattctcaacagtgaccgttaaagaagaaattaacgcgaaccgtgatttcaatctggttaacgctcagaacgggaaaatctcacgcgcaaaacgctggggaaacgaggcgtcaaaatgtgagtattttggccgagaaataaacccaaccgagtttttcatcaaataa
Vp-AcrIF9	atgaaggccgcatacatcattaaagaagttcagaatatcaattcggagcgcgaaggaacccagatcgaggcaacaagcctttcccaggcaaaacgtatcgcatcaaaagaacagtgctttcatgggactgtcatgcgtatcgagactgtaaacggactgtggttggcatacaaggaggacggaaaacgctgggttgactgtcag
Aa1-AcrIF9	atgacgaacgtagtttattactttacggaaactaataacatcaacgcttatgcgactgcggaggctttgaaagcgcagactcttgcggatgccaaacgcgaagcgtcgcgtcgtcagtgcttccaagggaccacgttaaagattggaacgatctactcactgaacagtgacgggcttctggtagacgagatcaccagtaaagaagatgggaaaaaatgggtcgatcgttac
Aa2-AcrIF9	atgacaaatgtcatttactactttaccgagacaaacaatatcaacgcctataccacggcagaagccttgaaggcgcaaacattagcggatgctaaacgtgaggcgtcacgtcgtcagtgctttcagggaacaaccttaaaaatcgggaccatctactcacttaattcggatgggttgttagtggatgaaatcacatcaaaagaggacgggcgtaagtgggtggatcgctac
Aa3-AcrIF9	atgattaacgagcgccaagagatcaagaaaatgaccacatattatttctctgagacatctaacattaacgcttacgcaaccgccgaagccttgaaagcccagactcttgccgacgctaagcgcgaagctagccgccgccagtgtttccagggtacgacgttgaagattgggacgatttattcattaaattcagacggcttgttggttgacgaaatcacatcgaaggaggacggcaagaaatgggttgaccgttac
AcrIF10	atgactacattccgcattgaaaacgtccgcattgaaacaatcaatgacttcgatatggttaaatttgatctagtaaccgatcttggccgtgttgaattagctgagcatgttaactatgacagcgaaggtgattttaaatctgttgaatacactgacagcaatattcgctacaacatggttgatgaactttgctcagtgtttgatttaactgataagccttcgctaatgcctgcaattgactacgttacgtttgctgaaatcatcgaagcagttgaagaaatgttagaagcataa
**Aa1-AcrIF9 mutant sequences**	
Aa1-AcrIF9-K24A/R37A/K72A	atgacgaacgtagtttattactttacggaaactaataacatcaacgcttatgcgactgcggaggctttggcggcgcagactcttgcggatgccaaacgcgaagcgtcggcgcgtcagtgcttccaaggtaccacgttaaagattggaacgatctactcactgaacagtgacgggcttctggtagacgagatcaccagtaaagaagatgggaaagcgtgggtcgatcgttac
Aa1-AcrIF9-K24E	atgacgaacgtagtttattactttacggaaactaataacatcaacgcttatgcgactgcggaggctttggaagcgcagactcttgcggatgccaaacgcgaagcgtcgcgtcgtcagtgcttccaaggtaccacgttaaagattggaacgatctactcactgaacagtgacgggcttctggtagacgagatcaccagtaaagaagatgggaaaaaatgggtcgatcgttac
Aa1-AcrIF9-R37E	atgacgaacgtagtttattactttacggaaactaataacatcaacgcttatgcgactgcggaggctttgaaagcgcagactcttgcggatgccaaacgcgaagcgtcggaacgtcagtgcttccaaggtaccacgttaaagattggaacgatctactcactgaacagtgacgggcttctggtagacgagatcaccagtaaagaagatgggaaaaaatgggtcgatcgttac
Aa1-AcrIF9-F41A	atgacgaacgtagtttattactttacggaaactaataacatcaacgcttatgcgactgcggaggctttgaaagcgcagactcttgcggatgccaaacgcgaagcgtcgcgtcgtcagtgcgcccaaggtaccacgttaaagattggaacgatctactcactgaacagtgacgggcttctggtagacgagatcaccagtaaagaagatgggaaaaaatgggtcgatcgttac
AcrIF9-Aa1-N12A/N13A/N15A/Y17A	atgacgaacgtagtttattactttacggaaactgcggcgatcgcggctgcggcgactgcggaggctttgaaagcgcagactcttgcggatgccaaacgcgaagcgtcgcgtcgtcagtgcttccaaggtaccacgttaaagattggaacgatctactcactgaacagtgacgggcttctggtagacgagatcaccagtaaagaagatgggaaaaaatgggtcgatcgttac
AcrIF9-Aa1-Δ(L54-V60)	atgacgaacgtagtttattactttacggaaactaataacatcaacgcttatgcgactgcggaggctttgaaagcgcagactcttgcggatgccaaacgcgaagcgtcgcgtcgtcagtgcttccaagggaccacgttaaagattggaacgatctactcagacgagatcaccagtaaagaagatgggaaaaaatgggtcgatcgttactaa
AcrIF9-Aa1-I51-D61->VNGL	atgacgaacgtagtttattactttacggaaactaataacatcaacgcttatgcgactgcggaggctttgaaagcgcagactcttgcggatgccaaacgcgaagcgtcgcgtcgtcagtgcttccaagggaccacgttaaagattggaacggtaaacggactggagatcaccagtaaagaagatgggaaaaaatgggtcgatcgttactaa

Supplementary Table S7. Oligonucleotides used in this study.

Primer	Sequence 5'→3'	Purpose
**Cloning**		
TS543	TATCCATGGCTTCACTGCCGAATAGGCAGCTTAGAAATGAAGAGCCGAGCTCGGCGCGCCTGCAGGTCGACAAGC	pCR-cloning construction via NcoI/PacI sites; forward primer
TS544	CTCTTAATTAATTTCTAAGCTGCCTATTCGGCAGTGAAGTGAAGAGCGCTTGTCGACCTGCAGGCGCGCCGAGCTCG	pCR-cloning construction via NcoI/PacI sites; reverse primer
TS918	GAAGTGCCATTCCGCCTGACC	Amplification of *acrIF1-10* genes from synthetic fragment; forward primer
TS919	CACTGAGCCTCCACCTAGCCT	Amplification of *acrIF1-10* genes from synthetic fragment; reverse primer

MZ1209	CATGTCCGGCCTGAACGACATCTTCGAGGCTCAGAAAATC GAATGGCACGAAGGCGGCGG	Annealed oligos were used for AviTag sequence insertion via NcoI site
MZ1210	CATGCCGCCGCCTTCGTGCCATTCGATTTTCTGAGCCTCG AAGATGTCGTTCAGGCCGGA	
TS747	AAAAGATGATCACACTCGGGTGATTACGATCGCGC	Annealed oligos were used for *lacZ* targeting spacer insertion via SapI site
TS748	AAGGCGCGATCGTAATCACCCGAGTGTGATCATCT	
TS1138	AAAATGTACCGGGCGATGTTATTGAAATCTGCGAT	Annealed oligos were used for λ targeting spacer insertion via SapI site
TS1139	AAGATCGCAGATTTCAATAACATCGCCCGGTACAT	
**EMSA**		
Lgur	GCGAGGAAGCGGAAGAGCGCCC	SP1 or NS 420 bp substrate obtained by PCR-amplification from pSP or pNS plasmids as templates, respectively.
pUC57-For	GCCAGGGTTTTCCCAGTCACGA	
**Cas2/3 cleavage**		
GG222	CTTGAGATCCTTTTTTTCTGC	SP2 1444 bp substrate obtained by PCR-amplification from pSP plasmid as a template.
TS759	GAGCAGACAAGCCCGTCAG	
**DNA footprinting and competitive DNA binding**		
TS931*	GCTCGGTACCCGACCACCCTTTTTGATATCC	SP* 272 bp substrate obtained by PCR-amplification from pSP plasmid as a template.
TS930	GCGGGCAGTGAGCGCAACG	
**AFM**		
TS1144	TCCCCTGATTCTGTGGATAACC	SP3 638 bp substrate obtained by PCR-amplification from pSP plasmid as a template.
pUC-Ehe	CCGCATCAGGCGCCATTCGCC	

* ^{32}P-5'-end- labelled

Supplementary References

1. Tutkus, M., Rakickas, T., Kopūstas, A., Ivanovaite, S. N., Venckus, O., Navikas, V., Zaremba, M., Manakova, E. & Valiokas, R. N. (2019) Fixed DNA Molecule Arrays for High-Throughput Single DNA-Protein Interaction Studies, *Langmuir : the ACS journal of surfaces and colloids.* **35**, 5921-5930.
2. Kopūstas, A., Ivanovaitė, Š., Rakickas, T., Pocevičiūtė, E., Paksaitė, J., Karvelis, T., Zaremba, M., Manakova, E. & Tutkus, M. (2020) Oriented Soft DNA Curtains for Single Molecule Imaging, *bioRxiv*, 2020.06.15.151662.
3. Chivers, C. E., Crozat, E., Chu, C., Moy, V. T., Sherratt, D. J. & Howarth, M. (2010) A streptavidin variant with slower biotin dissociation and increased mechanostability, *Nature methods.* **7**, 391-3.
4. Schneider, S. W., Larmer, J., Henderson, R. M. & Oberleithner, H. (1998) Molecular weights of individual proteins correlate with molecular volumes measured by atomic force microscopy, *Pflugers Archiv : European journal of physiology.* **435**, 362-7.
5. Pawluk, A., Staals, R. H., Taylor, C., Watson, B. N., Saha, S., Fineran, P. C., Maxwell, K. L. & Davidson, A. R. (2016) Inactivation of CRISPR-Cas systems by anti-CRISPR proteins in diverse bacterial species, *Nature microbiology.* **1**, 16085.
6. Bondy-Denomy, J., Pawluk, A., Maxwell, K. L. & Davidson, A. R. (2013) Bacteriophage genes that inactivate the CRISPR/Cas bacterial immune system, *Nature.* **493**, 429-32.
7. Chowdhury, S., Carter, J., Rollins, M. F., Golden, S. M., Jackson, R. N., Hoffmann, C., Nosaka, L., Bondy-Denomy, J., Maxwell, K. L., Davidson, A. R., Fischer, E. R., Lander, G. C. & Wiedenheft, B. (2017) Structure Reveals Mechanisms of Viral Suppressors that Intercept a CRISPR RNA-Guided Surveillance Complex, *Cell.* **169**, 47-57 e11.
8. Guo, T. W., Bartesaghi, A., Yang, H., Falconieri, V., Rao, P., Merk, A., Eng, E. T., Raczkowski, A. M., Fox, T., Earl, L. A., Patel, D. J. & Subramaniam, S. (2017) Cryo-EM Structures Reveal Mechanism and Inhibition of DNA Targeting by a CRISPR-Cas Surveillance Complex, *Cell.* **171**, 414-426 e12.
9. Peng, R., Xu, Y., Zhu, T., Li, N., Qi, J., Chai, Y., Wu, M., Zhang, X., Shi, Y., Wang, P., Wang, J., Gao, N. & Gao, G. F. (2017) Alternate binding modes of anti-CRISPR viral suppressors AcrF1/2 to Csy surveillance complex revealed by cryo-EM structures, *Cell research.* **27**, 853-864.
10. Zhang, K., Wang, S., Li, S., Zhu, Y., Pintilie, G. D., Mou, T. C., Schmid, M. F., Huang, Z. & Chiu, W. (2020) Inhibition mechanisms of AcrF9, AcrF8, and AcrF6 against type I-F CRISPR-Cas complex revealed by cryo-EM, *Proceedings of the National Academy of Sciences of the United States of America.* **117**, 7176-7182.
11. Gabel, C., Li, Z., Zhang, H. & Chang, L. (2020) Structural basis for inhibition of the type I-F CRISPR-Cas surveillance complex by AcrIF4, AcrIF7 and AcrIF14, *Nucleic acids research*.
12. Bondy-Denomy, J., Garcia, B., Strum, S., Du, M., Rollins, M. F., Hidalgo-Reyes, Y., Wiedenheft, B., Maxwell, K. L. & Davidson, A. R. (2015) Multiple mechanisms for CRISPR-Cas inhibition by anti-CRISPR proteins, *Nature.* **526**, 136-9.
13. Hirschi, M., Lu, W. T., Santiago-Frangos, A., Wilkinson, R., Golden, S. M., Davidson, A. R., Lander, G. C. & Wiedenheft, B. (2020) AcrIF9 tethers non-sequence specific dsDNA to the CRISPR RNA-guided surveillance complex, *Nature communications.* **11**, 2730.

14. Drabavicius, G., Sinkunas, T., Silanskas, A., Gasiunas, G., Venclovas, C. & Siksnys, V. (2018) DnaQ exonuclease-like domain of Cas2 promotes spacer integration in a type I-E CRISPR-Cas system, *EMBO reports.* **19**, e45543.
15. Tuminauskaite, D., Norkunaite, D., Fiodorovaite, M., Tumas, S., Songailiene, I., Tamulaitiene, G. & Sinkunas, T. (2020) DNA interference is controlled by R-loop length in a type I-F1 CRISPR-Cas system, *BMC biology.* **18**, 65.
16. Sinkunas, T., Gasiunas, G., Waghmare, S. P., Dickman, M. J., Barrangou, R., Horvath, P. & Siksnys, V. (2013) In vitro reconstitution of Cascade-mediated CRISPR immunity in Streptococcus thermophilus, *The EMBO journal.* **32**, 385-94.

Raw Figures

Figure 2b

AcrIF6

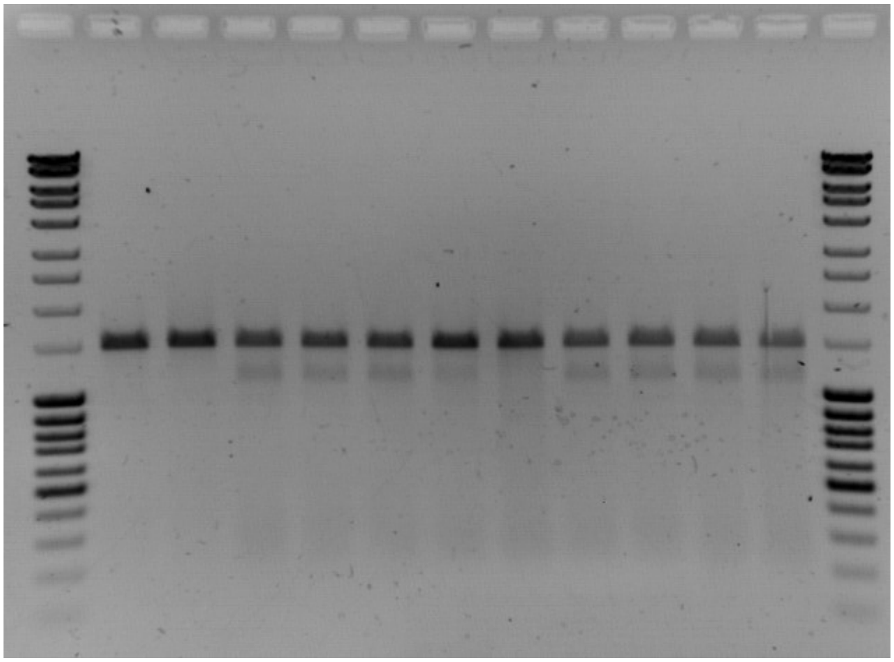

AcrIF9

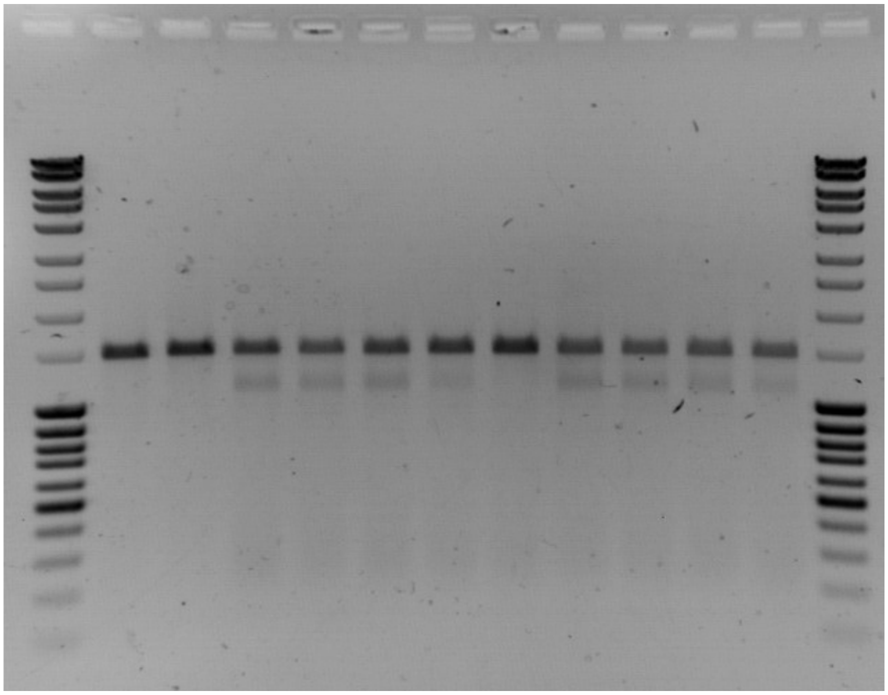

Figure 2c

AcrIF6

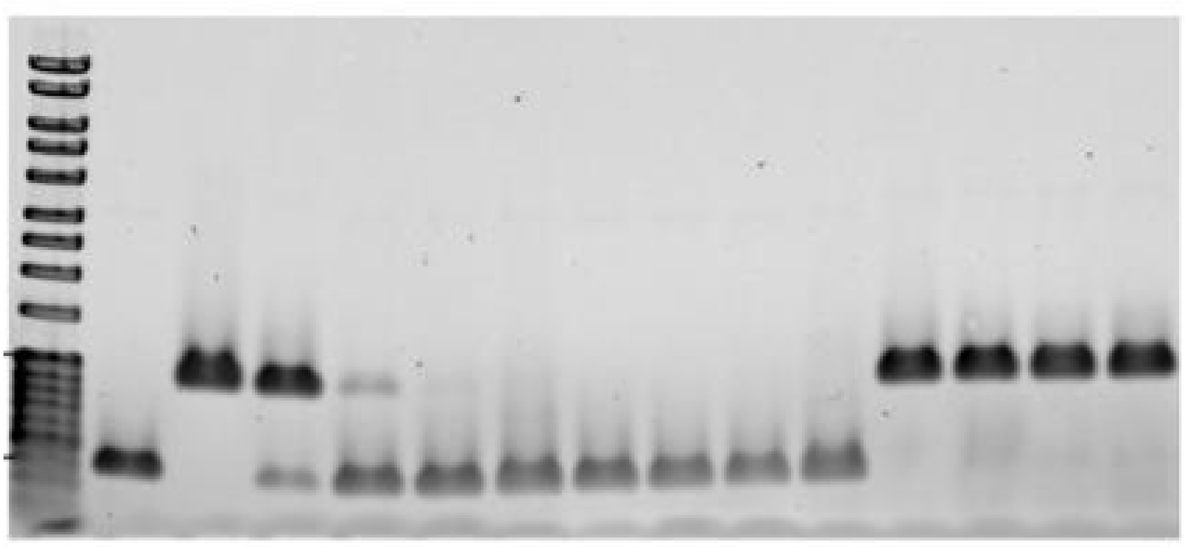

AcrIF9

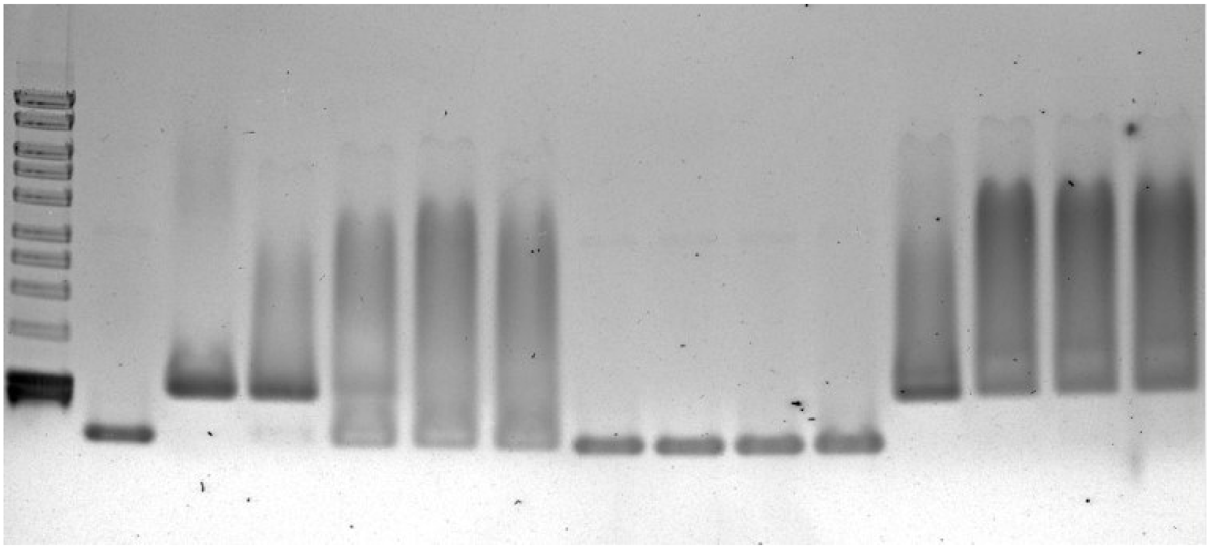

Figure 2d

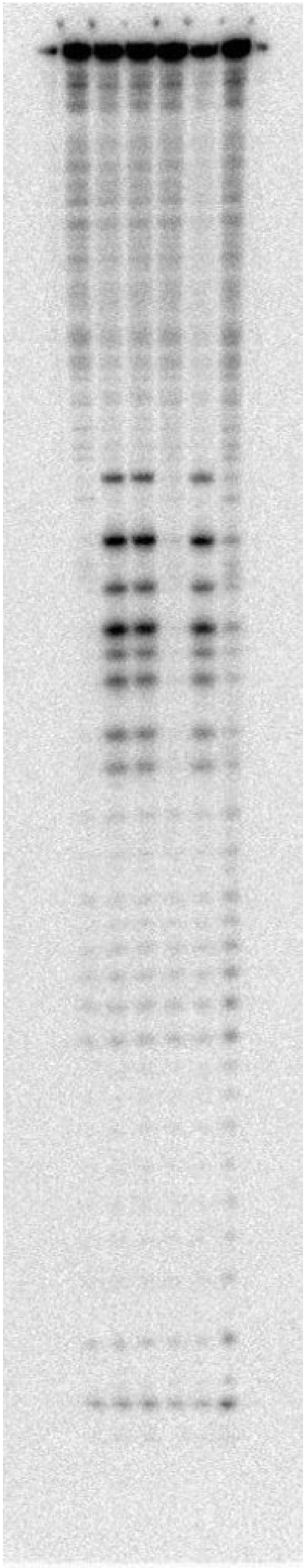

Figure 5c

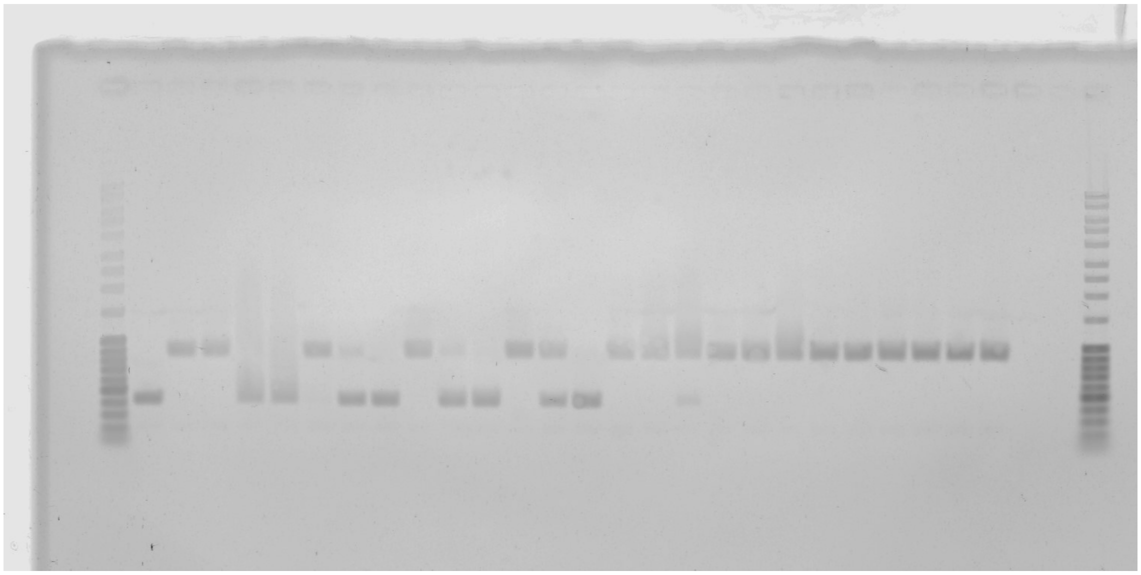

Figure 5d

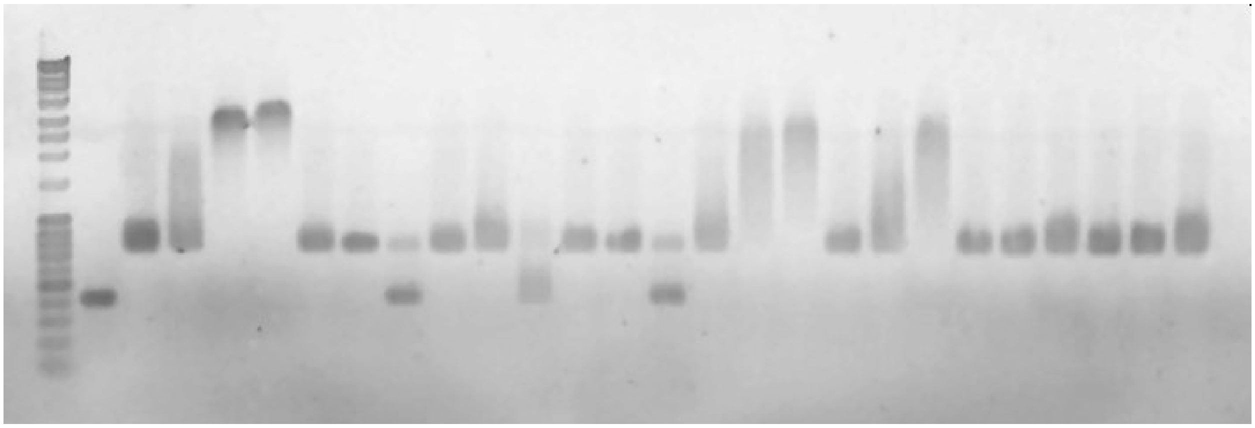

Supplementary Figure S2

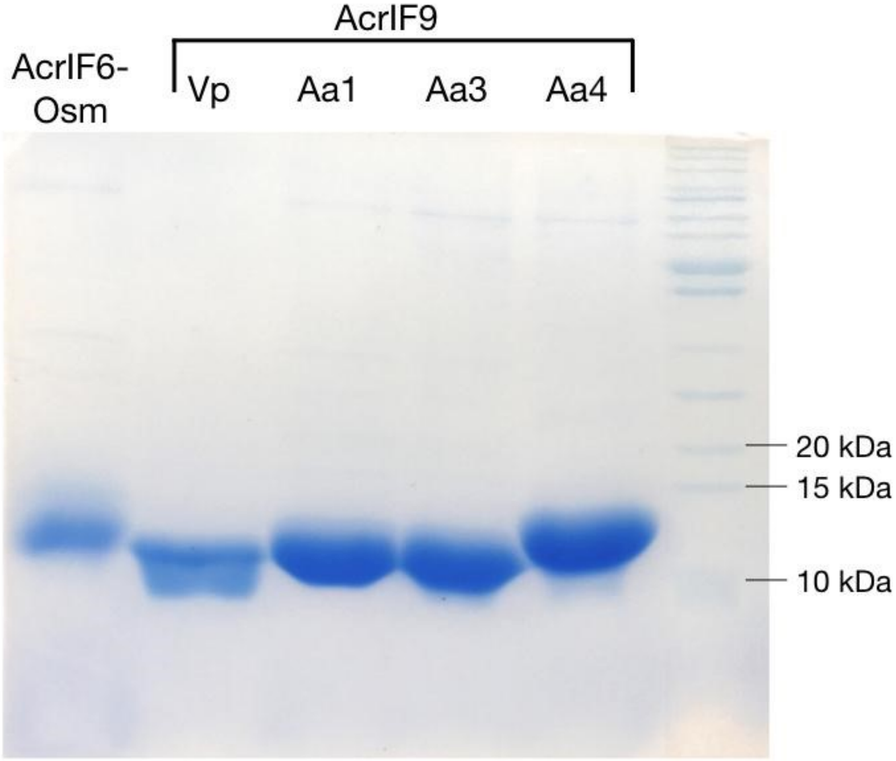

Supplementary Figure S3b

AcrIF6

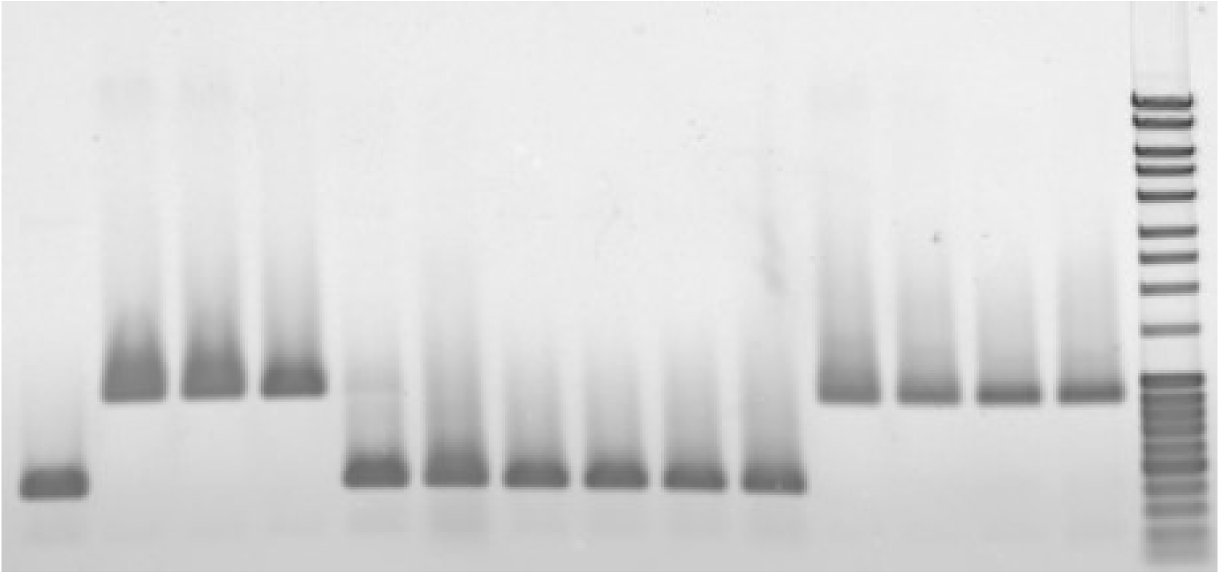

AcrIF9

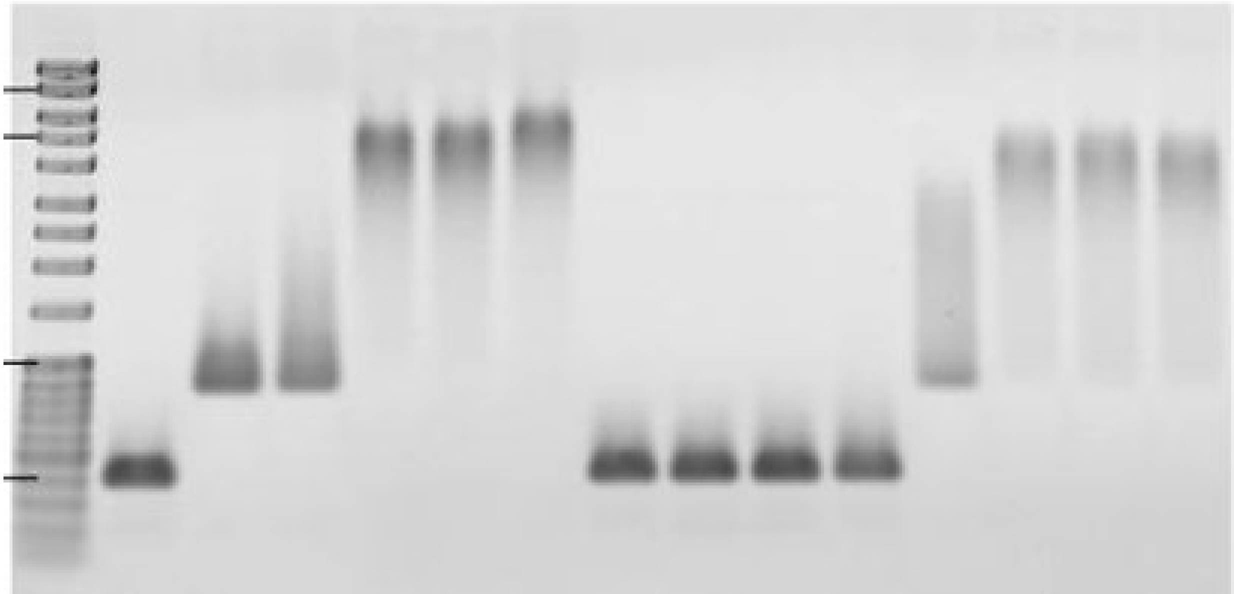

Supplementary Figure S3c

AcrIF6

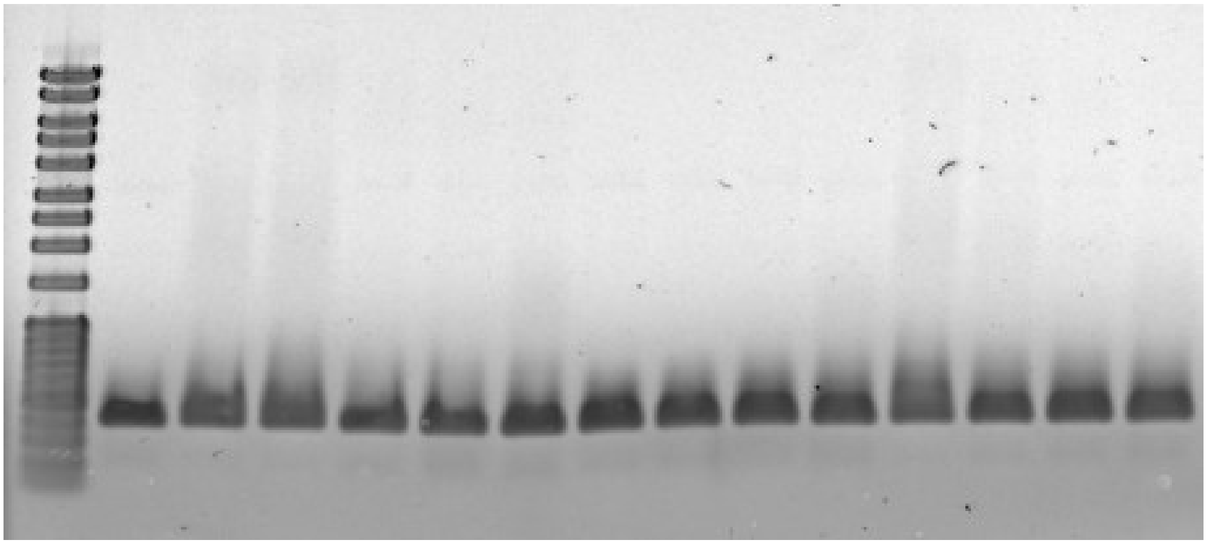

AcrIF9

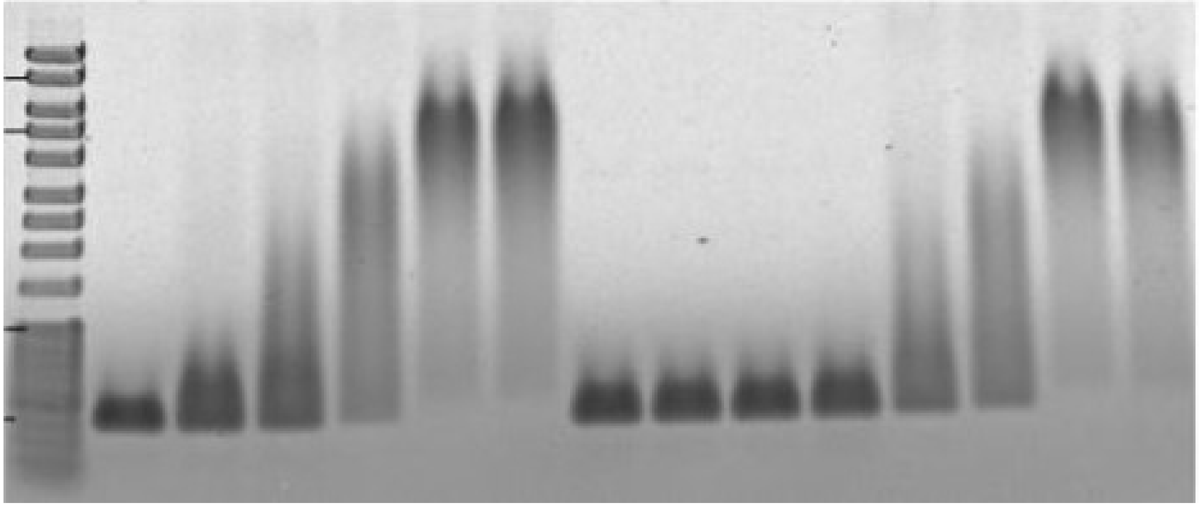

Supplementary Figure 4b

Left panel

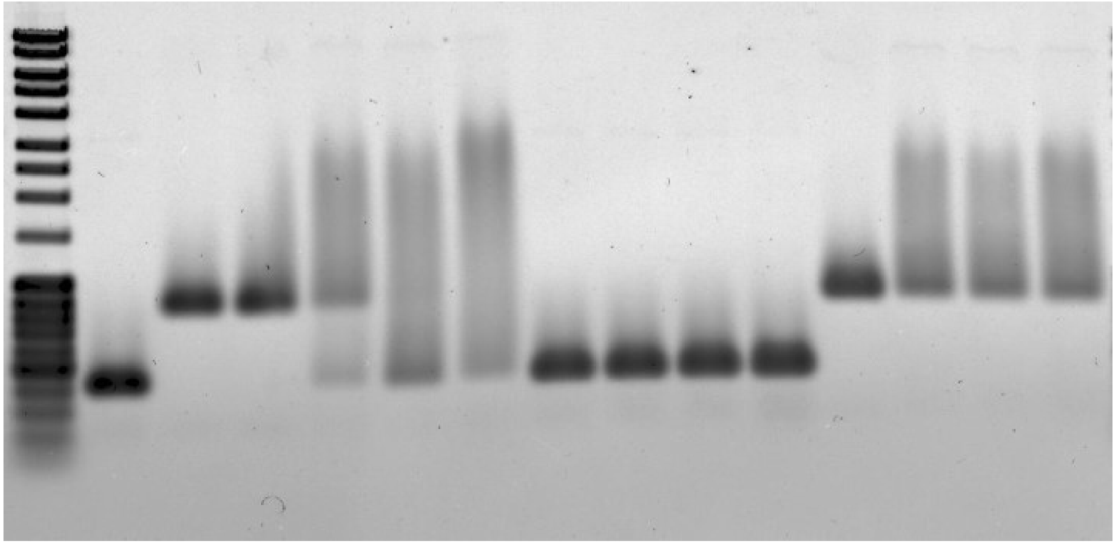

Right panel

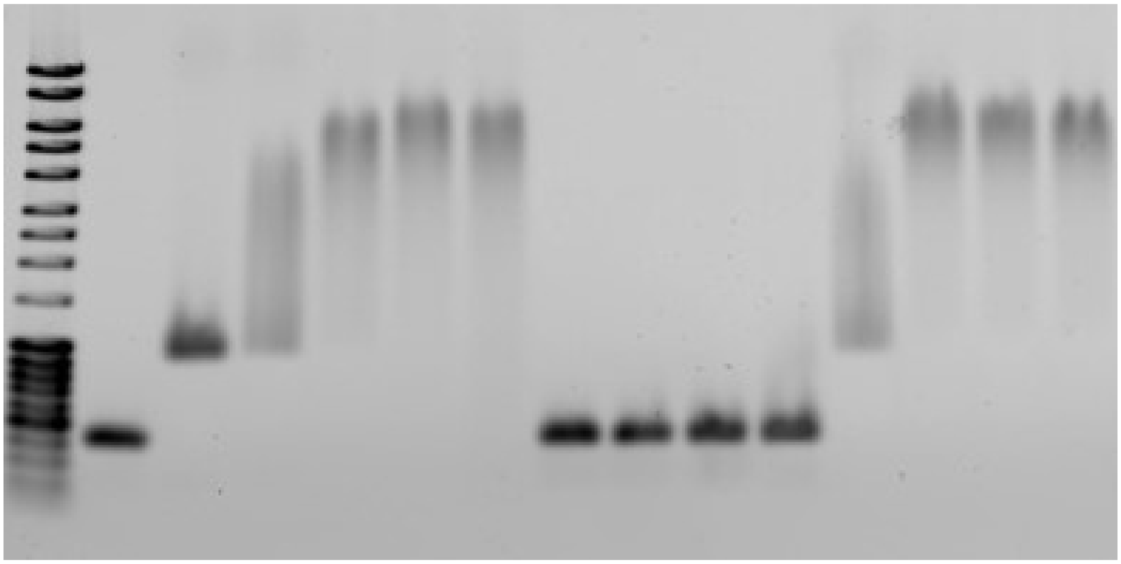

Supplementary Figure S4c

Left panel

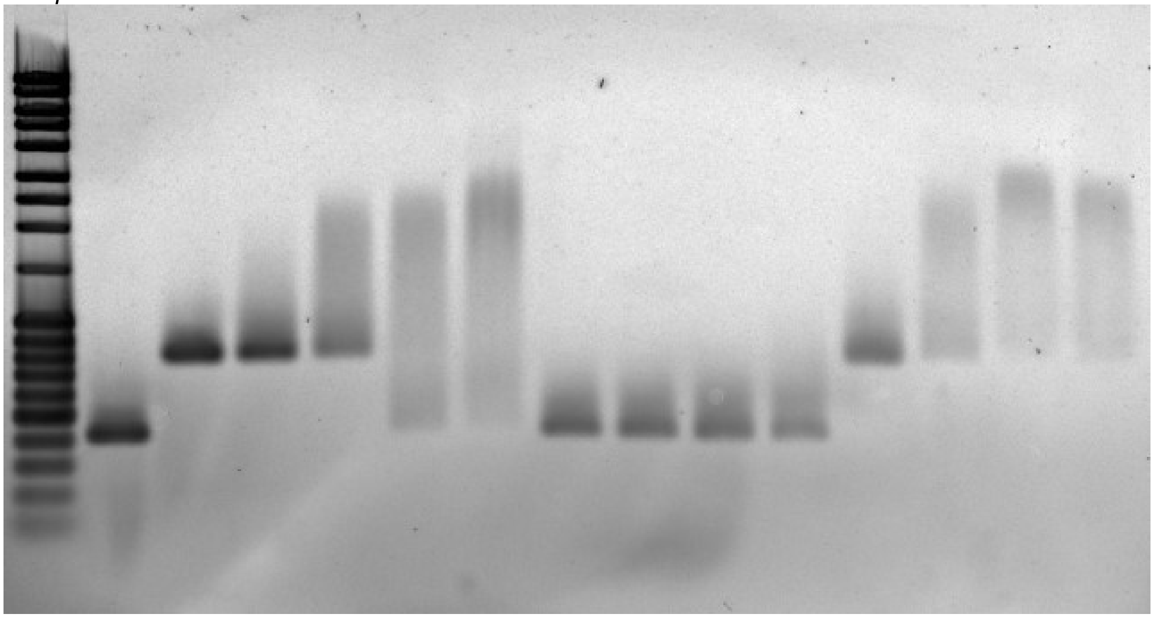

Right panel

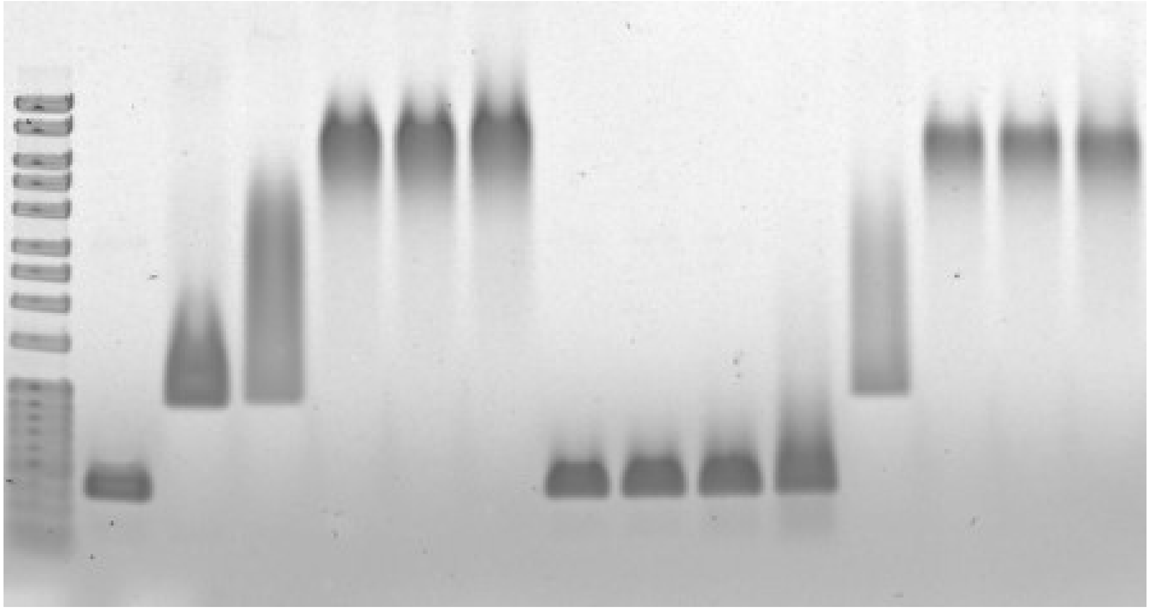

Supplementary Figure S4d

Left panel

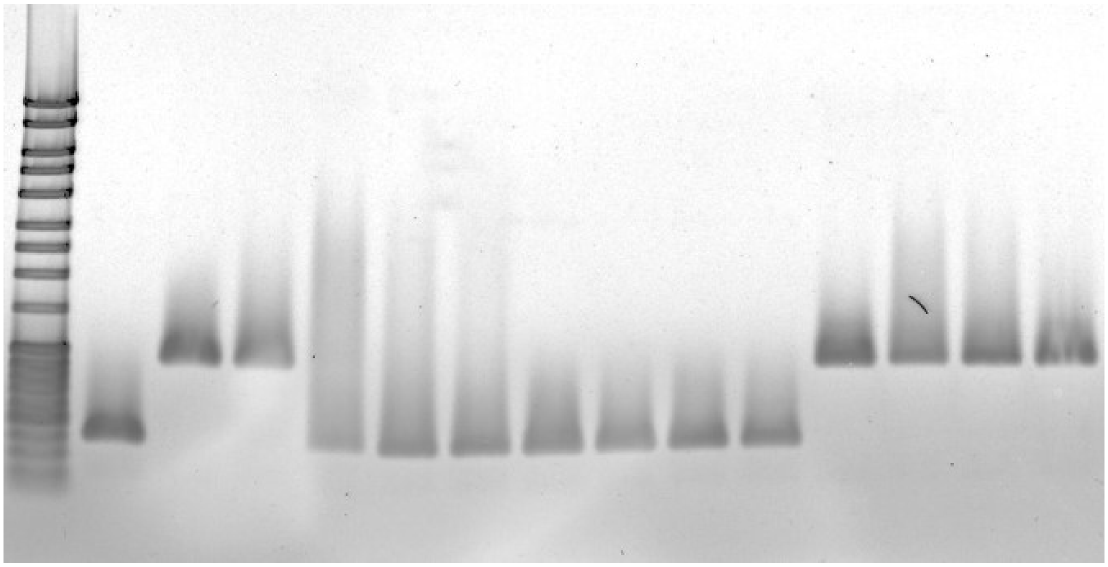

Right panel

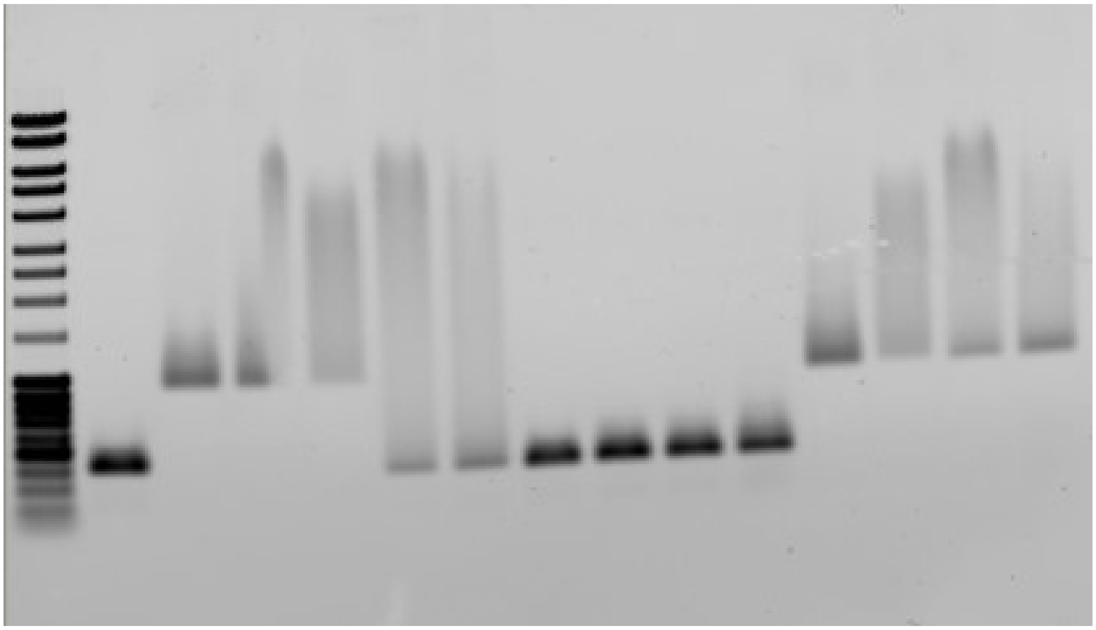

Supplementary Figure S5d

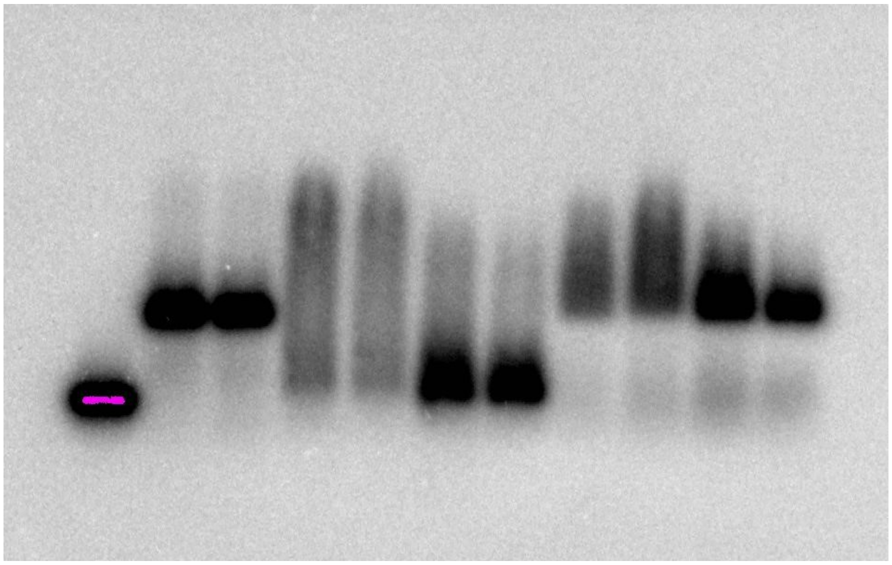

Supplementary Figure S6

AcrIF9 and AcrIF6 cropped lanes are indicated by red and orange rectangles, respectively.

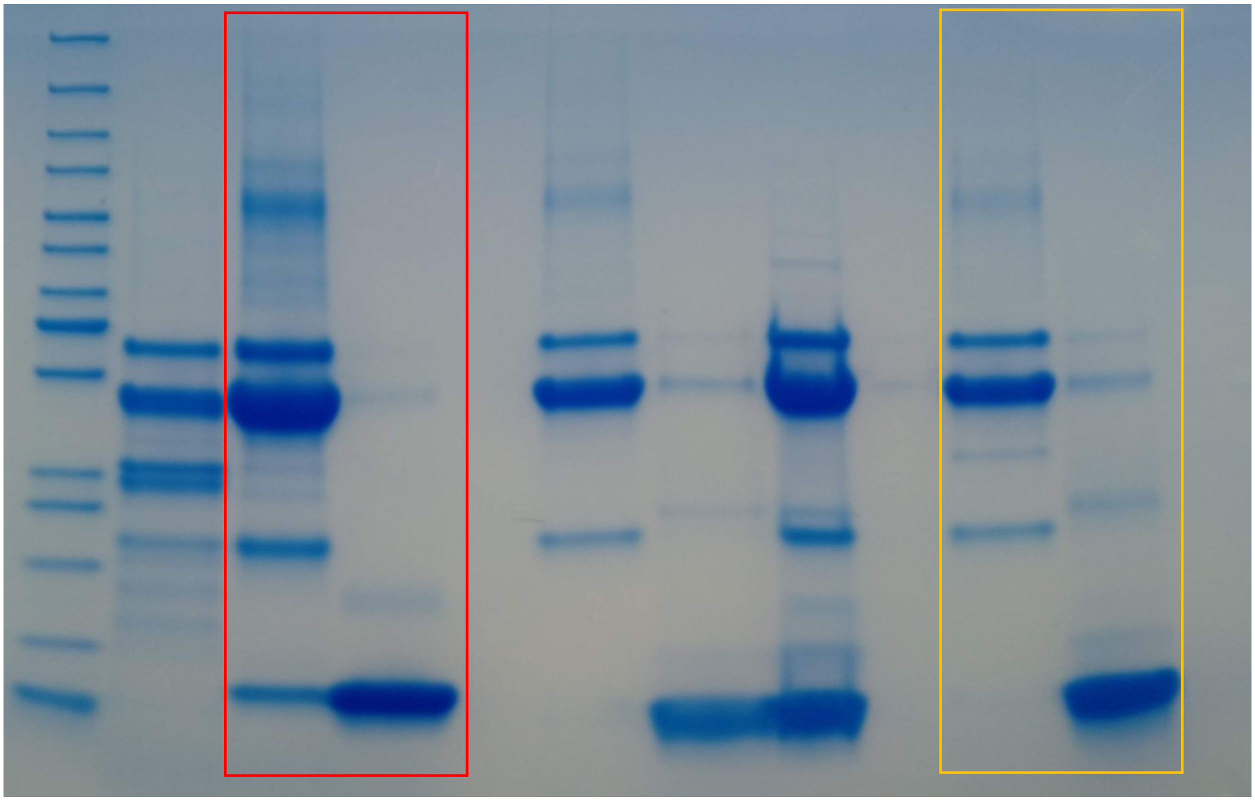

Supplementary Figure S11

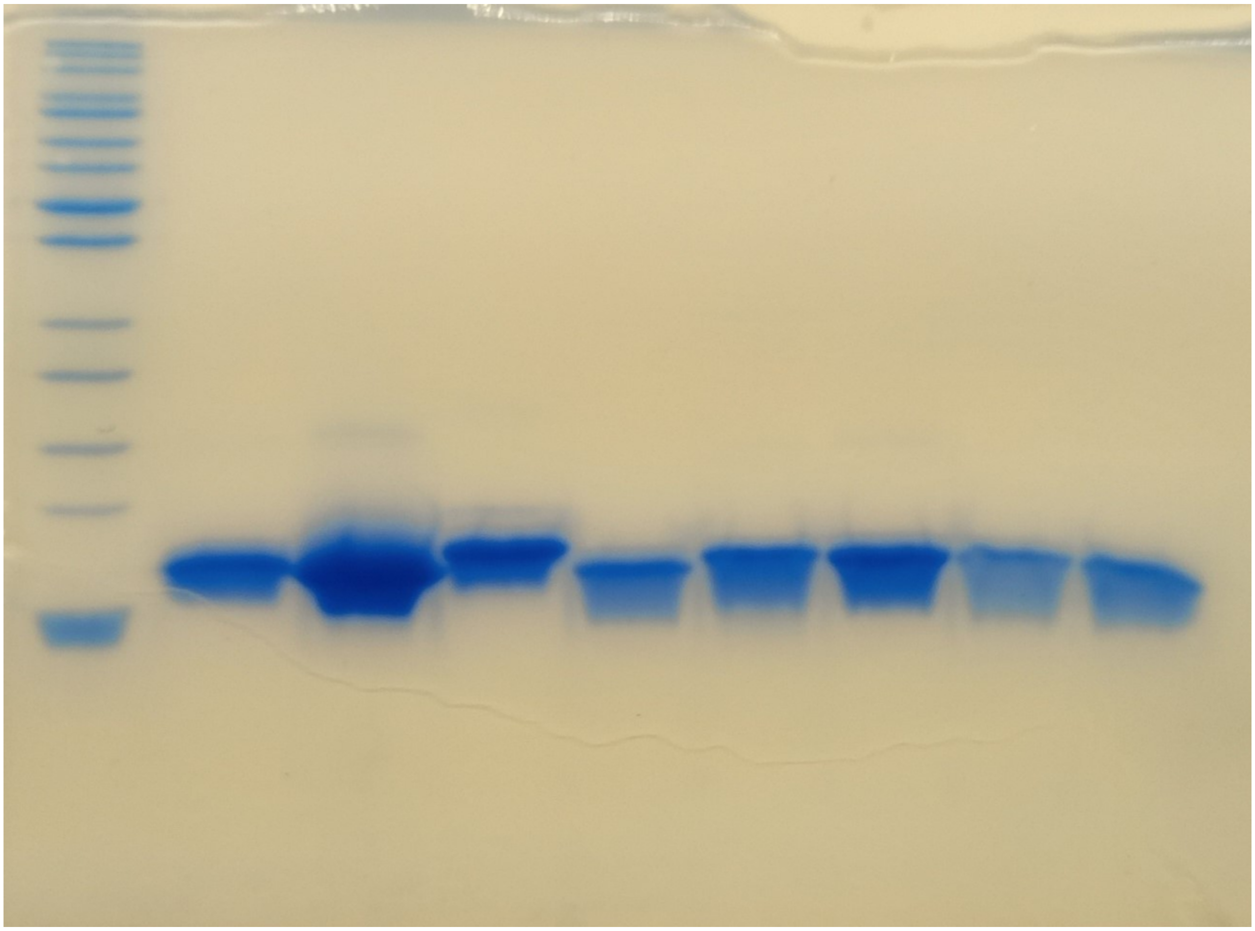

Supplementary Figure S12

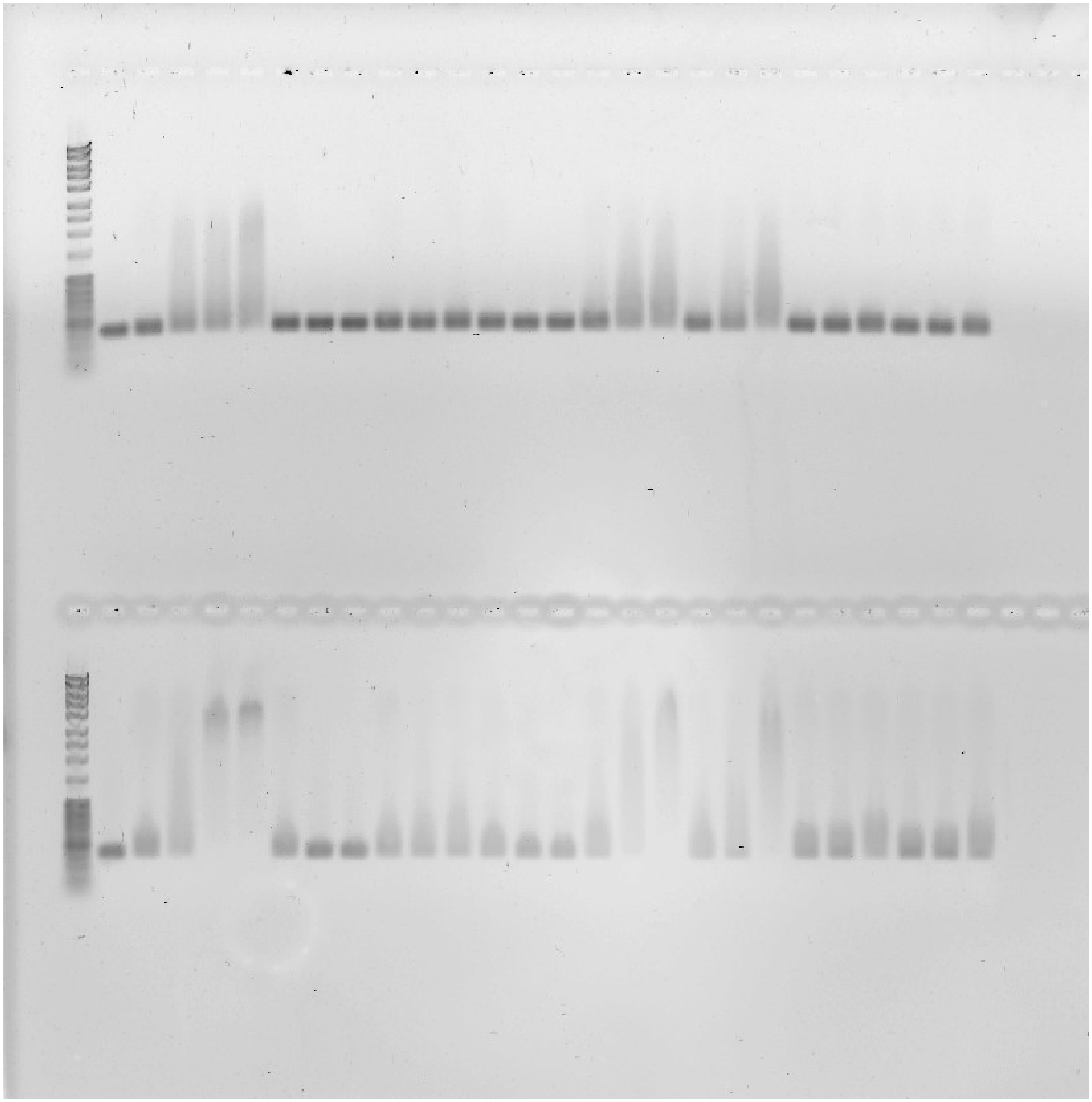

Supplementary Figure S13

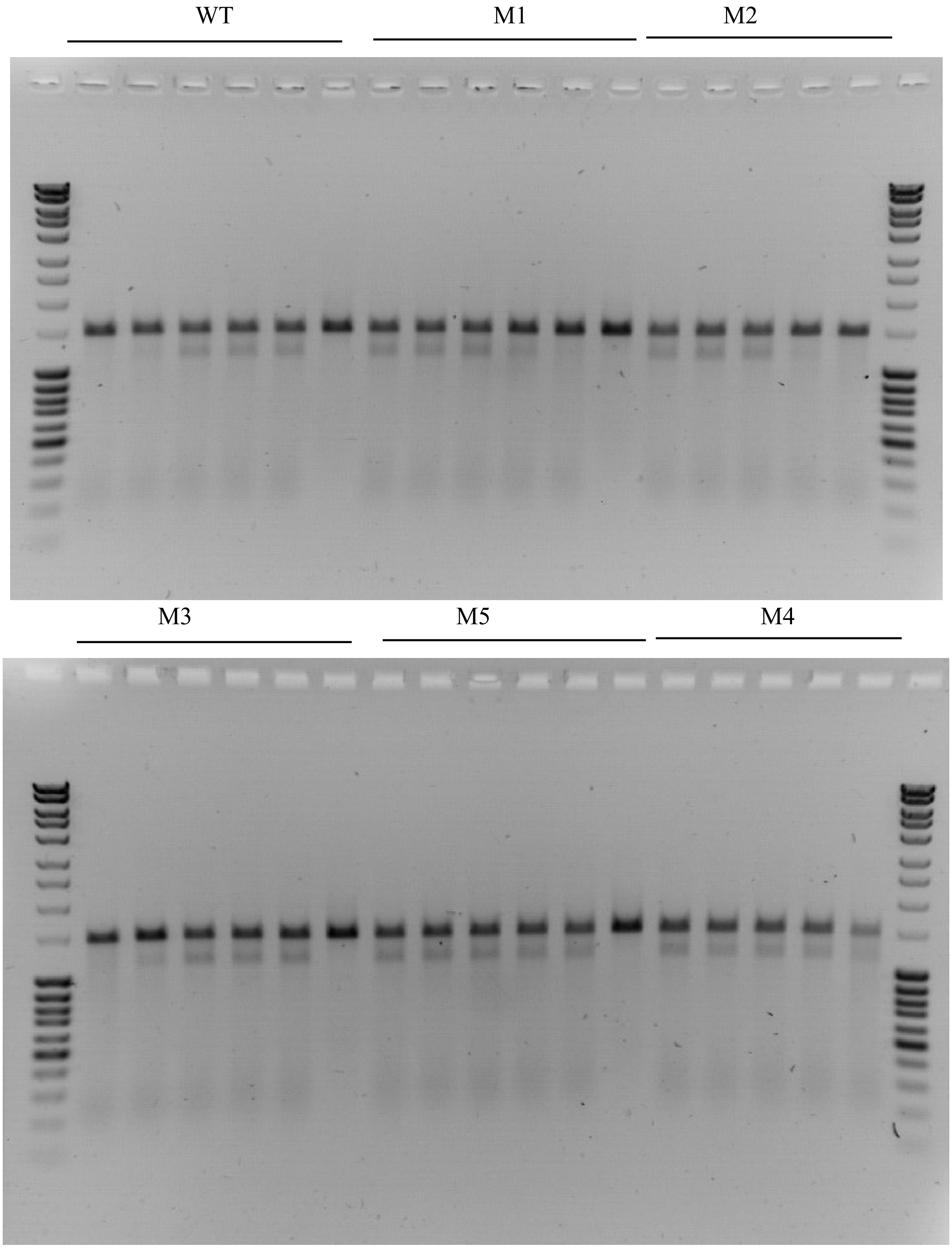

Supplement: Supplementary file 1 — Supplementary Information. [file 41598_2022_19797_MOESM1_ESM.pdf]
